# Qualitative longitudinal research in health research: a method study

**DOI:** 10.1186/s12874-022-01732-4

**Published:** 2022-10-01

**Authors:** Åsa Audulv, Elisabeth O. C. Hall, Åsa Kneck, Thomas Westergren, Liv Fegran, Mona Kyndi Pedersen, Hanne Aagaard, Kristianna Lund Dam, Mette Spliid Ludvigsen

**Affiliations:** 1 Department of Nursing, Umeå University, Umeå, Sweden; 2 Faculty of Health, Aarhus University, Aarhus, Denmark; 3 Faculty of Health Sciences, University of Faroe Islands, Thorshavn, Faroe Islands Denmark; 4 Department of Health Care Sciences, Ersta Sköndal Bräcke University College, Stockholm, Sweden; 5 Department of Health and Nursing Science, University of Agder, Kristiansand, Norway; 6 Department of Public Health, University of Stavanger, Stavanger, Norway; 7 Center for Clinical Research, North Denmark Regional Hospital, Hjørring, Denmark; 8 Department of Clinical Medicine, Aalborg University, Aalborg, Denmark; 9 Lovisenberg Diaconale Univeristy of College, Oslo, Norway; 10 Department of Clinical Medicine-Randers Regional Hospital, Aarhus University, Aarhus, Denmark; 11 Faculty of Nursing and Health Sciences, Nord University, Bodø, Norway

**Keywords:** Qualitative longitudinal research, Method development, Repeated data collection

## Abstract

**Background:**

Qualitative longitudinal research (QLR) comprises qualitative studies, with repeated data collection, that focus on the temporality (e.g., time and change) of a phenomenon. The use of QLR is increasing in health research since many topics within health involve change (e.g., progressive illness, rehabilitation). A method study can provide an insightful understanding of the use, trends and variations within this approach. The aim of this study was to map how QLR articles within the existing health research literature are designed to capture aspects of time and/or change.

**Methods:**

This method study used an adapted scoping review design. Articles were eligible if they were written in English, published between 2017 and 2019, and reported results from qualitative data collected at different time points/time waves with the same sample or in the same setting. Articles were identified using EBSCOhost. Two independent reviewers performed the screening, selection and charting.

**Results:**

A total of 299 articles were included. There was great variation among the articles in the use of methodological traditions, type of data, length of data collection, and components of longitudinal data collection. However, the majority of articles represented large studies and were based on individual interview data. Approximately half of the articles self-identified as QLR studies or as following a QLR design, although slightly less than 20% of them included QLR method literature in their method sections.

**Conclusions:**

QLR is often used in large complex studies. Some articles were thoroughly designed to capture time/change throughout the methodology, aim and data collection, while other articles included few elements of QLR. Longitudinal data collection includes several components, such as what entities are followed across time, the tempo of data collection, and to what extent the data collection is preplanned or adapted across time. Therefore, there are several practices and possibilities researchers should consider before starting a QLR project.

**Supplementary Information:**

The online version contains supplementary material available at 10.1186/s12874-022-01732-4.

## Background

Health research is focused on areas and topics where time and change are relevant. For example, processes such as recovery or changes in health status. However, relating time and change can be complicated in research, as the representation of reality in research publications is often collected at one point in time and fixed in its presentation, although time and change are always present in human life and experiences. Qualitative longitudinal research (QLR; also called longitudinal qualitative research, LQR) has been developed to focus on subjective experiences of time or change using qualitative data materials (e.g., interviews, observations and/or text documents) collected across a time span with the same participants and/or in the same setting [1, 2]. QLR within health research may have many benefits. Firstly, human experiences are not fixed and consistent, but changing and diverse, therefore people’s experiences in relation to a health phenomenon may be more comprehensively described by repeated interviews or observations over time. Secondly, experiences, behaviors, and social norms unfold over time. By using QLR, researchers can collect empirical data that represents not only recalled human conceptions but also serial and instant situations reflecting transitions, trajectories and changes in people’s health experiences, personal development or health care organizations [3–5].

### Key features of QLR

Whether QLR is a methodological approach in its own right or a design element of a particular study within a traditional methodological approach (e.g., ethnography or grounded theory) is debated [1, 6]. For example, Bennett et al. [7] describe QLR as untied to methodology, giving researchers the flexibility to develop a suitable design for each study. McCoy [6] suggests that epistemological and ontological standpoints from interpretative phenomenological analysis (IPA) align with QLR traditions, thus making longitudinal IPA a suitable methodology. Plano-Clark et al. [8] described how longitudinal qualitative elements can be used in mixed methods studies, thus creating longitudinal mixed methods. In contrast, several researchers have argued that QLR is an emerging methodology [1, 5, 9, 10]. For example, Thomson et al. [9] have stated “What distinguishes longitudinal qualitative research is the deliberate way in which temporality is designed into the research process, making change a central focus of analytic attention” (p. 185). Tuthill et al. [5] concluded that some of the confusion might have arisen from the diversity of data collection methods and data materials used within QLR research. However, there are no investigations showing to what extent QLR studies use QLR as a distinct methodology versus using a longitudinal data collection as a more flexible design element in combination with other qualitative methodologies.

QLR research should focus on aspects of temporality, time and/or change [11–13]. The concepts of time and change are seen as inseparable since change is happening with the passing of time [13]. However, time can be conceptualized in different ways. Time is often understood from a chronological perspective, and is viewed as fixed, objective, continuous and measurable (e.g., clock time, duration of time). However, time can also be understood from within, as the experience of the passing of time and/or the perspective from the current moment into the constructed conception of a history or future. From this perspective, time is seen as fluid, meaning that events, contexts and understandings create a subjective experience of time and change. Both the chronological and fluid understanding of time influence QLR research [11]. Furthermore, there is a distinction between over-time, which constitutes a comparison of the difference between points in time, often with a focus on the latter point or destination, and through-time, which means following an aspect across time while trying to understand the change that occurs [11]. In this article, we will mostly use the concept of across time to include both perspectives.

Some authors assert that QLR studies should include a qualitative data collection with the same sample across time [11, 13], whereas Thomson et al. [9] also suggest the possibility of returning to the same data collection site with the same or different participants. When a QLR study involves data collection in shorter engagements, such as serial interviews, these engagements are often referred to as data collection time points. Data collection in time waves relates to longer engagements, such as field work/observation periods. There is no clear-cut definition for the minimum time span of a QLR study; instead, the length of the data collection period must be decided based upon what processes or changes are the focus of the study [13].

Most literature describing QLR methods originates from the social sciences, where the approach has a long tradition [1, 10, 14]. In health research, one-time-data collection studies have been the norm within qualitative methods [15], although health research using QLR methods has increased in recent years [2, 5, 16, 17]. However, collecting and managing longitudinal data has its own sets of challenges, especially regarding how to integrate perspectives of time and/or change in the data collection and subsequent analysis [1]. Therefore, a study of QLR articles from the health research literature can provide an insightful understanding of the use, trends and variations of how methods are used and how elements of time/change are integrated in QLR studies. This could, in turn, provide inspiration for using different possibilities of collecting data across time when using QLR in health research. The aim of this study was to map how QLR articles within the existing health research literature are designed to capture aspects of time and/or change.

More specifically, the research questions were:

1. What methodological approaches are described to inform QLR research?
2. What methodological references are used to inform QLR research?
3. How are longitudinal perspectives articulated in article aims?
4. How is longitudinal data collection conducted?

## Methods

In this method study, we used an adapted scoping review method [18–20]. Method studies are research conducted on research studies to investigate how research design elements are applied across a field [21]. However, since there are no clear guidelines for method studies, they often use adapted versions of systematic reviews or scoping review methods [21]. The adaptations of the scoping review method consisted of 1) using a large subsample of studies (publications from a three-year period) instead of including all QLR articles published, and 2) not including grey literature. The reporting of this study was guided by the Preferred Reporting Items for Systematic reviews and Meta-Analyses extension for Scoping Reviews (PRISMA-ScR) checklist [20, 22] (see Additional file 1). A (unpublished) protocol was developed by the research team during the spring of 2019.

### Eligibility criteria

In line with method study recommendations [21], we decided to draw on a manageable subsample of published QLR research. Articles that were eligible for inclusion were health research primary studies written in English, published between 2017 and 2019, and with a longitudinal qualitative data collection. Our operating definition for qualitative longitudinal data collection was data collected at different time points (e.g., repeated interviews) or time waves (e.g., periods of field work) involving the same sample or conducted in the same setting(s). We intentionally selected a broad inclusion criterion for QLR since we wanted a wide variety of articles. The selected time period was chosen because the first QLR method article directed towards health research was published in 2013 [1] and during the following years the methodological resources for QLR increased [3, 8, 17, 23–25], thus we could expect that researchers publishing QLR in 2017–2019 should be well-grounded in QLR methods. Further, we found that from 2012 to 2019 the rate of published QLR articles were steady at around 100 publications per year, so including those from a three-year period would give a sufficient number of articles (~ 300 articles) for providing an overview of the field. Published conference abstracts, protocols, articles describing methodological issues, review articles, and non-research articles (e.g., editorials) were excluded.

### Search strategy

Relevant articles were identified through systematic searches in EBSCOhost, including biomedical and life science research and nursing and allied health literature. A librarian who specialized in systematic review searches developed and performed the searches, in collaboration with the author team (LF, TW & ÅA). In the search, the term “longitudinal” was combined with terms for qualitative research (for the search strategy see Additional file 2). The searches were conducted in the autumn of 2019 (last search 2019-09-10).

### Study selection

All identified citations were imported into EndNote X9 (www.endnote.com) and further imported into Rayyan QCRI online software [26], and duplicates were removed. All titles and abstracts were screened against the eligibility criteria by two independent reviewers (ÅA & EH), and conflicting decisions were discussed until resolved. After discussions by the team, we decided to include articles published between 2017 and 2019, that selection alone included 350 records with diverse methods and designs. The full texts of articles that were eligible for inclusion were retrieved. In the next stage, two independent reviewers reviewed each full text article to make final decisions regarding inclusion (ÅA, EH, Julia Andersson). In total, disagreements occurred in 8% of the decisions, and were resolved through discussion. Critical appraisal was not assessed since the study aimed to describe the range of how QLR is applied and not aggregate research findings [21, 22].

### Data charting and analysis

A standardized charting form was developed in Excel (Excel 2016). The charting form was reviewed by the research team and pretested in two stages. The tests were performed to increase internal consistency and reduce the risk of bias. First, four articles were reviewed by all the reviewers, and modifications were made to the form and charting instructions. In the next stage, all reviewers used the charting form on four other articles, and the convergence in ratings was 88%. Since the convergence was under 90%, charting was performed in duplicate to reduce errors in the data. At the end of the charting process, the convergence among the reviewers was 95%. The charting was examined by the first author, who revised the charting in cases of differences.

Data items that were charted included 1) the article characteristics (e.g., authors, publication year, journal, country), 2) the aim and scope (e.g., phenomenon of interest, population, contexts), 3) the stated methodology and analysis method, 4) text describing the data collection (e.g., type of data material, number of participants, time frame of data collection, total amount of data material), and 5) the qualitative methodological references used in the methods section. Extracted text describing data collection could consist of a few sentences or several sections from the articles (and sometimes figures) concerning data collection practices, rational for time periods and research engagement in the field. This was later used to analyze how the longitudinal data collection was conducted and elements of longitudinal design. To categorize the qualitative methodology approaches, a framework from Cresswell [27] was used (including the categories for grounded theory, phenomenology, ethnography, case study and narrative research). Overall, data items needed to be explicitly stated in the articles in order to be charted. For example, an article was categorized as grounded theory if it explicitly stated “in this grounded theory study” but not if it referred to the literature by Glaser and Strauss without situating itself as a grounded theory study (See Additional file 3 for the full instructions for charting).

All charting forms were compiled into a single Microsoft Excel spreadsheet (see Supplementary files for an overview of the articles). Descriptive statistics with frequencies and percentages were calculated to summarize the data. Furthermore, an iterative coding process was used to group the articles and investigate patterns of, for example, research topics, words in the aims, or data collection practices. Alternative ways of grouping and presenting the data were discussed by the research team.

## Results

### Search and selection

**Fig. 1:**
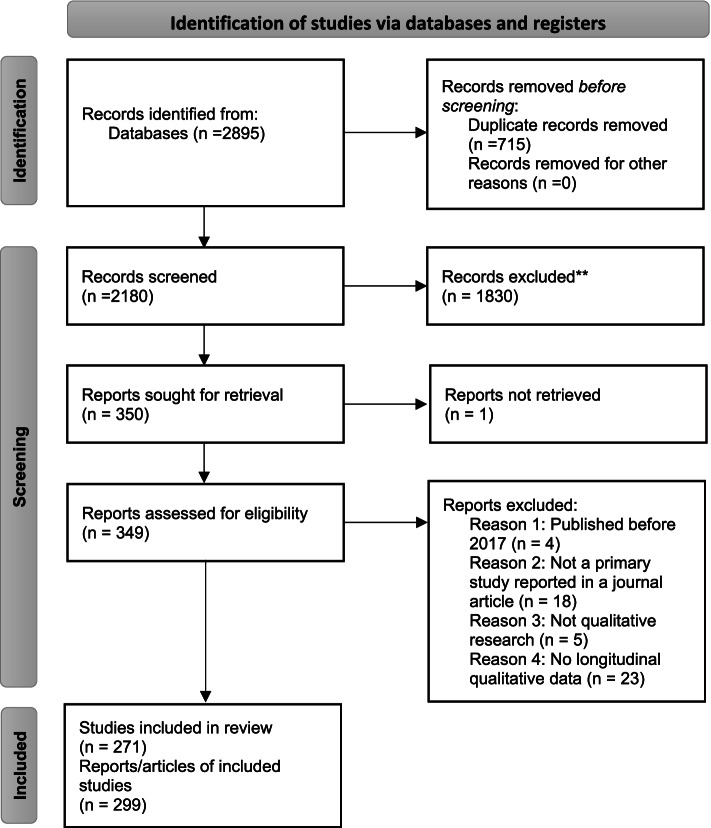
PRISMA diagram of study selection]

A total of 2179 titles and abstracts were screened against the eligibility criteria (see Fig. 1). The full text of one article could not be found and the article was excluded [28]. Fifty full text articles were excluded. Finally, 299 articles, representing 271 individual studies, were included in this study (see additional files 4 and 5 respectively for tables of excluded and included articles).

### General characteristics and research areas of the included articles

The articles were published in many journals (*n* = 193), and 138 of these journals were represented with one article each. BMJ Open was the most prevalent journal (*n* = 11), followed by the Journal of Clinical Nursing (*n* = 8). Similarly, the articles represented many countries (*n* = 41) and all the continents; however, a large part of the studies originated from the US or UK (*n* = 71, 23.7% and *n* = 70, 23.4%, respectively). The articles focused on the following types of populations: patients, families−/caregivers, health care providers, students, community members, or policy makers. Approximately 20% (*n* = 63, 21.1%) of the articles collected data from two or more of these types of population(s) (see Table 1).

**Table 1:** Characteristics of the included QLR articles

**Continents**	**n (%)**
Europe	141 (47.2)
North America	85 (28.4)
Oceania	33 (11.0)
Africa	23 (7.7)
Asia	10 (3.3)
South America	3 (1.0)
Several continents	3 (1.0)
**Population** (Articles could include several types of populations)	**n (%)**
Patients (individuals with a health condition)	122 (40.8)
Family members/caregivers	72 (24.1)
Community members (citizens, people in low income areas, volunteers)	63 (21.1)
Health care providers	61 (20.4)
Students or pupils (mostly health care education)	26 (8.7)
Policy makers	14 (4.7)
Managers	15 (5.0)
Teachers	7 (2.3)
US national news organizations	1 (0.3)
**Phenomena of interest**	**n (%)**
Disease experience/beliefs	52 (17.4)
Health care navigation and/or health care-patient relationships	48 (16.1)
Experiences with health care trials/interventions or treatment	43 (14.4)
Implementation of health care practices/routines	32 (10.7)
Life transitions and development (pregnancy, breastfeeding, parenthood, adolescence, aging)	23 (7.7)
Societal adversities (violence, housing, drug addiction, criminality)	22 (7.4)
Health care providers’ professional development	20 (6.7)
Education	18 (6.0)
Family caregiving	14 (4.7)
Health behaviors and sports (e.g., physical activity, smoking cessation, talent development)	11 (3.7)
Policy development and social reform	5 (1.7)
Experience of technology (assistive technology, aids and adaptations)	4 (1.3)
Disaster experiences (flooding, earthquakes)	3 (1.0)
**Context** (from which participants were recruited. Articles could have several contexts)	**n (%)**
**Health-care/patient associations**	**194 (64.9)**
Specialist care/Hospital	84 (28.1)
Emergency/intensive/neonatal care	15 (5.0)
Primary care	12 (4.0)
Residential homes/nursing homes	7 (2.3)
**Community**	46 (15.8)
**Schools/universities**	32 (10.7)
**Social services/community services, volunteer organizations, prison**	27 (9.0)
Rural	11 (3.7)
Urban	16 (5.4)
Socially vulnerable area	25 (8.63)
Diversity of contexts (e.g., rural and urban area)	14 (4.7)

Approximately half of the articles (*n* = 158, 52.8%) articulated being part of a larger research project. Of them, 95 described a project with both quantitative and qualitative methods. They represented either 1) a qualitative study embedded in an intervention, evaluation or implementation study (*n* = 66, 22.1%), 2) a longitudinal cohort study collecting both quantitative and qualitative material (*n* = 23, 7.7%), or 3) qualitative longitudinal material collected together with a cross sectional survey (n = 6, 2.0%). Forty-eight articles (16.1%) described belonging to a larger qualitative project presented in several research articles.

### Methodological traditions

Approximately one-third (*n* = 109, 36.5%) of the included articles self-identified with one of the qualitative traditions recognized by Cresswell [27] (case study: *n* = 36, 12.0%; phenomenology: *n* = 35, 11.7%; grounded theory: *n* = 22, 7.4%; ethnography: *n* = 13, 4.3%; narrative method: n = 3, 1.0%). In nine articles, the authors described using a mix of two or more of these qualitative traditions. In addition, 19 articles (6.4%) self-identified as mixed methods research.

Every second article self-identified as having a qualitative longitudinal design (*n* = 156, 52.2%); either they self-identified as “a longitudinal qualitative study” or “using a longitudinal qualitative research design”. However, in some articles, this was stated in the title and/or abstract and nowhere else in the article. Fifty-two articles (17.4%) self-identified both as having a QLR design and following one of the methodological approaches (case study: *n* = 8; phenomenology: *n* = 23; grounded theory: *n* = 9; ethnography: *n* = 6; narrative method: *n* = 2; mixed methods: *n* = 4).

The other 143 articles used various terms to situate themselves in relation to a longitudinal design. Twenty-seven articles described themselves as a longitudinal study (9.0%) or a longitudinal study within a specific qualitative tradition (e.g., a longitudinal grounded theory study or a longitudinal mixed method study) (*n* = 64, 21.4%). Furthermore, 36 articles (12.0%) referred to using longitudinal data materials (e.g., longitudinal data or longitudinal interviews). Nine of the articles (3.0%) used the term longitudinal in relation to the data analysis or aim (e.g., the aim was to longitudinally describe), used terms such as serial or repeated in relation to the data collection design (*n* = 2, 0.7%), or did not use any term to address the longitudinal nature of their design (*n* = 5, 1.7%).

### Use of methodological references

The mean number of qualitative method references in the methods sections was 3.7 (range 0 to 16), and 20 articles did not have any qualitative method reference in their methods sections.^1^ Commonly used method references were generic books on qualitative methods, seminal works within qualitative traditions, and references specializing in qualitative analysis methods (see Table 2). It should be noted that some references were comprehensive books and thus could include sections about QLR without being focused on the QLR method. For example, Miles et al. [31] is all about analysis and coding and includes a chapter regarding analyzing change.

**Table 2:** Most frequently used method references (8 most used) and QLR method references (5 most used). Citations in Google Scholar were used as an indication of how widely used the references are; searches conducted in Google Scholar 2022-01-02

	N (%)	Description
**Methodological reference**		
Braun & Clark [29]	43 (14.4)	Early, widespread description of thematic analysis. 117,046 citations in Google Scholar.
Patton [30]	29 (9.7)	Early, comprehensive book about conducting research using qualitative methods. References included 2nd, 3rd and 4th editions, published between 1990 and 2015. 111,407 citations in Google Scholar.
Miles, Huberman & Saldaña [31]	22 (7.4)	Comprehensive book about analysis and coding. This edition was coauthored with Saldana who has previously written about QLR. 420 citations in Google Scholar. The book is a developed version and the first edition was published in 1994 [32] (144,063 citations in Google Scholar). This latter edition was used by 14 articles in the sample.
Smith, Flowers & Larkin [33]	20 (6.7)	Comprehensive book on Interpretative Phenomenological Analysis. 605 citations in Google Scholar.
Hsieh & Shannon [34]	19 (6.4)	Widespread early overview of content analysis. 36,554 citations in Google Scholar.
Glaser & Strauss [35]	17 (5.7)	First book describing grounded theory. 150,386 citations in Google Scholar.
Tong., et al., [36]	16 (5.4)	First guidelines on the reporting of qualitative articles within health research. 14,302 citations in Google Scholar.
**QLR method references**		
Calman, Brunton & Molassiotis [1]	15 (5.0)	One of the first articles describing the QLR method from a health research perspective. 211 citations in Google Scholar.
Saldaña [13]	15 (5.0)	Methodological book with influence on the further development of QLR, mainly drawing on ethnographical traditions and examples from theatre education. 880 citations in Google Scholar.
Murray [37]	11 (3.7)	Article giving practical advice on the use of serial interviewing. 301 citations in Google Scholar.
Grossoehme & Lipstein [3]	7 (2.3)	Article about QLR analysis, giving examples and advice regarding two different analysis approaches. 147 citations in Google Scholar.
Thomson & Holland [38]	5 (1.7)	One article of several that originated from an early report on how QLR was used in UK. This article outlines several challenges and solutions when working with QLR. 424 citations in Google Scholar.

Only approximately 20% (*n* = 58) of the articles referred to the QLR method literature in their methods sections.^2^ The mean number of QLR method references (counted for articles using such sources) was 1.7 (range 1 to 6). Most articles using the QLR method literature also used other qualitative methods literature (except two articles using one QLR literature reference each [39, 40]). In total, 37 QLR method references were used, and 24 of the QLR method references were only referred to by one article each.

### Longitudinal perspectives in article aims

In total, 231 (77.3%) articles had one or several terms related to time or change in their aims, whereas 68 articles (22.7%) had none. Over one hundred different words related to time or change were identified. Longitudinally oriented terms could focus on changes across time (process, trajectory, transition, pathway or journey), patterns of how something changed (maintenance, continuity, stability, shifts), or phenomena that by nature included change (learning or implementation). Other types of terms emphasized the data collection time period (e.g., over 6 months) or a specific changing situation (e.g., during pregnancy, through the intervention period, or moving into a nursing home). The most common terms used for the longitudinal perspective were change (*n* = 63), over time (*n* = 52), process (*n* = 36), transition (*n* = 24), implementation (*n* = 14), development (*n* = 13), and longitudinal (n = 13).^3^

Furthermore, the articles varied in what ways their aims focused on time/change, e.g., the longitudinal perspectives in the aims (see Table 3). In 71 articles, the change across time was the *phenomenon of interest of the article*: for example, articles investigating the process of learning or trajectories of diseases. In contrast, 46 articles investigated change or factors impacting *change in relation to a defined outcome*: for example, articles investigating factors influencing participants continuing in a physical activity trial. The longitudinal perspective could also be embedded in an article’s *context*. In such cases, the focus of the article was on experiences that happened during a certain time frame or in a time-related context (e.g., described experiences of the patient-provider relationship during 6 months of rehabilitation).

**Table 3:** Different longitudinal perspectives in the articles’ aims and objectives

How time or change is articulated in the aim	Description	Example	Number of articles
Time/change as the **phenomenon** of interest	Focus is on how changes occurs. Articles aimed to investigate phenomena such as process, trajectories or change.	Coombs, Parker and de Vries [41] aimed “to describe how decision-making influences transitions in care when approaching the end of life.” (p. 618) Thus, the focus in the aim was how decision-making influences transitions.	n = 71, 23.7%
Time/change related to the **outcomes** of the study	Focus is on the factors, reasons or explanations of why participants reach different outcomes. Articles aimed to investigate mechanisms or factors related to an outcome often in relation to a trial or intervention.	Vaghefi et al. [42] aimed to focus on “the continued use of mHealth apps and the factors underlying this behavior”. (p. 2) In this aim, the emphasis was on whether the participant maintained their use of mHealth apps and possible explanations for their use.	*n* = 46, 15.4%
Time/change as the **context** of the study	Focus is on the subjective experiences of a phenomenon that may change across time. The change is not the preliminary interest. Articles aimed to investigate experiences over a certain time period (such as during the first year of nursing school, through the intervention period, or over 6 months).	Andersen et al. [43] aimed “to explore COPD patients’ and their family members’ experiences of both participation in care during hospitalization for an acute exacerbation in chronic obstructive pulmonary disease, and of the subsequent day-to-day care at home.” (p. 4879) Here the focus of the aim was on the experiences of participation, but in the context of hospitalization and subsequent homecomings.	*n* = 93, 31.1%
Time/change **not described** in the aims.	No terms connected to time or change in the aims.	Albrecht et al. [44] (p. 68) aimed “to examine the experiences of younger adults diagnosed with acute leukemia who are actively receiving induction chemotherapy”. Their aim did not include any words showing that data were collected across time or that time/change were the focus.	*n* = 68, 22.7%
Time/change illuminated in **several** longitudinal perspectives	Articles combining several of the longitudinal perspectives in the aims and objectives. Articles could have one objective where time/change was the phenomenon of interest and another objective where time/change was the context.	Corepal et al. [45] aimed “to explore the views and experiences of adolescents who participated in a gamified PA [physical activity] intervention based on Self-determination Theory (SDT), and the temporal changes of these views and experiences over the 1-year study period. Study objectives included: 1. To explore key aspects of a gamified PA intervention over a 1-year period using a qualitative longitudinal research (QLR) method. 2. To discuss key issues relating to the intervention, such as PA opportunities/barriers, the value of competition and types of rewards and so on. 3. To explore the key influences of PA and to determine who benefited from the intervention, how and why it worked for them. 4. To qualitatively chart changes in behaviours, opinions or views as a result of participating in the intervention.” (p2) In this example, Research question 1 use a context approach to time/change; Research question 2 contain no description of time/change; Research question 3 used an outcome perspective; and Research question 4 investigated changes in behavior as a phenomenon.	*n* = 21, 7.0%

### Types of data and length of data collection

The QLR articles were often large and complex in their data collection methods. The median number of participants was 20 (range from one to 1366, the latter being an article with open-ended questions in questionnaires [46]). Most articles used individual interviews as the data material (*n* = 167, 55.9%) or a combination of data materials (*n* = 98, 32.8%) (e.g., interviews and observations, individual interviews and focus group interviews, or interviews and questionnaires). Forty-five articles (15.1%) presented quantitative and qualitative results. The median number of interviews was 46 (range three to 507), which is large in comparison to many qualitative studies. The observation materials were also comprehensive and could include several hundred hours of observations. Documents were often used as complementary material and included official documents, newspaper articles, diaries, and/or patient records.

**Fig. 2:**
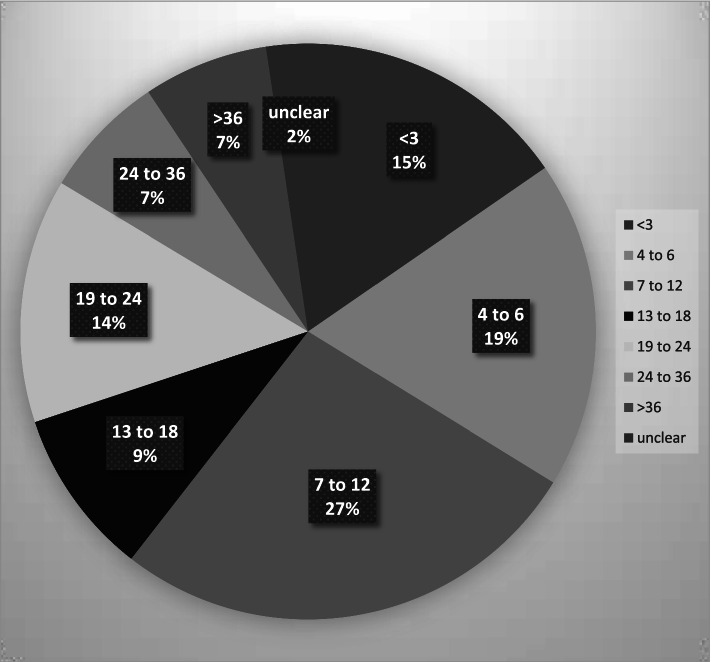
Number of articles in relation to the time span of data collection. The time span of data collection is given in months

The articles’ time spans^4^ for data collection varied between a few days and over 20 years, with 60% of the articles’ time spans being 1 year or shorter (*n* = 180) (see Fig. 2). The variation in time spans might be explained by the different kinds of phenomena that were investigated. For example, Jensen et al. [47] investigated hospital care delivery and followed each participant, with observations lasting between four and 14 days. Smithbattle [48] described the housing trajectories of teen mothers, and collected data in seven waves over 28 years.

### Three components of longitudinal data collection

In the articles, the data collection was conducted in relation to three different longitudinal data collection components (see Table 4).

**Table 4:** Components of longitudinal data collection

	Description	Example	Frequency n (%)
**1) Types of entities followed across time**			
Individual	Data are collected from the same individuals across time in an individual mode, e.g., individual interviews, questionnaires, diaries.	Albrecht et al. [44] investigated young adults’ experiences of chemotherapy treatment in the hospital. Seven young adults were interviewed twice, with interviews about one month apart. The young adults were also invited to keep a diary between the two interviews.	170 (56.9)
Individual case or dyads	Data are collected from cases based upon individuals or dyads. An individual case included a primary participant (e.g., patient) and secondary participants (e.g., family, health care providers). Dyads were based on two connected individuals being equally important (e.g., parents or spouses). Data consisted of individual and/or joint interviews, observations, and/or documents, etc.	Denney-Koelsch [49] investigated couples’ experiences meeting health care providers when pregnant, with a lethal fetal diagnosis. The couples took part in up to five interviews both individually and jointly during the pregnancy and after birth.	64 (21.4)
Groups	Data are collected from one or several defined groups (e.g., classes of students or health care teams). The groups are followed across time but members of the group can change during the data collection period. Data were often collected with the group, e.g., focus group interviews and/or observations, and complemented with individual interviews, questionnaires or documents.	Pyörälä et al. [50] followed two classes of students over a five year period of education. Data were collected with focus groups and open-ended questions in surveys. Some students took part in several data collection rounds whereas others contributed once during the years of the data collection period.	9 (3.0)
Settings (location/trial)	Data are collected at the same setting(s) across time. Settings can be locations (e.g., hospital wards, community centers) or trials (e.g., interventions). Articles often included several types of populations (e.g., patients, health care providers, family members). Over the data collection period, some participants contributed on several occasions, while some contributed once. Typical data collection methods included observations and/or recorded intervention sessions, combined with individual interviews, focus group interviews, questionnaires and/or documents.	Lindberg et al. [51] investigated how new technology was learned and used at an operational unit. Data were collected over four years through observations of training sessions, observations of daily work and medical procedures, observations of meetings and seminars, individual interviews with nurses, doctors, hospital technicians, physicists and technology suppliers, and documents. Some key participants took part in several parts of the data collection period, while others took part once. Frost et al. [52] investigated a home rehabilitation program for people with heart failure. Data consisted both of interviews at two time points with the same patients and caregivers, as well as audio recordings of the intervention sessions, and intervention fidelity scores. The timeline for the data collection followed the program with the last interview 12 months after baseline.	55 (18.4)
2) Tempo of data collection			
Baseline and follow up	Data are collected at two points in time. Can be prospectively planned or followed up with previous data material.	Young et al. [53] conducted interviews with 60 women with genetic mutations increasing the risk for breast cancer. Three years later, 12 of the women took part in a follow up interview. The current article was built on data from both interviews with these 12 women.	70 (23.4)
Serial time points	Data are collected at several shorter engagements.	Lewis et al. [54] explored women’s experiences of trust in relation to their midwives during pregnancy. Semistructured interviews were conducted at three time-points: in early pregnancy, late pregnancy and two months post-birth.	154 (51.5)
Time waves	Data are collected during time periods with some time in between the data collection periods.	Mozaffar et al. [55] explored challenges in relation to the integration of electronic prescribing systems. Semistructured interviews were complemented with observations of meetings and documents. Data were collected in two one-month periods with about two years in between the data collection periods.	50 (16.7)
Continuous data collection	Data are collected continuously for a period of time, for example, with regular observations for several days in a row, observations of all events of a certain kind or including all documents that fulfill specific criteria.	Castro et al. [56] investigated nurses work-life narratives by analyzing nurses’ blogs. The data material consisted of all blog entries by four bloggers over a one-year period, with a total of 520 entries. Jensen et al. [47, 57] studied patients with Alzheimer’s disease who were receiving hospital care after a hip fracture. The three participants were observed for several day and evening shifts during their whole hospital stay. Observations for each participant ranged from 4 to 14 days.	23 (7.7)
3) Preplanned or adapted data collection			
Preplanned data collection	The data collection is planned by the research team based upon theory, previous research and project capacity.	Nash et al. [58] investigated occupational therapy students’ changes in perspectives of frames of reference during their education. The students were interviewed at four occasions over 15 months; the interviews were scheduled at the end of each course where frames of reference were part of the curriculum.	224 (74.9)
Theoretical or analysis driven data collection	Data collection is adapted to questions raised during analysis and theoretical ideas, often using several types of data material and/or different groups of participants or stakeholders.	Bright et al., [59] investigated how health care providers engaged people with communication disabilities during rehabilitation. Data were collected in the form of observations and interviews with three patients and 28 providers. The patients were followed during the rehabilitation period for up to 12 weeks. In choosing what situations or events should be observed, the research team drew on insights from the ongoing data collection as well as previous research and theoretical notions of what situations would provide rich data.	19 (6.4)
Participant-adapted data collection	Data collection is partly preplanned but also adjusted to the individual trajectory of each participant or case to capture essential changes across time. Typically, some participants are followed more closely and for a longer period of time than other participants.	Superdock et al. [60] conducted a study about the influence of religion and spirituality on parental decision-making regarding children’s life-threatening conditions. The parents of 16 children were included as well as the children’s health care providers. The shortest individual case was followed for 6 days whereas the longest was followed for 531 days (median = 380 days). Interviews were held at the time of study enrollment and then on a monthly basis, but additional data collection was performed in the following situations: when a child had encountered a life-threatening event; when a child’s treatment had changed; when a child was discharged from the clinic; and, in some cases, a few weeks after a child’s death.	44 (14.7)
Participant entries of data	Data are independently entered by the participants. Data often consist of texts or pictures such as diary entries, think aloud methods, or answers to open-ended questions. Prompts can be sent, or participants can be encouraged to enter data in certain situations. Studies can include an entry and/or exit interview.	Gordon et al., [61] investigated experiences of the transition from trainee doctors to trained doctors. During the enrollment interview, the trainee doctors were instructed about how to provide audio diaries. Audio diaries were recorded on smartphones in order to capture thoughts and experiences in the moment. Participants received weekly reminders to provide audio diaries. In total, the audio diaries were collected over a period of 6 to 8 months and thereafter the participants took part in an exit interview.	11 (6.7)

#### Entities followed across time

Four different types of entities were followed across time: 1) individuals, 2) individual cases or dyads, 3) groups, and 4) settings. Every second article (*n* = 170, 56.9%) followed individuals across time, thus following the same participants through the whole data collection period. In contrast, when individual cases were followed across time, the data collection was centered on the primary participants (e.g., people with progressive neurological conditions) who were followed over time, and secondary participants (e.g., family caregivers) might provide complementary data at several time points or only at one-time point. When settings were followed over time, the participating individuals were sometimes the same, and sometimes changed across the data collection period. Typical settings were hospital wards, hospitals, smaller communities or intervention trials. The type of collected data corresponded with what kind of entities were followed longitudinally. Individuals were often followed with serial interviews, whereas groups were commonly followed with focus group interviews complemented with individual interviews, observations and/or questionnaires. Overall, the lengths of data collection periods seemed to be chosen based upon expected changes in the chosen entities. For example, the articles following an intervention setting were structured around the intervention timeline, collecting data before, after and sometimes during the intervention.

#### Tempo of data collection

The data collection tempo differed among the articles (e.g., the frequency and mode of the data collection). Approximately half (*n* = 154, 51.5%) of the articles used serial time points, collecting data at several reoccurring but shorter sequences (e.g., through serial interviews or open-ended questions in questionnaires). When data were collected in time waves (*n* = 50, 16.7%), the periods of data collection were longer, usually including both interviews and observations; often, time waves included observations of a setting and/or interviews at the same location over several days or weeks.

**Fig. 3:**
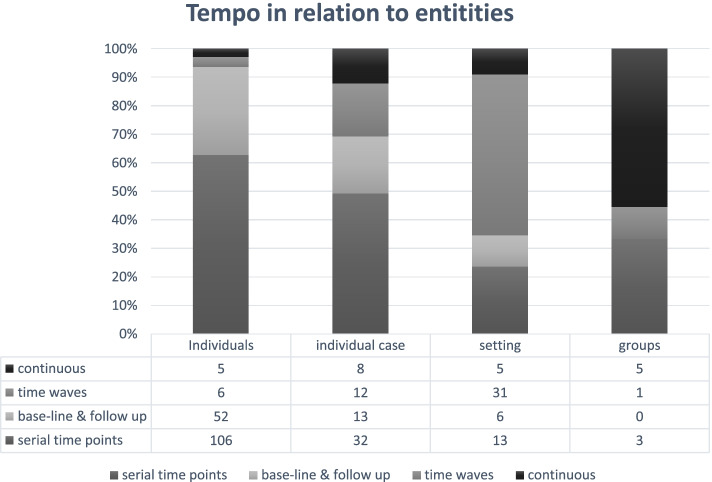
Tempo of data collection in relation to entities followed over time

When comparing the tempo with the type of entities, some patterns were detected (see Fig. 3). When individuals were followed, data were often collected at time points, mirroring the use of individual interviews and/or short observations. For research in settings, data were commonly collected in time waves (e.g., observation periods over a few weeks or months). In studies exploring settings across time, time waves were commonly used and combined several types of data, particularly from interviews and observations. Groups were the least common studied entity (*n* = 9, 3.0%), so the numbers should be interpreted with caution, but continuous data collection was used in five of the nine studies. The continuous data collection mode was, for example, collecting electronic diaries [62] or minutes from committee meetings during a time period [63].

#### Preplanned or adapted data collection

**Fig. 4:**
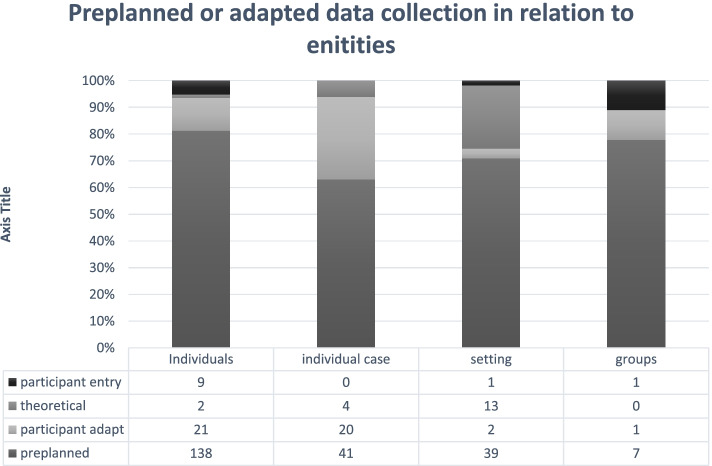
Preplanned or adapted data collection in relation to entities followed over time

A large majority (*n* = 224, 74.9%) of the articles used preplanned data collection (e.g., in preplanned data collection, all participants were followed across time according to the same data collection plan). For example, all participants were interviewed one, six and twelve months’ post-diagnosis. In contrast to the preplanned data collection approach, 44 articles had a participant-adapted data collection (14.7%), and participants were followed at different frequencies and/or over various lengths of time depending on each participant’s situation. Participant-adapted data collection was more common among articles following individuals or individual cases (see Fig. 4). To adapt the data collection to the participants, the researchers created strategies to reach participants when crucial events were happening. Eleven articles used a participant entry approach to data collection (*n* = 11, 6.7%), and the whole or parts of the data were independently sent in by participants in the form of diaries, questionnaires, or blogs. Another approach to data collection was using theoretical or analysis-driven ideas to guide the data collection (*n* = 19, 6.4%). In these articles, the analysis and data collection were conducted simultaneously, and ideas arising in the analysis could be followed up, for example, returning to some participants, recruiting participants with specific experiences, or collecting complementary types of data materials. This approach was most common in the articles following settings across time, which often included observations and interviews with different types of populations. Articles using theoretical or analysis driven data collection were not associated with grounded theory to a greater extent than the other articles in the sample (e.g., did not self-identify as grounded theory or referred to methodological literature within grounded theory traditions to a greater proportion).

## Discussion

**Fig. 5:**
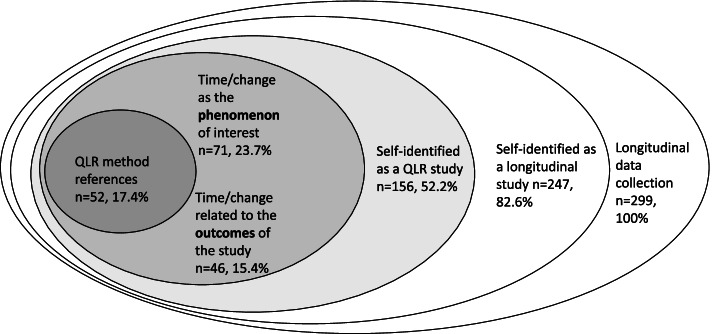
The QLR onion. The use of QLR design can be described as layered, where researchers use more or less elements of a QLR design. The two inmost layers represents articles using QLR as a methodological approach

According to our results, some researchers used QLR as a methodological approach and other researchers used a longitudinal qualitative data collection without aiming to investigate change. Adding to the debate on whether QLR is a methodological approach in its own right or a design element in a particular study we suggest that the use of QLR can be described as layered (see Fig. 5). Namely, articles must fulfill several criteria in order to use QLR as a methodological approach, and that is done in some articles. In those articles QLR method references were used, the aim was to investigate change of a phenomenon and the longitudinal elements of the data collection were thoroughly integrated into the method section. On the other hand, some articles using a longitudinal qualitative data collection were just collecting data over time, without addressing time and/or change in the aim. These articles can still be interesting research studies with valuable results, but they are not using the full potential of QLR as a methodological approach. In all, around 40% of the articles had an aim that focused on describing or understanding change (either as phenomenon or outcome); but only about 24% of the articles set out to investigate change across time as their phenomenon of interest.

Regarding methodological influences, about one-third of the articles self-identify with any of the traditional qualitative methodologies. Using a longitudinal qualitative data collection as an element integrated with another methodological tradition can therefore be seen as one way of working with longitudinal qualitative materials. In our results, the articles referring to methodologies other than QLR preferably used case study, phenomenology and grounded theory methodologies. This was surprising since Neale [10] identified ethnography, case studies and narrative methods as the main methodological influences on QLR. Our findings might mirror the profound impacts that phenomenology and grounded theory have had on the qualitative field of health research. Regarding phenomenology, the findings can also be influenced by more recent discussions of combining interpretative phenomenological analysis with QLR [6].

Half of the articles self-identified as QLR studies, but QLR method references were used in less than 20% of the identified articles. This is both surprising and troublesome since use of appropriate method literature might have supported researchers who were struggling with for example a large quantity of materials and complex analysis. A possible explanation for the lack of use of QLR method literature is that QLR as a methodological approach is not well known, and authors might not be aware that method literature exists. It is quite understandable that researchers can describe a qualitative project with longitudinal data collection as a qualitative longitudinal study, without being aware that QLR is a specific form of study. Balmer [64] described how their group conducted serial interviews with medical students over several years before they became aware of QLR as a method of study. Within our networks, we have met researchers with similar experiences. Likewise, peer reviewers and editorial boards might not be accustomed to evaluating QLR manuscripts. In our results, 138 journals published one article between 2017 and 2019, and that might not be enough for editorial boards and peer reviewers to develop knowledge to enable them to closely evaluate manuscripts with a QLR method.

In 2007, Holland and colleagues [65] mapped QLR in the UK and described the following four categories of QLR: 1) mixed methods approaches with a QLR component; 2) planned prospective longitudinal studies; 3) follow-up studies complementing a previous data collection with follow-up; and 4) evaluation studies. Examples of all these categories can be found among the articles in this method study; however, our results do paint a more complex picture. According to our results, Holland’s categories are not multi-exclusive. For example, studies with intentions to evaluate or implement practices often used a mixed methods design and were therefore eligible for both categories one and four described above. Additionally, regarding the follow-up studies, it was seldom clearly described if they were planned as a two-time-point study or if researchers had gained an opportunity to follow up on previous data collection. When we tried to categorize QLR articles according to the data collection design, we could not identify multi-exclusive categories. Instead, we identified the following three components of longitudinal data collection: 1) entities followed across time; 2) tempo; and 3) preplanned or adapted data collection approaches. However, the most common combination was preplanned studies that followed individuals longitudinally with three or more time points.

The use of QLR differs between disciplines [14]. Our results show some patterns for QLR within health research. Firstly, the QLR projects were large and complex; they often included several types of populations and various data materials, and were presented in several articles. Secondly, most studies focused upon the individual perspective, following individuals across time, and using individual interviews. Thirdly, the data collection periods varied, but 53% of the articles had a data collection period of 1 year or shorter. Finally, patients were the most prevalent population, even though topics varied greatly. Previously, two other reviews that focused on QLR in different parts of health research (e.g., nursing [4] and gerontology [66]) pointed in the same direction. For example, individual interviews or a combination of data materials were commonly used, and most studies were shorter than 1 year but a wide range existed [4, 66].

### Considerations when planning a QLR project

Based on our results, we argue that when health researchers plan a QLR study, they should reflect upon their perspective of time/change and decide what part change should play in their QLR study. If researchers decide that change should play the main role in their project, then they should aim to focus on change as the phenomenon of interest. However, in some research, change might be an important part of the plot, without having the main role, and change in relation to the outcomes might be a better perspective. In such studies, participants with change, no change or different kinds of change are compared to explore possible explanations for the change. In our results, change in relation to the outcomes was often used in relation to intervention studies where participants who reached a desired outcome were compared to individuals who did not. Furthermore, for some research studies, change is part of the context in which the research takes place. This can be the case when certain experiences happen during a period of change; for example, when the aim is to explore the experience of everyday life during rehabilitation after stroke. In such cases a longitudinal data collection could be advisable (e.g., repeated interviews often give a deep relationship between interviewer and participants as well as the possibility of gaining greater depth in interview answers during follow-up interviews [15]), but the study might not be called a QLR study since it does not focus upon change [13]. We suggest that researchers make informed decisions of what kind of longitudinal perspective they set out to investigate and are transparent with their sources of methodological inspiration.

We would argue that length of data collection period, type of entities, and data materials should be in accordance with the type of change/changing processes that a study focuses on. Individual change is important in health research, but researchers should also remember the possibility of investigating changes in families, working groups, organizations and wider communities. Using these types of entities were less common in our material and could probably grant new perspectives to many research topics within health. Similarly, using several types of data materials can complement the insights that individual interviews can give. A large majority of the articles in our results had a preplanned data collection. Participant-adapted data collection can be a way to work in alignment with a “time-as-fluid” conceptualization of time because the events of subjective importance to participants can be more in focus and participants (or other entities) change processes can differ substantially across cases. In studies with lengthy and spaced-out data collection periods and/or uncertainty in trajectories, researchers should consider participant-adapted or participant entry data collection. For example, some participants can be followed for longer periods and/or with more frequency.

Finally, researchers should consider how to best publish and disseminate their results. Many QLR projects are large, and the results are divided across several articles when they are published. In our results, 21 papers self-identified as a mixed methods project or as part of a larger mixed methods project, but most of these did not include quantitative data in the article. This raises the question of how to best divide a large research project into suitable pieces for publication. It is an evident risk that the more interesting aspects of a mixed methods project are lost when the qualitative and quantitative parts are analyzed and published separately. Similar risks occur, for example, when data have been collected from several types of populations but are then presented per population type (e.g., one article with patient data and another with caregiver data). During the work with our study, we also came across studies where data were collected longitudinally, but the results were divided into publications per time point. We do not argue that these examples are always wrong, there are situations when these practices are appropriate. However, it often appears that data have been divided without much consideration. Instead, we suggest a thematic approach to dividing projects into publications, crafting the individual publications around certain ideas or themes and thus using the data that is most suitable for the particular research question. Combining several types of data and/or several populations in an analysis across time is in fact what makes QLR an interesting approach.

### Strengths and limitations

This method study intended to paint a broad picture regarding how longitudinal qualitative methods are used within the health research field by investigating 299 published articles. Method research is an emerging field, currently with limited methodological guidelines [21], therefore we used scoping review method to support this study. In accordance with scoping review method we did not use quality assessment as a criterion for inclusion [18–20]. This can be seen as a limitation because we made conclusions based upon a set of articles with varying quality. However, we believe that learning can be achieved by looking at both good and bad examples, and innovation may appear when looking beyond established knowledge, or assessing methods from different angles. It should also be noted that the results given in percentages hold no value for what procedures that are better or more in accordance with QLR, the percentages simply state how common a particular procedure was among the articles.

As described, the included articles showed much variation in the method descriptions. As the basis for our results, we have only charted explicitly written text from the articles, which might have led to an underestimation of some results. The researchers might have had a clearer rationale than described in the reports. Issues, such as word restrictions or the journal’s scope, could also have influenced the amount of detail that was provided. Similarly, when charting how articles drew on a traditional methodology, only data from the articles that clearly stated the methodologies they used (e.g., phenomenology) were charted. In some articles, literature choices or particular research strategies could implicitly indicate that the researchers had been inspired by certain methodologies (e.g., referring to grounded theory literature and describing the use of simultaneous data collection and analysis could indicate that the researchers were influenced by grounded theory), but these were not charted as using a particular methodological tradition. We used the articles’ aims and objectives/research questions to investigate their longitudinal perspectives. However, as researchers have different writing styles, information regarding the longitudinal perspectives could have been described in surrounding text rather than in the aim, which might have led to an underestimation of the longitudinal perspectives.

The experience and diversity of the research team in our study was a strength. The nine authors on the team represent ten universities and three countries, and have extensive experience in different types of qualitative research, QLR and review methods. The different level of experiences with QLR within the team (some authors have worked with QLR in several projects and others have qualitative experience but no experience in QLR) resulted in interesting discussions that helped drive the project forward. These experiences have been useful for understanding the field.

## Conclusion

Based on a method study of 299 articles, we can conclude that QLR in health research articles published between 2017 and 2019 often contain comprehensive complex studies with a large variation in topics. Some research was thoroughly designed to capture time/change throughout the methodology, focus and data collection, while other articles included a few elements of QLR. Longitudinal data collection included several components, such as what entities were followed across time, the tempo of data collection, and to what extent the data collection was preplanned or adapted across time. In sum, health researchers need to be considerate and make informed choices when designing QLR projects. Further research should delve deeper into what kind of research questions go well with QLR and investigate the best practice examples of presenting QLR findings.

## Supplementary Information

**Additional file 1.** PRISMA-ScR checklist.

**Additional file 2.** Data base searches.

**Additional file 3.** Guidelines for data charting

**Additional file 4.** List of excluded articles

**Additional file 5.** Table of included articles (author(s), year of publication, reference, country, aims and research questions, methodology, type of data material, length of data collection period, number of participants)

**Additional file 6.** Dataset

## Data Availability

The datasets used and analyzed in this current study are available in supplementary file 6.

## Footnotes

1 Qualitative method references were defined as a journal article or book with a title that indicated an aim to guide researchers in qualitative research methods and/or research theories. Primary studies, theoretical works related to the articles’ research topics, protocols, and quantitative method literature were excluded. References written in a language other than English was also excluded since the authors could not evaluate their content.

2 QLR method references were defined as a journal article or book that 1) focused on qualitative methodological questions, 2) used terms such as ‘longitudinal’ or ‘time’ in the title so it was evident that the focus was on longitudinal qualitative research. Referring to another original QLR study was not counted as using QLR method literature.

3 Words were charted depending on their word stem, e.g., change, changes and changing were all charted as change.

4 It should be noted that here time span refers to the data collection related to each participant or case. Researchers could collect data for 2 years but follow each participant for 6 months.
