# Supplementary material for: Qualitative longitudinal research in health research: a method study
Source: BMC Med Res Methodol. 2022 Oct 1;22:255. doi: 10.1186/s12874-022-01732-4 (PMC9526289; doi:10.1186/s12874-022-01732-4)
Supplement: Supplementary file 1 — Additional file 1. PRISMA-ScR checklist. [file 12874_2022_1732_MOESM1_ESM.docx]

## **Additional file 1: PRISMA-ScR Checklist**

| **Item** | **Section** | **PRISMA-ScR Checklist Item** | **Yes/no** | **Comment** |
| --- | --- | --- | --- | --- |
| 1 | Title | Identify the report as a scoping review | no | The study is primarily a method study but inspired by a scoping review method. Scoping review method is described in the abstract. |
|  | **Abstract** |  |  |  |
| 2 | Structured summary | Provide a structured summary that includes (as applicable) background, objectives, eligibility criteria, sources of evidence, charting methods, results, and conclusions that relate to the review questions and objectives. | yes |  |
|  | **Introduction** |  |  |  |
| 3 | Rationale | Describe the rationale for the review in the context of what is already known. Explain why the review questions/objectives lend themselves to a scoping review approach. | yes | Described in the introduction of the method section. |
| 4 | Objectives | Provide an explicit statement of the questions and objectives being addressed with reference to their key elements (e.g., population or participants, concepts, and context) or other relevant key elements used to conceptualize the review questions and/or objectives. | yes |  |
|  | **Methods** |  |  |  |
| 5 | Protocol and registration | Indicate whether a review protocol exists; state if and where it can be accessed (e.g., a Web address); and if available, provide registration information, including the registration number. | Not published. | The research team developed a protocol, but it was not published. The protocol can be received if requested. |
| 6 | Eligibility criteria | Specify characteristics of the sources of evidence used as eligibility criteria (e.g., years considered, language, and publication status), and provide a rationale. | yes |  |
| 7 | Information sources | Describe all information sources in the search (e.g., databases with dates of coverage and contact with authors to identify additional sources), as well as the date the most recent search was executed. | yes |  |
| 8 | Search | Present the full electronic search strategy for at least 1 database, including any limits used, such that it could be repeated. | yes | Can be found in supplementary file 2: Data base searches. |
| 9 | Selection of sources of evidence | State the process for selecting sources of evidence (i.e., screening and eligibility) included in the scoping review. | yes |  |
| 10 | Data charting process | Describe the methods of charting data from the included sources of evidence (e.g., calibrated forms or forms that have been tested by the team before their use, and whether data charting was done independently or in duplicate) and any processes for obtaining and confirming data from investigators. | yes |  |
| 11 | Data items | List and define all variables for which data were sought and any assumptions and simplifications made. | yes | The full instructions for the data charting is available in a supplementary file 3: Guidelines for data charting |
| 12 | Critical appraisal of individual sources of evidence§ | If done, provide a rationale for conducting a critical appraisal of included sources of evidence; describe the methods used and how this information was used in any data synthesis (if appropriate). | n/a |  |
| 13 | Summary measures | Not applicable for scoping reviews. | n/a |  |
| 14 | Synthesis of results | Describe the methods of handling and summarizing the data that were charted. | yes |  |
| 15 | Risk of bias across studies | Not applicable for scoping reviews. | n/a |  |
| 16 | Additional analyses | Not applicable for scoping reviews. | n/a |  |
|  | **Results** |  |  |  |
| 17 | Selection of sources of evidence | Give numbers of sources of evidence screened, assessed for eligibility, and included in the review, with reasons for exclusions at each stage, ideally using a flow diagram. | yes | A PRISMA diagram is included in the article. |
| 18 | Characteristics of sources of evidence | For each source of evidence, present characteristics for which data were charted and provide the citations. | yes | 299 articles were included; a table is provided in supplementary file 5: List of included articles. |
| 19 | Critical appraisal within sources of evidence | If done, present data on critical appraisal of included sources of evidence (see item 12). | n/a |  |
| 20 | Results of individual sources of evidence | For each included source of evidence, present the relevant data that were charted that relate to the review questions and objectives. | yes | The article table (supplementary file 5) includes author(s), year of publication, reference, country, aims and research questions, methodology, type of data material, length of data collection period, number of participants. The full data set can be accessed on request. |
| 21 | Synthesis of results | Summarize and/or present the charting results as they relate to the review questions and objectives. | yes |  |
| 22 | Risk of bias across studies | Not applicable for scoping reviews. | n/a |  |
| 23 | Additional analyses | Not applicable for scoping reviews. | n/a |  |
|  | **Discussion** |  |  |  |
| 24 | Summary of evidence | Summarize the main results (including an overview of concepts, themes, and types of evidence available), link to the review questions and objectives, and consider the relevance to key groups. | yes |  |
| 25 | Limitations | Discuss the limitations of the scoping review process. | yes |  |
| 26 | Conclusions | Provide a general interpretation of the results with respect to the review questions and objectives, as well as potential implications and/or next steps. | yes |  |
| 27 | Funding | Describe sources of funding for the included sources of evidence, as well as sources of funding for the scoping review. Describe the role of the funders of the scoping review | yes | No specific funding has been received for this study. |
