# Supplementary material for: Qualitative longitudinal research in health research: a method study
Source: BMC Med Res Methodol. 2022 Oct 1;22:255. doi: 10.1186/s12874-022-01732-4 (PMC9526289; doi:10.1186/s12874-022-01732-4)
Supplement: Supplementary file 2 — Additional file 2. Data base searches. [file 12874_2022_1732_MOESM2_ESM.docx]

## **Additional file 2: Data base searches.**

Search strategy in EBESCOhost. The last searches were conducted 2019-09-10

| **#** | **Query** | **Limiters/Expanders** | **Results** |
| --- | --- | --- | --- |
| S1 | Longitudinal |  | 357,010 |
| S2 | (MH "Qualitative Research+") OR (MH "Qualitative Studies+") OR phenomenological* OR (qualitative W1 (research* OR design* OR stud* OR method*)) |  | 250,653 |
| S3 | S1 AND S2 |  | 4,634 |
| S4 | S3 | Limiters - Published Date: 20120101-20201231 Search modes - Boolean/Phrase | 2,894 |
