# Supplementary material for: Qualitative longitudinal research in health research: a method study
Source: BMC Med Res Methodol. 2022 Oct 1;22:255. doi: 10.1186/s12874-022-01732-4 (PMC9526289; doi:10.1186/s12874-022-01732-4)
Supplement: Supplementary file 3 — Additional file 3. Guidelines for data charting [file 12874_2022_1732_MOESM3_ESM.docx]

# **Additional file 3: Guidelines for data charting**

## First:

- If you are unsure about what information you should chart in a single cell. Change the text colour to red and write your concerns in a parenthesis.
- If you judge a whole article as complicated and difficult to read change the text colour of the authors names (column B) to red.
- Where it is possible, suitable text should be cut from the article and pasted into the exel sheet. If you need to condense text, make it **bold**.
- Each article must be charted in one row.

## General article characteristics

- All articles in your list will have an article number, author, publication year, title and journal already filled. If anything looks strange here (for example misspelled author name), highlight the problem. Otherwise do not change anything in these columns. Your name is put next to the article number so we can easily back trace who has charted the data.
- **Country (column F):** Enter in which country or countries **the data is collected**. Use English names on countries. USA is written US, Great Britain is written UK. If data is collected in several countries name all.
- **Text described for longitudinal method/longitudinal design (column G):** Is it stated in the article that this study is a 'longitudinal qualitative study'? This is most often written out in the abstract, title, rational or the introduction section to the methods. Extract that text. For example; “In this qualitative longitudinal study…” “We used qualitative longitudinal method…”, “The study had a longitudinal qualitative approach…” “This longitudinal etnographic study…” “This was a longitudinal study…” “In this logitudinal mixed methods study…” “We collected longitudinal interviews…” “This study used recurrent data collection…”
- **Aim and research questions (column H):** Chart the **aim and the research questions/objectives**. Use the aim in the article, most often found in the end of the background section. If the article lacks a clear aim, extract the aim from the abstract and write abstract in a paranthesis.

## PICO- Population

- **Which type of population(s):** What population is in focus of this article? Cut and paste the terms used in the aim or text close to the aim. For example, "nursing students", "patients with heart failure", "care-givers". We do **not** need to extract all inclusion criteria.
- **Phenomena of interest**: Cut and paste the 'phenomena of interest' that is **stated in the aim or objectives.** What is the focus/phenomena of the article. For example, it could be ‘learning’, ‘experiences of an education program’, ‘experience of being admitted to hospital’, ‘attitudes towards HIV’.
- **Longitudinal aspect in the aim and or research questions (related to phenomena of interest):** Is there any longitudinal oriented terms in the aim or objectives? Longitudinal oriented terms are words that indicate that the research was focused on time or change. For exempel: 'over time change of', time, changing, development, process, increase, decrease, over three months, learning. Cut and paste the longitudinal oriented terms used in the aims or objectives.
- **Context: Setting, location, or environment:** Cut and paste a short description (one or two sentences) of the environment were the study took place. Context in this column can be a ward, hospital, city, village, etc. This is often described in the beginning of the method, sometimes in the data collection or under a context heading.
- **Context: Part of larger research project:** This column is for sorting reasons. If the article is described as part of a larger research project, chose yes/no in the list. This is often described in the beginning of the method section or in the section just before the aim.
- **Context: Description of larger research project:** Cut and paste a short description of the larger project (one or two sentences). Most important is to capture the larger projects overall design (for example trial, longitudinal cohort study) if stated.

## Stated methodology

- **Type of methodology:** This column is for sorting reasons. Choose from list (grounded theory, ethnography, phenomenology, narrative research, case study or other) (Creswell & Poth, 2018, s8-11) These types of methodologies should be named in the article. We do not evaluate if the authors have applied the methodology correctly but just choose the stated methodology. Our groupings are broad so if the author states constructive grounded theory we categorize it as grounded theory. If the authors name multiple case study we chose case study.
- **If other, how does the authors define methodology**: If the author does not name any of the above methodologies (or names several or the text is complicated) cut and paste the section were the author describe methodology (or ontology/epistemology). Example: mixed methods, critical theory. Some authors do just say “qualitative method” and others will say “qualitative longitudinal design”. Methodologies are not exclusive to each other, an article might use several methodological labels.
- **Qualitative method references**: Cut and paste from reference list the full references informing qualitative method used in the method section (including design, participants, data collection and analysis). References that should NOT be extracted: references on quantitative method, data programs, protocols, empirical studies (for example informing interview guides).

## Data collection method

- **One or several types of data materials:** This column is for sorting reasons. Note in the list if the article uses one or several types of data material. For example, individual interviews and diaries, or focus group interviews and individual interviews.
- **Type of qualitative data material:** Chose from list (individual interviews, focus group interviews, blog texts, diaries, observations, documents, photos, other). If neither applies or if the article uses several choose 'other'. Alternatives:
- **Specify qualitative data material:** Cut and paste one or a few sentences about how the authors describe the qualitative data material used in the article.
- **Is there complimentary quantitative data:** Choose Yes/No. In the case of yes the article must collect, and analyze quantitative data such as validated instruments, or physiological tests. **Solely demographic data do not count.** Neither does quantitative data collected but not used in the article.
- **Methodological literature references:** Extract all qualitative methodological references used in the method section. Other original studies do not count, nor do protocol, quantitative methodological literature or references in other language than English.

## Time frame for data collection

- **How long data collection time (in months)**: Specify for how long time the participants or settings were followed. For example: a study can collect data for 12 months but each participant is interviewed twice with six months in between. In this case extract six months, because that is the time that passed between the two interviews with each participant. Another example: A hospital ward was followed with repeated observations over five months, then five months is extracted. Sometimes articles give an average and sometimes this isn’t written clearly. Copy and past sentences or section if relevant.
- **Number of time points or data collection waves**: Time points are the repeated data collection points used in the article. For example, if participants been interviewed at four occasions over two years, the article will have four time points. Time waves are when researchers have followed the participants or setting closer for a period. For example, a researcher might do observations at a hospital unit several times during two weeks (time wave 1), and again five months later for more observations and interviews during two weeks (time wave 2). Sometimes time points or time waves are not clearly described, then write ”unclear”.
- **Specify how article describe data collection practices over time**: Cut and paste the overall description for how the data collection have been conducted over time. One or a few sentences.

## Total amount of data material

- **Number of participants (1st time point)**: Cut and paste the total number of participants at the first data collection point or the total number of people participating in the article. If this is unclear or the article does not collect data from individual participants’ write “unclear”.
- **Total amount of data material**: Total number of interviews or other data documents (for example total number of observations, blogg extracts etc). If the article state that a total number of interviews were 89 that is perfect. Another possibility is to chart “49 interviews at time point 1 and 40 interviews at time point 2”. If it is not clear how much data was collected and used in the article write ’unclear’. Remember to specify if your number stands for number of interviews or pages of text, etc.

## Analysis method

- **Stated analysis method** cut and paste if authors name the data analysis method (e.g., content analysis, thematic analysis). Often described in the beginning of the analysis section.

## Why do you like/dislike this article?

- **Why do you like/dislike this article?**: This column is optional. If you want to highlight a good example (or a bad example) that might be used in the manuscript. Motivate with one sentence your impression of the article. This column is going to be used as an audit-trail for our charting experience. This column will not be part of the formal analysis.
