# Supplementary material for: Qualitative longitudinal research in health research: a method study
Source: BMC Med Res Methodol. 2022 Oct 1;22:255. doi: 10.1186/s12874-022-01732-4 (PMC9526289; doi:10.1186/s12874-022-01732-4)
Supplement: Supplementary file 4 — Additional file 4. List of excluded articles [file 12874_2022_1732_MOESM4_ESM.docx]

## **Additional file 4: List of excluded articles**

| Reference | Reason for exclusion |
| --- | --- |
| Andrighetti, H. J., Semaka, A., & Austin, J. C. (2017). Women's experiences of participating in a prospective, longitudinal postpartum depression study: insights for perinatal mental health researchers. Archives of Women's Mental Health, 20(4), 547-559. | No longitudinal qualitative data |
| Bertuit, J., Van Lint, C. E., Rooze, M., & Feipel, V. (2018). Pregnancy and pelvic girdle pain: Analysis of pelvic belt on pain. Journal of Clinical Nursing, 27(1), e129-e137. | No qualitative research method |
| Brom, L., De Snoo-Trimp, J. C., Onwuteaka-Philipsen, B. D., Widdershoven, G. A. M., Stiggelbout, A. M., & Pasman, H. R. W. (2015). Challenges in shared decision making in advanced cancer care: A qualitative longitudinal observational and interview study. Health Expectations, 20(1), 69-84. | Published before 2017 |
| Cameron, A., Johnson, E., Atkinson, T., Evans, S., Darton, R., Porteus, J., Smith, R. & Lloyd, L. (2019). The challenges of integrating housing and care for older people. International Journal of Integrated Care (IJIC), 19, 1-2. | Not a primary study published in article format |
| Collins, T. (2017). Conducting longitudinal research with older widows: Exploring personal communities through multiple methods. Journal of Women & Aging, 29(2), 102-114. | Not a primary study published in article format |
| Cummins, S., Clark, C., Lewis, D., Smith, N., Thompson, C., Smuk, M., Stansfeld, S., Taylor, S., Fahy, A., Greenhalgh, T. & Eldridge, S. (2018). The effects of the London 2012 Olympics and related urban regeneration on physical and mental health: the ORiEL mixed-methods evaluation of a natural experiment. Public Health Research, 6(12). | Not a primary study published in article format |
| Ehrlich, C., Chester, P., Kendall, E., & Crompton, D. (2017). How do health professionals work in a recovery-oriented way? International Journal of Integrated Care, 17(3), 1-8. | Not a primary study published in article format |
| Fast, D. (2017). Dream homes and dead ends in the city: A photo essay experiment. Sociology of Health & Illness, 39(7), 1134-1148. | Not a primary study published in article format |
| Fredriksen Goldsen, K., Kim, H.-J., Jung, H. & Goldsen, J. (2019). The evolution of aging with pride—National health, aging, and sexuality/gender study: Illuminating the Iridescent life course of LGBTQ adults aged 80 years and older in the United States. International Journal of Aging & Human Development, 88(4), 380-404. | No longitudinal qualitative data |
| Golden, S., Thomas, Jr., C. R., & Slatore, C., (2017). It wasn't as bad as I thought it would be: A prospective, qualitative longitudinal study of early stage non-small cell lung cancer patients after treatment. International Journal of Radiation Oncology, Biology, Physics, 98(1), 241-242. | Not a primary study published in article format |
| Goldenberg, T., Darbes, L. A., & Stephenson, R. (2018). Inter-partner and temporal variations in the perception of sexual risk for HIV. AIDS & Behavior, 22(6), 1870-1884. | No longitudinal qualitative data |
| Hays, R., Daker-White, G., Esmail, A., Barlow, W., Minor, B., Brown, B., Blakeman, T., Sanders, C. & Bower, P. (2017). Threats to patient safety in primary care reported by older people with multimorbidity: Baseline findings from a longitudinal qualitative study and implications for intervention. BMC Health Services Research, 17(1), 754-754. | No longitudinal qualitative data |
| Hunter, R., Noble, S., Lewis, S., & Bennett, P. (2019). Long-term psychosocial impact of venous thromboembolism: A qualitative study in the community. BMJ Open, 9(2), e024805-e024805. | No longitudinal qualitative data |
| Hutt Greenyer, C. (2017). The lived experience of engagement in occupations by older people during the first year of widowhood. Doctoral thesis. University of Southampton. | Not a primary study published in article format |
| Jardine, E. E., McLellan, J., & Dombrowski, S. U. (2016). Is being resolute better than being pragmatic when it comes to breastfeeding? Longitudinal qualitative study investigating experiences of women intending to breastfeed using the Theoretical Domains Framework. Journal Of Public Health, 39(3), e88-e94. | Published before 2017 |
| Juliff, D., Russell, K., & Bulsara, C. (2017). The essence of helping: Significant others and nurses in action draw men into nursing. Contemporary Nurse, 53(2), 156-166. | No longitudinal qualitative data |
| Kang, M., Robards, F., Sanci, L., Steinbeck, K., Jan, S., Hawke, C., Kong, M. & Usherwood, T. (2017). Access 3 project protocol: Young people and health system navigation in the digital age: A multifaceted, mixed methods study. BMJ Open, 7(8), e017047-e017047. | Not a primary study published in article format |
| Kean, S., Salisbury, L. G., Rattray, J., Walsh, T. S., Huby, G., & Ramsay, P. (2016). 'Intensive care unit survivorship' - A constructivist grounded theory of surviving critical illness. Journal of Clinical Nursing, 26(19), 3111-3124. | Published before 2017 |
| Khisa, W., Wakasiaka, S., McGowan, L., Campbell, M., & Lavender, T. (2016). Understanding the lived experience of women before and after fistula repair: A qualitative study in Kenya. An International Journal of Obstetrics and Gynaecology, 124(3), 503-510. | Published before 2017 |
| Klever, P. (2018). Goal effectiveness and family functioning. American Journal of Family Therapy, 46(2), 195-212. | No longitudinal qualitative data |
| Klinga, C., Hasson, H., Sachs, M. A., & Hansson, J. (2018). Dynamics of sustainable integration of health and social care: A 20-year case study. International Journal of Integrated Care, 18(S2), A218. | No qualitative research method |
| Lemelson, R., & Tucker, A. (2017). The bird dancer and the warrior king: Divergent lived experiences of Tourette syndrome in Bali. Transcultural Psychiatry, 54(4), 540-561. | No longitudinal qualitative data |
| Lewis, M. (2019). Trust me I am a midwife. International Journal of Integrated Care, 19(S1), A307. | Not a primary study published in article format |
| Lohani, M., Hendershot, K. A., Pelletier, W., Stegenga, K., Dixon, M., Hinds, P. S., Alderfer, M. A. & Pentz, R. D. (2018). Potential benefits to families, children, and adolescents enrolled in longitudinal qualitative research. Ethics & Human Research, 40(4), 1-7. | No longitudinal qualitative data |
| Lustigman, L., & Clark, E. V. (2019). Exposure and feedback in language acquisition: Adult construals of children's early verb-form use in Hebrew. Journal of Child Language, 46(2), 241-264. | No qualitative research method |
| McKenna, V. B., Sixsmith, J., & Barry, M. M. (2017). The relevance of context in understanding health literacy skills: Findings from a qualitative study. Health Expectations: An International Journal Of Public Participation In Health Care And Health Policy, 20(5), 1049-1060. | No longitudinal qualitative data |
| McKenna, V. B., Sixsmith, J., & Barry, M. M. (2018). A qualitative study of the development of health literacy capacities of participants attending a community-based cardiovascular health programme. International Journal of Environmental Research and Public Health, 15(6), 1157. | No longitudinal qualitative data |
| McPherson, K., Fadyl, J., Theadom, A., Channon, A., Levack, W., Starkey, N., Wilkinson-Meyers, L., Kayes, N., & on behalf of the TBI Experiences Research Group (2018). Living life after traumatic brain injury: Phase 1 of a longitudinal qualitative study. The Journal of Head Trauma Rehabilitation, 33(1), E44-E52. | No longitudinal qualitative data |
| Mupambireyi, Z., & Bernays, S. (2019). Reflections on the use of audio diaries to access young people's lived experiences of HIV in Zimbabwe. Qualitative Health Research, 29(5), 680-692. | Not a primary study published in article format |
| Müller, F., Tuinman, M. A., Janse, M., Almansa, J., Sprangers, M. A. G., Smink, A., Ranchor, A. V., Fleer, J. & Hagedoorn, M. (2017). Clinically distinct trajectories of fatigue and their longitudinal relationship with the disturbance of personal goals following a cancer diagnosis. British Journal of Health Psychology, 22(3), 627-643. | No qualitative research method |
| Namukwaya, S., Paparini, S., Seeley, J., & Bernays, S. (2017). "How do we start? And how will they react?" Disclosing to young people with perinatally acquired HIV in Uganda. Frontiers In Public Health, 5, 343-343. | No longitudinal qualitative data |
| Naumann, L., Babitsch, B., & Hübner, U. (2018). Experts' Insights into eHealth-Legislation: Comparing Switzerland and Germany. Studies in Health Technology & Informatics, 253, 148-152. | No longitudinal qualitative data |
| Northcutt Bohmert, M., Hoskins, K. M., & Morash, M. (2019). Tracking methods and retention for a longitudinal sample of alcohol- and drug-involved women on probation and parole. Journal of Community Psychology, 47(5), 1064-1077. | No longitudinal qualitative data |
| Pelts, M. D., & Galambos, C. (2017). Intergroup contact: Using storytelling to increase awareness of lesbian and gay older adults in long-term care settings. Journal of Gerontological Social Work, 60(6), 587-604. | No longitudinal qualitative data |
| Pender Norton, V. (2017). The CALM Project: Teaching Mindfulness Meditation in Prim ary Care Using Computer-Based Application. CALM Project: Teaching Mindfulness Meditation In Primary Care Using Computer-Based Application, 1-1. | Not a primary study published in article format |
| Price, S. L., McGillis Hall, L., Murphy, G. T., & Pierce, B. (2018). Evolving career choice narratives of new graduate nurses. Nurse Education In Practice, 28, 86-91. | No longitudinal qualitative data |
| Ratcliff, C., Naik, A. D., Martin, L. A., & Moye, J. (2018). Examining cancer survivorship trajectories: Exploring the intersection between qualitative illness narratives and quantitative screening instruments. Palliative & Supportive Care, 16(6), 712-718. | No qualitative research method |
| Redman, R. M., Reinsvold, M. C., Reddy, A., Bennett, P. E., Hoerauf, J. M., Puls, K. M.,Ovrutsky A. R., Ly, A. R., White, G., McNeil, O. & Meredith, J. J. (2017). A successful model for longitudinal community-engaged health research: The 2040 Partners for Health Student Program. Family Practice, 34(3), 341-346. | Not a primary study published in article format |
| Robards, B., & Lincoln, S. (2017). Uncovering longitudinal life narratives: Scrolling back on Facebook. Qualitative Research, 17(6), 715-730. | Not a primary study published in article format |
| Shawyer, F., Enticott, J. C., Brophy, L., Bruxner, A., Fossey, E., Inder, B., Julian, J., Kakuma, R., Weller, P., Wilson-Evered, E., Edan, V., Slade, M. & Meadows, G. N. (2017). The PULSAR Specialist Care protocol: a stepped-wedge cluster randomized control trial of a training intervention for community mental health teams in recovery-oriented practice. BMC Psychiatry, 17(1), 172-172. | Not a primary study published in article format |
| SmithBattle, L. (2018). The past is prologue? The long arc of childhood trauma in a multigenerational study of teen mothering. Social Science & Medicine, 216, 1-9. | No longitudinal qualitative data |
| Sylvest, R., Koert, E., Vittrup, I., Birch Petersen, K., Nyboe Andersen, A., Pinborg, A., & Schmidt, L. (2018). Status one year after fertility assessment and counselling in women of reproductive age-a qualitative study. Upsala Journal Of Medical Sciences, 123(4), 264-270. | No longitudinal qualitative data |
| Tam, M. (2018). Retirement and learning: A longitudinal qualitative approach. Educational Gerontology, 44(1), 54-63. | Not a primary study published in article format |
| Van der Elst, K., Bremander, A., De Groef, A., Larsson, I., Mathijssen, E. G. E., Vriezekolk, J. E., Westhovens, R. & van Eijk-Hustings, Y. J. L. (2019). European qualitative research project on patient-preferred outcomes in Early Rheumatoid Arthritis (EQPERA): Rationale, design and methods of a multinational, multicentre, multilingual, longitudinal qualitative study. BMJ Open, 9(3), e023606-e023606. | Not a primary study published in article format |
| Vanobberghen, R., Louckx, F., Devroey, D., & Vandevoorde, J. (first published 2019). Five years later: The impact of a hunger strike on undocumented migrant workers in Brussels. Journal of Immigrant and Minority Health, (2020) 22, 392–398. | No longitudinal qualitative data |
| Villalobos, M., Coulibaly, K., Krug, K., Kamradt, M., Wensing, M., Siegle, A., Kuon, J., Eschbach, C., Tessmer, G., Winkler, E., Szecsenyi, J., Ose, D. & Thomas, M. (2018). A longitudinal communication approach in advanced lung cancer: A qualitative study of patients', relatives' and staff's perspectives. European Journal Of Cancer Care, 27(2), e12794-e12794. | No longitudinal qualitative data |
| Willard, V. W., Qaddoumi, I., Zhang, H., Huang, L., Russell, K. M., Brennan, R., Wilson, M. W., Rodriguez-Galindo, C. & Phipps, S. (2017). A longitudinal investigation of parenting stress in caregivers of children with retinoblastoma. Pediatric Blood & Cancer, 64(4). | No qualitative research method |
| Williams, T. L. (2018). Exploring narratives of physical activity and disability over time: A novel integrated qualitative methods approach. Psychology of Sport & Exercise, 37, 224-234. | Not a primary study published in article format |
| Windle, G., Joling, K. J., Howson-Griffiths, T., Woods, B., Jones, C. H., van de Ven, P. M., Newman, A. & Parkinson, C. (2018). The impact of a visual arts program on quality of life, communication, and well-being of people living with dementia: a mixed-methods longitudinal investigation. International Psychogeriatrics, 30(3), 409-423. | No longitudinal qualitative data |
| Yielder, J., Wearn, A., Chen, Y., Henning, M. A., Weller, J., Lillis, S., Mogol, V. & Bagg, W. (2017). A qualitative exploration of student perceptions of the impact of progress tests on learning and emotional wellbeing. BMC Medical Education, 17(1), 148-148. | No longitudinal qualitative data |
