# Supplementary material for: Qualitative longitudinal research in health research: a method study
Source: BMC Med Res Methodol. 2022 Oct 1;22:255. doi: 10.1186/s12874-022-01732-4 (PMC9526289; doi:10.1186/s12874-022-01732-4)
Supplement: Supplementary file 5 — Additional file 5. Table of included articles (author(s), year of publication, reference, country, aims and research questions, methodology, type of data material, length of data collection period, number of participants) [file 12874_2022_1732_MOESM5_ESM.docx]

## **Additional file 5: Table of included articles**

**Reference**: Full reference of the articles.

**Country**: The country/countries were data were collected.

**Aim and research questions**: Article aim and research questions extracted from the articles background, in cases were no succinct aim could be found in the background aim is extracted from the abstract. Abbreviations are clarified.

**Type(s) of population**: Populations were sorted in the following categories: 1) patients meaning people with a medical condition and/or diagnose, 2) family members meaning people primary recruited because they were relatives to a certain group, 3) health care providers meaning people working in the health care sector, 4) people in the community meaning persons belonging to a certain community or experience a specific situation but not health care or school related, 5) students people studying in university or in lower grades (often in health-care professions), 6) teachers meaning people teaching in universities or schools, 7) managers meaning people managing a unit or organization (often a hospital ward, health-care clinic or school), 8) policy-makers meaning people in the position of making larger decisions in an organization and/or experts working with policy documents. In brackets are the articles population described in more detail.

**Type of qualitative methodology/tradition**: Self-identified methodology of the individual articles, most often extracted from the introduction of the method section. Specific methodological branches have been categorized in larger groupings, such as Interpretive Phenomenological Analysis has been categorized as phenomenology and Constructive Grounded Theory as Grounded Theory. The methodological tradition should be explicitly described in order to be extracted. For example, it should write “in this qualitative longitudinal study” in order to be categorized as belonging to a qualitative longitudinal tradition. If an article described several methodological traditions that is accounted for.

**Type of qualitative data material**: The type of qualitative data material used in the article, for example individual interviews or observations. In some cases, it was unclear to what extent different kind of data were used in the analysis, and if questionnaires included qualitative data and/or were analyzed as part of a qualitative analysis.

**Length of data collection**: The full time between the first data collection and last data collection with the same participants, cases or setting. Were possible we have extracted the wording from the articles, for example 30 days, 4 weeks or one month. In articles providing information in dates (e.g., from December 2015 until July 2017) we have made an estimate.

**Number of participants**: Total number of individuals participating in the study. Meaning that some participants have contributed with data once and some over several occasions.

**Total amount of data material**: In some articles the total amount of data were explicitly described, in other articles we have estimated the data material based on number of interviews/observations/documents at different time-points.

| Reference | Country | Aim and research questions | Type(s) of population | Type of qualitative methodology  /tradition | Type of qualitative data material | Length of data collection | Number of partici-pants | Total amount of data material |
| --- | --- | --- | --- | --- | --- | --- | --- | --- |
| Abel, G., & Thompson, L. (2018). 'I don't want to look like an AIDS victim': A New Zealand case study of facial lipoatrophy. Health & Social Care in the Community, 26(1), 41-47. | New Zealand | "This paper takes a longitudinal qualitative approach to explore one man’s struggle with the visible signs of HIV and the emotional toll this had on his life. It argues for the need for HIV treatment to look more broadly than just the reduction of viral load, and consider the physical, mental and social aspects essential for health and well- being. This paper presents a year of Tom’s emotional struggle with lipoatrophy." | Patients (persons with HIV and facial lipoatrophy) | Case study & qualitative longitudinal research | Interviews and pictures | 12 months | 1 | 4 interviews |
| Ahlstrom, L., Dellve, L., Hagberg, M., & Ahlberg, K. (2017). Women with neck pain on long-term sick leave-approaches used in the return to work process: A qualitative study. Journal of Occupational Rehabilitation, 27(1), 92-105. | Sweden | "The aim of this study was to identify approaches used in the RTW [Return to Work] process among women with neck pain on long-term sick leave from human service organizations." | Patients (women with neck pain on long-term sick leave) | Grounded theory | Individual interviews and open ended questions in questionnairs | 6 years | 117 | 496 text extractions from questionnairs, 16 interviews |
| Albrecht, T. A., Keim-Malpass, J., Boyiadzis, M., & Rosenzweig, M. (2019). Psychosocial experiences of young adults diagnosed with acute leukemia during hospitalization for induction chemotherapy treatment. Journal of Hospice & Palliative Nursing, 21(2), 167-173. | US | "...to examine the experiences of younger adults diagnosed with Acute leucemia who are actively receiving induction chemotherapy, to better direct the care delivered by oncology and palliative care clinicians." | Patients (younger adults diagnosed with Acute leucemia) | mixed methods & qualitative longitudinal research | Individual interviews and diaries | 4 to 6 weeks | 7 | 14 interviews, 2 journals |
| Allan, E. (2017). Community nursing middle management: 'dealing with different people in different time zones on both sides'. British Journal of Community Nursing, 22(9), 448-457. | UK | "What are CNMMs’ (Community nurse middle managers) perceptions of their role within CHPs (Community Health Partnerships)? What are CNMMs’ experiences and views of negotiating and managing change within CHPs? How do CNMMs understand the impact on themselves and others? What sense do they make of this? What does this mean in the context of wider understandings from the literature in Scotland and the UK? What implications are there for community nursing policy, practice, education and research?" | Health care providers (community nurse middle managers) | Phenomenology | individual interviews | 2 years | 26 | 35 interviews |
| Alonso, W. W., Kitko, L. A., & Hupcey, J. E. (2018). Intergenerational caregivers of parents with end-stage heart failure. Research & Theory for Nursing Practice, 32(4), 413-435. | US | "This study was undertaken to longitudinally examine the experiences of intergenerational young adult, adult, and older adult caregivers as they cared for a parent with end-stage HF [heart failure] in the context of the caregiver empowerment model (Jones, Winslow, Lee, Burns, & Zhang, 2011)." | Family members (intergenerational caregivers) | Grounded theory | Individual interviews | up to 24 months | 23 | 114 interviews |
| Alvarez, S., & Schultz, J.-H. (2019). Professional and personal competency development in near-peer tutors of gross anatomy: A longitudinal mixed-methods study. Anatomical Sciences Education, 12(2), 129-137. | Germany | "The purpose of this study was to explore the professional and personal competencies peer tutors of gross anatomy developed as a result of their tutoring activities. It was hypothesized that because of the unique and challenging environment of the gross anatomy course, tutors acquire competencies that match, and even go beyond those described in some of the competency catalogues used for curriculum development." | Students (medical students who are peer tutors) | mixed methods | Individual interviews and questionnairs | 8 to 12 months | 24 | 72 inter-views and 72 questionnaires |
| Alves-Costa, F., Hamilton-Giachritsis, C., Christie, H., & Halligan, S. L. (2018). Self-perception of adaptation among homicidally bereaved individuals following a psychoeducational intervention: a UK longitudinal qualitative study. BMJ Open, 8(8), e020443-e020443. | UK | "Thus, this study aimed to contribute to the literature by gathering the individuals’ perceptions about how they adjust posthomicide longitudinally. Furthermore, it sought to understand their perspectives on benefits 1. What changes occurred over time? 2. What was their perception of the benefits of a residential intervention?" | Patients (participants of a psychoeducational intervention following traumatic experiences particularly homicidal bereavement for those affected by serious crime) | mixed methods & qualitative longitudinal research | Individual interviews | up to 5 years | 14 | 29 interviews |
| Andersen, I. C., Thomsen, T. G., Bruun, P., Bødtger, U., & Hounsgaard, L. (2017a). The experience of being a participant in one's own care at discharge and at home, following a severe acute exacerbation in chronic obstructive pulmonary disease: a longitudinal study. International Journal of Qualitative Studies on Health and Well-Being, 12(1), 1371994-1371994. | Denmark | "To explore COPD [chronic obstructive pulmonary disease] patients’ experiences of participating in their care in the transitional period around discharge from hospital and in their own subsequent day-to-day care at home following a severe AECOPD [acute exacerbation in chronic obstructive pulmonary disease]." | Patients (chronic obstructive pulmonary disease patients’) | Phenomenology & qualitative longitudinal research | observations, individual interviews and joint interviews | Up to 18 months (range 4 days to 18 months) | 15 | Unclear, 15 observations and at least 20 interviews |
| Andersen, I. C., Thomsen, T. G., Bruun, P., Bødtger, U., & Hounsgaard, L. (2017b). Patients' and their family members' experiences of participation in care following an acute exacerbation in chronic obstructive pulmonary disease: A phenomenological-hermeneutic study. Journal Of Clinical Nursing, 26(23), 4877-4889. | Denmark | "The aim of this study was to explore COPD [chronic obstructive pulmonary disease] patients’ and their family members’ experiences of both participation in care during hospitalisation for an AECOPD[acute exacerbation in chronic obstructive pulmonary disease], and of the subsequent day-to-day care at home." | Several: Patients and family members (COPD patients’ and their family members’) | Phenomenology | Observations, informal interviews, and individual interviews | 11 to 18 months (range 4 days to 18 months) | 27 | 15 observations including informal interviews, 32 individual interviews, 10 joint interviews |
| Andersen, I. C., Thomsen, T. G., Bruun, P., Bødtger, U., & Hounsgaard, L. (2018). Between hope and hopelessness: COPD patients' and their family members' experiences of interacting with healthcare providers – a qualitative longitudinal study. Scandinavian Journal of Caring Sciences, 32(3), 1197-1206. | Denmark | "…explore the meaning of COPD patients’ and their family members’ experiences of interacting with healthcare providers to their daily self-management over time." | Several: Patients and family members (COPD patients and family members) | Phenomenology & qualitative longitudinal research | Participant observations, individual and joint interviews | up to 18 months | 17 | 22 interviews |
| Armuand, G., Wettergren, L., Nilsson, J., Rodriguez-Wallberg, K., & Lampic, C. (2018). Threatened  fertility: A longitudinal study exploring experiences of fertility and having children after cancer treatment. European Journal of Cancer Care, 27(2), e12798-e12798. | Sweden | "The aim of this study was, therefore, to explore how men and women experience the threat of infertility and their thoughts about having children after cancer during the first 2 years following diagnosis." | Family members (couples with risk of infertility after cancer diagnosis) | qualitative longitudinal research | Individual interviews | 2 years | 21 | 37 interviews |
| Arnolds, M., Xu, L., Hughes, P., McCoy, J., & Meadow, W. (2018). Worth a try? Describing the experiences of families during the course of care in the neonatal intensive care unit when the prognosis is poor. The Journal of Pediatrics, 196, 116-122.e113. | US | "This study illuminates the experience of families during an infant’s admission as well as after discharge or death." | Family members (parents to infants) | qualitative longitudinal research | Joint interviews | average of 435 days (range, 259-750 days) | 52 | 43 interviews |
| Asada, Y., Gilmet, K., Welter, C., Massuda-Barnett, G., Kapadia, D. A., & Fagen, M. (2019). Applying theory of change to a structural change initiative: Evaluation of model communities in a diverse county. Health Education & Behavior, 46(3), 377-387. | US | "...this article examines the application of a ToC framework [the theory of change] that guided planning, implementation, and evaluation of the Model Communities grant program (2011-2012) that was funded to suburban Cook County as one component of the CPPW [Communities Putting Prevention to Work] initiative." | Several: Policy-makers and managers (community-based organization receiving funds to work for health behaviours) | Case study | Individual interviews | Unclear | Unclear, six data collection sites, total number of participants is not described | 97 interviews |
| Asamane, E. A., Greig, C. A., Aunger, J. A., & Thompson, J. L. (2019). Perceptions and factors influencing eating behaviours and physical function in community-dwelling ethnically diverse older adults: A longitudinal qualitative study. Nutrients, 11(6). | UK | "The present study uses a longitudinal qualitative design to: (1) identify and compare factors influencing eating behaviours and physical function among ethnic older minorities living in Birmingham, United Kingdom; and (2) understand how these factors and their association with healthy eating and physical function changed over 8 months" | People in the community (older people from ethnic minorities) | Phenomenology & qualitative longitudinal research | Individual interviews | 8 months | 92 | 173 interviews |
| Bagot, K. L., Moloczij, N., Barclay-Moss, K., Vu, M., Bladin, C. F., & Cadilhac, D. A. (first published 2018). Sustainable implementation of innovative, technology-based health care practices: A qualitative case study from stroke telemedicine. Journal of Telemedicine and Telecare, 2020, 26(1–2) 79–91. | Australia | "The aim of this study was to identify factors to support the sustainability of an innovative technology-based programme beyond the initial implementation phases." | Health care providers (health-care professionals) | Case study | Individual interviews | 6 months | 25 | 49 interviews |
| Balmer, D. F., Devlin, M. J., & Richards, B. F. (2017). Understanding the relation between medical students' collective and individual trajectories: An application of habitus. Perspectives on Medical Education, 6(1), 36-43. | US | "Thus, we posed another research question in this secondary analysis, ‘How might the concept of habitus speak to the relation between collective trajectories and individual trajectories of medical students?’" | Students (medical students) | Case study | Individual interviews | about 30 months | 19 | 95 narratives derived from interviews |
| Baloh, J., Zhu, X., & Ward, M. M. (2018). Types of internal facilitation activities in hospitals implementing evidence-based interventions. Health Care Management Review, 43(3), 229-237. | US | "To examine internal facilitation activities at ten critical access hospitals in rural Iowa during their implementation of TeamSTEPPS, a patient safety intervention, and to identify characteristics that distinguish different types of facilitation activities" (from abstract) | Several: Health-care providers and managers (health-care providers and managers at 10 hospitals) | qualitative longitudinal research | Individual interviews | 2 years | 77 key informants | 281 interviews |
| Baretta, D., Perski, O., & Steca, P. (2019). Exploring users' experiences of the uptake and adoption of physical activity apps: Longitudinal qualitative study. JMIR Mhealth And Uhealth, 7(2), e11636-e11636. | Italy | "The aim of this study was to guide the selection of design features to implement in Physical Activity [PA] apps for nonclinical, adult populations. Through a combination of think-aloud methodology and in-depth interview techniques, this study examined (1) what features potential users expect to be important for engagement with PA apps during first exposure to never-used, randomly allocated, and commercially available PA app and (2) what features are judged to be important for supporting engagement and satisfactory experiences after 2 weeks’ usage of the same PA app." | People in the community (adults using physical activity apps) | Qualitative longitudinal research | Individual interviews and think aloud interviews | 2 weeks | 20 | 37 interviews |
| Barros Ferreira, E., Oliveira de Almeida Marques da Cruz, F., Alves Costa de Jesus, C., Moura Pinho, D. L., Kamada, I., & Diniz dos Reis, P. E. (2017). Telephone contact as a strategy for the promotion of comfort to the patient submitted to chemotherapy. Journal of Nursing UFPE, 11(5), 1936-1942. | Brazil | "This study aimed to monitor the adverse effects of antineoplastic chemotherapy in patients undergoing outpatient treatment and to describe the telephone follow-up as a strategy to provide comfort, according to the assumptions of Katherine Kolcaba." | Patients (patients undergoind chemotherapy) | not described | Observations (recorded phone consultations) | 7 weeks | 21 | 147 telephone contacts |
| Barthel, S., Belton, S., Raymond, C. M., & Giusti, M. (2018). Fostering children's connection to nature through authentic situations: The case of saving salamanders at school. Frontiers in Psychology, 9, 928-928. | Sweden | "The aim of this paper is to increase our understanding about if, how and by which means children’s affective relationships with nature change by taking part in a nature conservation project during school hours, and if such a shift persists 2 years post-participation. Does participating in the Salamander Project at school strengthen children’s connection to nature? If so, how do children learn to create affective relations with nature? Which specific situations might encourage or enable stronger affective relationships with nature? Do affective relations persist 2 years after the project?" | Students (10 year old children) | qualitative longitudinal research | Individual interviews, open-ended questions in a questionnaire, and field observations | 2 years | 57 | 106 questionaries, 49 interviews, 9 field observations |
| Bélanger, M., Wolfe Phillips, E., O'Rielly, C., Mallet, B., Aubé, S., Doucet, M., Couturier, J., Mallet, M., Martin, J., Gaudet, C., Murphy, N. & Brunet, J. (2017). Longitudinal qualitative study describing family physicians' experiences with attempting to integrate physical activity prescriptions in their practice: 'It's not easy to change habits'. BMJ Open, 7(7), e017265-e017265. | Canada | "We aimed to explore how their [*family physicians who were new recipients of physical activity prescription pads]* prescription habits changed (if at all) over time and to identify barriers and enablers to writing physical activity prescriptions for their patients." | Health care providers (family physicians) | Qualitative longitudinal research | Individual interviews | 12 months | 11 | 43 interviews |
| Bengtsson, M., Sjöblom, Y., & Öberg, P. (2018). Young care leavers' expectations of their future: A question of time horizon. Child & Family Social Work, 23(2), 188-195. | Sweden | "The aim of this paper is to study young care leavers' own expectations of their future during their transition from care to independent adulthood. How, while still in care, do they express their expectations of this transition, and do these expectations change while they are taking their first step towards an independent life?" | People in the community (young people) | qualitative longitudinal research | individual interviews | 6 to 9 months | 15 | 30 interviews |
| Bernays, S., Paparini, S., Seeley, J., & Rhodes, T. (2017). "Not taking it will just be like a sin": Young people living with HIV and the stigmatization of less-than-perfect adherence to antiretroviral therapy. Medical Anthropology, 36(5), 485-499. | Uganda, US, UK, and Ireland | "To overcome the challenge of evaluating meaningfulness of treatment effect from a patient-reported perspective in the context of this rare cancer, a mixed methods approach that followed a convergent design was used [28]." | Patients ( young people living with HIV) | Qualitative longitudinal research | Individual interviews, field notes, observations | Unclear, up to three interviews during a trial | 43 | 102 interviews |
| Bernet, M., Sommerhalder, K., Mischke, C., Hahn, S., & Wyss, A. (2019). "Theory does not get you from bed to wheelchair": A qualitative study on patients' views of an education program in spinal cord injury rehabilitation. Rehabilitation Nursing, 44(5), 247-253. | Switzerland | "The purpose of the current study was, therefore, to evaluate the new nurse-guided Patient Education [PE] program in Spinal Cord Injury rehabilitation, with a particular focus on the patients' perspectives and experiences. The study focused on two areas: (1) to analyze the program’s impact on patients’ preparation for their everyday life at home after rehabilitation and (2) to reveal which aspects of the PE program were most valuable from the participants’ perspective" | Patients (persons with spinal cord injury) | Qualitative longitudinal research | Individual interviews | 5 to 6 months | 10 | 20 interviews |
| Beryl, L. L., Rendle, K. A. S., Halley, M. C., Gillespie, K. A., May, S. G., Glover, J., Yu, P., Chattopadhyay, R. & Frosch, D. L. (2017). Mapping the decision-making process for adjuvant endocrine therapy for breast cancer. Medical Decision Making, 37(1), 79-90. | US | "To help fill this gap in understanding, we present data from a longitudinal, qualitative study of women undergoing breast cancer treatment. We pay particular attention to patterns of uncertainty and change in women’s decisions over time. We use detailed, qualitative data to characterize the multiple dimensions of the decision-making process and to consider how the chronic care decision-making process around hormone therapy differs from other acute decision making processes in breast cancer treatment. Our analysis suggests the importance of tracking ‘‘decisional resolve,’’ that is, a patient’s firm determination to maintain her decision to take or not take hormone therapy over time." | Patients (women undergoing breast cancer treatment) | qualitative longitudinal research | Individual interviews | avarage 260 days | 41 | Unclear |
| Bharmal, M., Guillemin, I., Marrel, A., Arnould, B., Lambert, J., Hennessy, M., & Fofana, F. (2018). How to address the challenges of evaluating treatment benefits-risks in rare diseases? A convergent mixed methods approach applied within a Merkel cell carcinoma phase 2 clinical trial. Orphanet Journal of Rare Diseases, 13(1), 95-95. | Germany | "To overcome the challenge of evaluating meaningfulness of treatment effect from a patient-reported perspective in the context of this rare cancer, a mixed methods approach that followed a convergent design was used [28]. In this study, qualitative interviews were performed with the patients participating in the JAVELIN Merkel 200 trial; in parallel, patients’ overall response by Independent Endpoint Review Committee (IERC) per Response Evaluation Criteria In Solid Tumors version 1.1 (RECIST) was determined clinically to report patients’ tumour response status [29]. Data from both the patient interviews and the clinical evaluations were then merged to look for correspondence between the qualitative outcomes data and the clinical and patient-reported quantitative outcomes data." | Patients (adults who had chemotherapy-refractory, with histologically confirmed Merkel cell carcinoma, rare skinn cancer) | mixed methods | Individual interviews | 25 weeks | 19 | 41 interviews |
| Bilodeau, K., Tremblay, D., & Durland, M.-J. (2019). Return to work after breast cancer treatments: Rebuilding everything despite feeling "in-between". European Journal of Oncology Nursing, 41, 165-172. | Canada | "We propose targeting a turning point in the survival experience, i.e. the end of active cancer treatments. The goal of this study is therefore to describe the Return To Work journey of Breast cancer survivors from the end of active treatments through their return to work and job retention." | Patients (women after breast cancer treatment) | Interpretative Description method & qualitative longitudinal research | Individual interviews | Unclear. Total data collection for the study were within 9 months. Unclear regarding the individual cases. | 9 | 23 interviews |
| Birt, L., Poland, F., Charlesworth, G., Leung, P., & Higgs, P. (2019). Relational experiences of people seeking help and assessment for subjective cognitive concern and memory loss. Aging & Mental Health, 1-9. | UK | "...understand the experiences of those who seek help from a primary care doctor for subjective cognitive concerns, but who do not receive a cognitive assessment. We also report the experiences of patients who have experienced cognitive assessments from diverse health providers, reporting processes and procedures which disrupt their trust in the process of cognitive assessment." | Patients (people experience cognitive concerns) | interpretive constructivist approach | Individual interviews | 12 to 15 months | 41 | 77 interviews |
| Blagden, N., & Wilson, K. (2019). "We're all the same here"- Investigating the rehabilitative climate of a re-rolled sexual offender prison: A qualitative longitudinal study. Sexual Abuse, 1-24. | UK | "This study aims to explore the qualitative changes in the rehabilitative climate of a re-rolled prison (in this case, a general prison turned into a prison only for individuals who have sexually offended) from just after the re-roll to a year later. 1. To understand how individuals with sexual convictions experience a prison for only that client group, the challenges they face, and the opportunities to change and whether such experiences change over time. 2. To investigate the perspectives of prisoners on the purpose of the prison, its regime, and climate and whether this changes over time." | People in the community (individuals in prison for a sexual crime) | Qualitative longitudinal research | Individual interviews | 12 months | 10 | 20 interviews |
| Bolier, M., Doulougeri, K., de Vries, J., & Helmich, E. (2018). 'You put up a certain attitude': a 6-year qualitative study of emotional socialisation. Medical Education, 52(10), 1041-1051. | the Netherlands | "The aim of this longitudinal qualitative study was to gain a better understanding of the socialisation of emotion in the process of becoming a doctor." | Students (medical students) | qualitative longitudinal research | Individual interviews | 6 years | 17 | 48 interviews |
| Bomsta, H., & Sullivan, C. M. (2018). IPV survivors’ perceptions of how a flexible funding housing intervention impacted their children. Journal of Family Violence, 33(6), 371-380. | US | "The current study, then, presents IPV survivors’ perceptions of how a brief intervention designed to enhance their safety and housing stability also impacted their children’s well-being." | People in the community (mothers experienced intimate partner violence) | qualitative, longitudinal evaluation of a brief intervention | Individual interviews | 6 months | 42 | 117 interviews |
| Boström, M., Ernsth Bravell, M., Björklund, A., & Sandberg, J. (2017). How older people perceive and experience sense of security when moving into and living in a nursing home: A case study. European Journal of Social Work, 20(5), 697-710. | Sweden | "Therefore, a longitudinal design with in-depth interviews and observations was used in order to highlight sense of security by the older person when moving into and living in nursing home." | People in the community (older persons) | Case study | Individual interviews and observations | 3 to 4 months | 3 | 9 interviews and field notes |
| Braaf, S., Ameratunga, S., Ponsford, J., Cameron, P., Collie, A., Harrison, J., Ekegren, C., Christie, N., Nunn, A., & Gabbe, B. (2019). Traumatic injury survivors' perceptions of their future: A longitudinal qualitative study. Disability and Rehabilitation, 1-11. | Australia | "...this study aims to explore seriously injured adults’ perceptions of their future including their concerns, anxieties, coping mechanisms, and sources of resilience over time." | Patients (seriously injured adults) | Qualitative longitudinal research | Individual interviews | about 2 years | 66 | 186 interviews |
| Bradley, E. H., Brewster, A., L., McNatt, Z., Linnander, E. L., Cherlin, E., Fosburgh, H., Ting, H. H., Curry, L. A. (2018). How guiding coalitions promote positive culture change in hospitals: a longitudinal mixed methods interventional study. BMJ Quality & Safety, 27(3), 218-225. | US | "Accordingly, we sought to understand what distinguished hospitals that succeeded to shift culture substantially and to reduce 30-day RSMR [risk-standardised mortality rate] after AMI [acute myocardial infarction] through participation in the LSL [Leadership Saves Lives] collaborative." | Several: Health-care providers and managers (staff from multiple departments and professions) | mixed methods | Questionnairs, individual interviews, and observations | 18 months | 197 | 468 questionnairs, 624 interviews |
| Brietzke, M., & Perreira, K. (2017). Stress and coping: Latino youth coming of age in a new latino destination. Journal of Adolescent Research, 32(4), 407-432. | US | "Our study aimed to contextualize the processes of stress and coping among Latino adolescents growing up in an emerging Latino destination in the US—North Carolina (NC)." (from abstract) | People in the community (Latino adolescents) | Qualitative longitudinal research | Individual interviews | 3 years | 12 | 24 interviews |
| Bright, F. A. S., Kayes, N. M., McPherson, K. M., & Worrall, L. E. (2018). Engaging people experiencing communication disability in stroke rehabilitation: A qualitative study. International Journal of Language & Communication Disorders, 53(5), 981-994. | New Zealand | "The aim of this research was to develop rich understandings of the process of engagement for people experiencing communication disability after stroke and, in particular, to examine how rehabilitation practitioners worked to engage patients throughout rehabilitation." | Several: patients and health-care providers (people experiencing communication disability after stroke and their providers) | underpinned by the Voice Centred Relational Approach | Observations, short debrie interviews,and individual interviews | 2 weeks | 31 | 160 observations over 147 h, 108 interviews, and 5 stimulated recall interviews with practitioner participants |
| Brooks, H., Lovell, K., Bee, P., Fraser, C., Molloy, C., & Rogers, A. (2019). Implementing an intervention designed to enhance service user involvement in mental health care planning: A qualitative process evaluation. Social Psychiatry & Psychiatric Epidemiology, 54(2), 221-233. | UK | "This manuscript reports on the nested qualitative process evaluation informed by implementation theory which aimed to explore the impact of the EQUIP training package to enhance user involvement in care planning." | Several: Patients, family members and health-care professionals (service users, carers and mental health professionals) | qualitative process evaluation | Individual interviews | 12 months | 54 | 134 interviews |
| Brown, D. (2019). Changes in communication tensions for men facing prostate cancer: A longitudinal study. Journal of Communication in Healthcare, 12(1), 44-53. | New Zealand | "In an effort to better understand tensions over time, the principle aims of the present study are to establish if and how they change after men have undergone a prostate biopsy. Three research questions guided this study: RQ1: How were the communication tensions of a group of men waiting for the result of a prostate biopsy initially (T1) resolved three years later (T2)? RQ2: What new communication tensions emerged three years after the biopsy (T2)? RQ3: What competing discourses underpin the communication tensions in this cohort of men at T1 as compared to T2?" | Patients (men undergone prostate biopsy) | not described | Individual interviews | About 3 years | 25 | 50 interviews |
| Busza, J., Dauya, E., Bandason, T., Simms, V., Chikwari, C. D., Makamba, M., Mchugh, G., Munyati, S., Chonzi, P., & Ferrand, R. A. (2018). The role of community health workers in improving HIV treatment outcomes in children: Lessons learned from the ZENITH trial in Zimbabwe. Health Policy and Planning, 33(3), 328-334. | Zimbawe | "The aim was to give a voice to the central actors of a CHW [community health workers] programme that successfully met its goals, reflect on how use of a criteria-based framework during intervention design affected CHWs’ [community health workers] delivery, job satisfaction and motivation, and consider implications for the programme’s future scale-up and adoption in other settings." | Health care providers (community health workers) | not described | Individual interviews | 2 years | 19 | 57 interviews |
| Busza, J., Simms, V., Dziva Chikwari, C., Dauya, E., Bandason, T., Makamba, M., McHughc, G., & Ferrand, R. A. (2018). “It is not possible to go inside and have a discussion”: How fear of stigma affects delivery of community-based support for children’s HIV care. AIDS Care, 30(7), 903-909. | Zimbawe | "We investigated how stigma affected a community-based intervention to support caregivers of children newly diagnosed with HIV in Harare, Zimbabwe. Specifically, we assessed children’s, caregivers’, and CHWs’ [community health workers] perceptions of how HIV-related stigma affected implementation." | Several: Patients, family-members and health-care providers (children with HIV, caregivers, andcommunity health workers)) | qualitative longitudinal research | Individual interviews | 18 months | 71 | 91 interviews |
| Cain, C. L., Frazer, M., & Kilaberia, T. R. (2019). Identity work within attempts to transform healthcare: Invisible team processes. Human Relations, 72(2), 370-396. | US | "In this article, we ask: How do workers in a new healthcare team negotiate multiple identification targets, including their previous professional identities as well as their new team identities? What contextual events produce shifts in how workers identify? What do these shifts tell us about work identities, teams and organizational change?" | Health care providers (workers in health-care teams) | not described | Diaries | 30 weeks | Unclear, probably 8 to 14 participants | 176 audio dairy recordings |
| Cameron, A., Johnson, E. K., Lloyd, L., Evans, S., Smith, R., Porteus, J., Darton, R., & Atkinson, T. (2019). Using longitudinal qualitative research to explore extra care housing. International Journal of Qualitative Studies on Health and Well-Being, 14(1), 1593038-1593038. | UK | "Using a longitudinal qualitative research (LQR) approach, the aim of The Provision of Social Care in Extra Care Housing (ECHO) project was to investigate how care is negotiated and delivered in ECH. Focusing on the “extra care” element of extra care housing services, the ECHO project explored the perspectives of residents on their changing care needs and their experiences of being cared for." | Several: people in the community, managers,social service providers, managers (residents, managers, care staff and local commissioners of housing and care) | Qualitative longitudinal research | Unstructured observations, individual interviews, and documents (e.g., annual reports) | 20 months | 51 | 164 interviews |
| Campbell, V., & Nolan, M. (2019). 'It definitely made a difference': A grounded theory study of yoga for pregnancy and women's self-efficacy for labour. Midwifery, 68, 74-83. | UK | "The aim was to generate a theory, grounded in women’s voices, about which aspects of Yoga for Pregnancy (YfP) are effective in enhancing women’s ability to manage labour." | People in the community (pregnant woman) | Grounded theory | Individual interviews | Differed between partricipants, up to 7.5 months | 22 | 45 interviews |
| Carduff, E., Kendall, M., & Murray, S. A. (2018). Living and dying with metastatic bowel cancer: Serial in-depth interviews with patients. European Journal of Cancer Care, 27(1). | UK | "This paper reports the longitudinal experiences, perceptions and service use of patients with metastatic colorectal cancer." | Patients (patients with metastatic colorectal cancer) | Narrative research | Individual interviews | one year | 16 | 36 interviews |
| Carusone, S. C., O'Leary, B., McWatt, S., Stewart, A., Craig, S., & Brennan, D. J. (2017). The lived experience of the hospital discharge "Plan": A longitudinal qualitative study of complex patients. Journal of Hospital Medicine, 12(1), 5-10. | Canada | "A longitudinal case study approach was used, with multiple sources of data, to understand the clinical context and discharge plans in relation to the lived experience of patients over time, exploring potential misalignment and areas for improvement." | Patients (complex patients) | Case study & qualitative longitudinal research | individual interviews, medical chart abstraction, and review of discharge summaries. | about 30 days | 9 | 12 interviews |
| Castro, A., & Andrews, G. (2018). Nursing lives in the blogosphere: A thematic analysis of anonymous online nursing narratives. Journal of Advanced Nursing, 74(2), 329-338. | Canada | "The purpose of this study was to explore the work-life narratives of nurses through a thematic analysis of the nursing accounts they post in their public blogs. The overall research question was, 'when nurses who work in traditional healthcare settings have the protection of online anonymity, how do they describe their nursing insights and experiences on blog platforms?' " | Health care providers (nurses) | qualitative description | blog texts | 12 months | 4 | 520 blog posts |
| Cherry, M. G., Salmon, P., Byrne, A., Ullmer, H., Abbey, G., & Fisher, P. L. (2019). Qualitative evaluation of cancer survivors' experiences of metacognitive therapy: A new perspective on psychotherapy in cancer care. Frontiers in Psychology, 10, 949-949. | UK | "In this study, we report the findings of qualitative research nested in an open trial of MCT [Metacognitive Therapy] for anxiety and depression in adult cancer survivors. We also interviewed consenting patients at each time point, to explore qualitatively: (i) how they understood and experienced the intervention; (ii) once treatment ended, how, and to what extent over the follow-up period, did patients transfer what they had learned across the range of emotional challenges arising during survivorship; and (iii) what characterized any patients who did not benefit?" | Patients (adult cancer survivors with depression or anxiety) | Qualitative longitudinal research | Individual interviews | 6 months | 19 | 43 interviews |
| Choi, J., Lingler, J. H., Donahoe, M. P., Happ, M. B., Hoffman, L. A., & Tate, J. A. (2018). Home discharge following critical illness: A qualitative analysis of family caregiver experience. Heart & Lung, 47(4), 401-407. | US | "The purpose of this study was to longitudinally describe the varying challenges and needs of family caregivers of ICU [intensive care units] survivors related to patients’ home discharge." | Family members (family caregivers of ICU survivors) | descriptive qualitative study | Individual interviews | 4 months | 20 | 37 interviews |
| Chu, H., Westbrook, R. A., Njue-Marendes, S., Giordano, T. P., & Dang, B. N. (2019). The psychology of the wait time experience - what clinics can do to manage the waiting experience for patients: a longitudinal, qualitative study. BMC Health Services Research, 19(1), 459-459. | US | "Here in, we examine contextual factors and potential intervening variables that can shape the manner in which patients may respond to different waits. In addition, this study aims to identify actions providers and clinics can take to promote positive wait time experiences and mitigate negative ones." | Patients (People with HIV) | Qualitative longitudinal research | Individual interviews | 6 to 12 months | 56 | 138 interviews |
| Ciclitira, K., Starr, F., Payne, N., Clarke, L., & Marzano, L. (2017). A sanctuary of tranquillity in a ruptured world: Evaluating long-term counselling at a women’s community health centre. Feminism & Psychology, 27(4), 530-552. | UK | "The main aim of this study was to explore how service users make sense of long term counselling in a women-only service in the context of their gendered experiences and complex needs." | People in the community (female service users) | not described | Individual interviews | unclear, before and after counseling | 59 | 66 interviews |
| Clarke, G., Fistein, E., Holland, A., Tobin, J., Barclay, S., & Barclay, S. (2018). Planning for an uncertain future in progressive neurological disease: a qualitative study of patient and family decision-making with a focus on eating and drinking. BMC Neurology, 18(1), 115-115. | UK | "We therefore undertook a study of patients and their families with a range of progressive neurological diseases. We investigated their experiences and views on decision-making concerning their care as their disease progressed, with a focus on problems with eating and drinking. The key research question was: How do patients and their family members make decisions about their future care, with a particular focus on mealtimes, eating and drinking?" | Several: Patients and family members (patients and their families with a range of progressive neurological diseases) | Qualitative longitudinal research | Individual interviews, observation and informsal conversation | 3 to 12 months depending upon disease | 29 | Unclear |
| Clermont, A., Kodish, S. R., Matar Seck, A., Salifou, A., Rosen, J., Grais, R. F., & Isanaka, S. (2018). Acceptability and utilization of three nutritional supplements during pregnancy: Findings from a longitudinal, mixed-methods study in Niger. Nutrients, 10(8). | Niger | "The objective of this paper is to examine the factors influencing acceptability and utilization of these three supplements among a rural population in southern Niger using a longitudinal, mixed-methods design. In this paper, we discuss the typical consumption, perceived benefits, facilitating factors, and barriers to appropriate utilization reported by participants for each of the three supplement types over the course of pregnancy. We also examine household and community member perceptions of supplement utilization and triangulate qualitative findings with quantitative household utilization data from unannounced spot checks." | Several: Pregnant women, family members and people in the community (pregnant woman, household members, and community members) | mixed methods | Individual interview and focus group interviews | 6 months | unclear, over 100 participants | 114 individual interviews, 26 focus group interviews |
| Coombe, J., Harris, M. L., & Loxton, D. (2019). Motivators of contraceptive method change and implications for long-acting reversible contraception (non-)use: A qualitative free-text analysis. Sexual & Reproductive Healthcare, 19, 71-77. | Australia | "...this analysis sought to develop a greater understanding of the motivators of method change over time for young Australian women using three waves of survey data from the Contraceptive Use, Pregnancy Intention and Decisions (CUPID) Study. Using responses to an open-ended question regarding reasons for contraceptive change, we were particularly interested in exploring what these comments could tell us (if anything) about long-acting reversible contraception (LARC; IUDs, contraceptive implants, contraceptive injections), (non-)use among young women." | People in the community (young women) | not described | Free-text comments from questionnairs | About 12 months | 512 | 740 free-text comments |
| Coombs, M. A., Parker, R., & de Vries, K. (2017). Managing risk during care transitions when approaching end of life: A qualitative study of patients’ and health care professionals’ decision making. Palliative Medicine, 31(7), 617-624. | New Zealand | "To describe decision-making processes that influence transitions in care when approaching the end of life." | Several: Patients and family members (Patients in palliative care who approache end of life, and their family members) | Qualitative longitudinal research | Field observation and individual interviews. | 3 to 4 months | 29 | 40 interviews |
| Corepal, R., Best, P., O'Neill, R., Tully, M. A., Edwards, M., Jago, R., Miller, S.J., Kee, F., Hunter, R. F. (2018). Exploring the use of a gamified intervention for encouraging physical activity in adolescents: a qualitative longitudinal study in Northern Ireland. BMJ Open, 8(4), e019663-e019663. | UK (northern Ireland) | "The aim of this study was to explore the views and experiences of adolescents who participated in a gamified PA [physical activity] intervention based on Self-determination Theory (SDT), and the temporal changes of these views and experiences over the 1-year study period. Study objectives included: 1. To explore key aspects of a gamified PA intervention over a 1-year period using a qualitative longitudinal research (QLR) method. 2. To discuss key issues relating to the intervention, such as PA opportunities/barriers, the value of competition and types of rewards and so on. 3. To explore the key influences of PA and to determine who benefited from the intervention, how and why it worked for them. 4. To qualitatively chart changes in behaviours, opinions or views as a result of participating in the intervention." | People in the community (adolescents who participated in a gamified PA intervention) | qualitative longitudinal research | Focus group interviews | 12 months | 19 | 12 focus group interviews |
| Côté-Arsenault, D., & Denney-Koelsch, E. (2018). “Love is a choice”: Couple responses to continuing pregnancy with a lethal fetal diagnosis. Illness, Crisis & Loss, 26(1), 5-22. | US | "Hence, the purpose of this study is to describe pregnant couples’ responses and relationships during pregnancy and after birth when they choose to continue a pregnancy with an LFD [Lethal Fetal Diagnosis]. A secondary goal is to examine similarities and differences in these responses within the couple." | Family members (pregnant couples’) | longitudinal naturalistic study | Individual interviews and joint interviews | Unclear: "Ninety interviews were conducted between 2012 and 2014; each parent participated in one to five interviews" | 30 | 90 |
| Cresswell, K. M., Mozaffar, H., Lee, L., Williams, R., & Sheikh, A. (2017). Safety risks associated with the lack of integration and interfacing of hospital health information technologies: A qualitative study of hospital electronic prescribing systems in England. BMJ Quality & Safety, 26(7), 530-541. | UK | "As part of an English programme of research, we explored the social and technical challenges relating to integration and interfacing experienced by early adopter hospitals of standalone and hospital-wide multimodular integrated electronic prescribing (ePrescribing) systems." (from abstract) | Several: health-care providers, managers, policy-makers (for example users, implementers and software suppliers) | longitudinal, qualitative, multisite case study | Individual interviews, documentary data, observations and expert roundtable discussions | Up to 3 years (differed between sites) | Unclear | 173 interviews, 24 observations, 17 documents, and 2 whole-day expert round-table discussions. |
| Curry, L. A., Brault, M. A., Linnander, E. L., McNatt, Z., Brewster, A. L., Cherlin, E., Peterson Flieger, S., Ting, H. H. & Bradley, E. H. (2018). Influencing organisational culture to improve hospital performance in care of patients with acute myocardial infarction: A mixed-methods intervention study. BMJ Quality & Safety, 27(3), 207-217. | US | "We designed a 2-year intervention, Leadership Saves Lives (LSL),26 directed at fostering changes in hospital organisational culture that might contribute to reductions in RSMRs [risk-standardised mortality rates] for patients with AMI [acute myocardial infarction]. Our study addresses limitations of prior research through a longitudinal design in a diverse sample of hospitals, the use of robust quantitative and qualitative measures of culture, and the inclusion of an important clinical outcome." | Several: Health-care providers, managers (participants from different departments and professions) | mixed methods | Questionnairs, individual interviews, and ethnographic observations | 18 months | 197 | 514 questionnairs, 56 hours of observation, 393 interviews. |
| Daker-White, G., Hays, R., Blakeman, T., Croke, S., Brown, B., Esmail, A., & Bower, P. (2018). Safety work and risk management as burdens of treatment in primary care: Insights from a focused ethnographic study of patients with multimorbidity. BMC Family Practice, 19, 155. | UK | "The aims of the study were to describe the safety issues identified in a cohort of primary care patients with multimorbidity; and, to explore the clinical and social context in which patient safety concerns arise and play out. These objectives were addressed via a longitudinal, multi-method qualitative study in the form of a focused ethnography [44]. The principle aim of the analysis was to construct a line of argument concerning the circumstances under which patient agency in safety monitoring might be bolstered." | Several: Patients, family members and health-care providers (primary care patients with multimorbidity, spouses, health-care providers) | longitudinal multimethod qualitative study, ethnography & case study | Individual interviews, observations, and health care diaries | 24 months | 6 | Unclear |
| Dambha-Miller, H., Silarova, B., Irving, G., Kinmonth, A. L., & Griffin, S. J. (2018). Patients' views on interactions with practitioners for type 2 diabetes: A longitudinal qualitative study in primary care over 10 years. The British Journal Of General Practice, 68(666), e36-e43. | UK | "This study aims to explore patient views on factors within patient–practitioner interactions that are of significance to them after diagnosis, and over a 10-year experience of living with the disease." | Patients (patients with diabetes type 2) | qualitative longitudinal research | Open ended question in questionnairs | 10 years | 311 | 412 text extracts from open ended questions |
| Dang, B. N., Westbrook, R. A., Njue, S. M., & Giordano, T. P. (2017). Building trust and rapport early in the new doctor-patient relationship: A longitudinal qualitative study. BMC Medical Education, 17(1), 32-32. | US | "...this study aims to identify what patients see as the most critical elements for building trust and rapport from the outset." | Patients (people with HIV) | Qualitative longitudinal research | Individual interviews | 6 to 12 months | 21 | 57 interviews |
| Danneris, S. (2018). Ready to work (yet)? Unemployment trajectories among vulnerable welfare recipients. Qualitative Social Work, 17(3), 355-372. | Denmark | "A central aim of the article is to introduce the client perspective as a way of increasing and supplementing our knowledge about policy delivery in terms of understanding not only what services and benefits clients receive but also how they are received and the consequences of receiving them (Borghi and Van Berkel, 2007; Larsen, 2013). Investigating the fabric and textures of the perceived policy implications and their longitudinal interrelations from the client perspective will illuminate corners of the concept of employability where the current map ceases and reveal new, perhaps conflicting, understandings and dilemmas (Verd and Lo´ pez, 2011: 3)." | People in the community (vulnerable welfare recipients) | qualitative longitudinal research | individual interviews, observations, and informal conversations | 24 months | 36 | over 2000 pages of transcribed observations, interviews and field notes |
| Dattalo, M., Wise, M., Ford Li, J. H., Abramson, B., & Mahoney, J. (2017). Essential resources for implementation and sustainability of evidence-based health promotion programs: A mixed methods multi-site case study. Journal of Community Health, 42(2), 358-368. | US | "This study examines the varying abilities of intervention sites from the randomized trial to implement and sustain SO [Stepping On is a falls prevention program] and CDSMP [Chronic Disease Self-Management Program] workshop delivery over 3 years and compares different approaches to preparing for workshop implementation by level of sustainability. | Policy-makers (coaches and country change leaders) | mixed methods and case study | Individual interviews, documents (e.g., annual county reports), and open-ended questions in questionnairs | 3 years | 9 | 15 interviews, 22 questionnairs, and 7 reports |
| Davies, K. E., Marshall, J., Brown, L. J. E., & Goldbart, J. (2017). Co-working: Parents’ conception of roles in supporting their children’s speech and language development. Child Language Teaching & Therapy, 33(2), 171-185. | UK | "The current study aimed to identify parents’ conception of roles and to track changes in conception as they participated in typical speech and language therapy intervention provided by services in the UK. The study posed the following research questions: 1. What are parents’ conceptions of their own and SLTs’ [speech and language therapists'] roles in speech and language therapy intervention for their pre-school children? 2. How do parents’ conceptions of these roles change during speech and language therapy intervention?" | Family members (parents) | Qualitative longitudinal research | Individual interviews | 30 weeks | 14 | 27 interviews |
| Davies, S., Salmon, P., & Young, B. (2017). When trust is threatened: Qualitative study of parents' perspectives on problematic clinical relationships in child cancer care. Psycho-Oncology, 26(9), 1301-1306. | UK | "So we took an inductive approach, analysing qualitative interviews with parents who described pervasive difficulties in their relationships with clinicians to understand their needs and identify ways of helping them." | Family members (parents who described pervasive difficulties in their relationships with clinicians in childhood cancer) | Qualitative longitudinal research | Individual interviews | 12 months | 20 | 50 interviews |
| Davis, E. B., Kimball, C. N., Aten, J. D., Andrews, B., Van Tongeren, D. R., Hook, J. N., Davis, D. E., Granqvist, P., & Park, C. L. (2019). Religious meaning making and attachment in a disaster context: A longitudinal qualitative study of flood survivors. Journal of Positive Psychology, 14(5), 659-671. | US | "In the current study, we develop a grounded theory of how people draw on their religion and their religious attachment (perceived relationship with God) to make meaning of their disaster experiences. More specifically, we employed a longitudinal qualitative design to examine how post-disaster religious meaning making and attachment unfold over time and to compare these processes in the acute and intermediate wake of a disaster (e.g. at 4 weeks [Time 1, T1] and 6 months post-disaster [Time 2, T2]) The specific research questions we sought to explore were: (1) How do disaster survivors engage in religious meaning making after a disaster? (2) What role does religious attachment play in this process of religious meaning making? (3) Does this religious meaning making process differ across disaster phases?" | People in the community (disaster survivors) | Grounded theory | Individual interviews | 6 months | 36 | 65 interviews |
| Davis, E. B., Kimball, C. N., Aten, J. D., Hamilton, C., Andrews, B., Lemke, A., Hook, J. R., Captari, L., Granqvist, P., Hook, J. N., Davis, D. E., Van Tongeren, D. R., Cattrell, E. L., Cuthbert, A. D., Chung, J. (2019). Faith in the wake of disaster: A longitudinal qualitative study of religious attachment following a catastrophic flood. Psychological Trauma: Theory, Research, Practice & Policy, 11(6), 578-587. | US | "...we anticipated this natural disaster would have some type of effect on survivors’ religious attachment (i.e., perceived relationship with God). We chose a longitudinal design in order to permit an examination of these effects over time and a comparison of such effects in the acute versus intermediate wake of the disaster. Research Question 1: In the wake of a disaster, what types of religious-attachment language do theistic survivors use to describe their pre-disaster and post-disaster religious attachment? Research Question 2: After a disaster, what types of differences are there in the religious-attachment language that theistic disaster survivors use to describe their religious attachment, based on the type of disaster exposure they have experienced (e.g., direct vs. indirect exposure) or based on their previous exposure to a catastrophic natural disaster? Research Question 3: How do theistic survivors describe the quality and characteristics of their post-disaster religious attachment (perceived relationship with God)? What are the themes that emerge as survivors discuss various aspects of their post-disaster religious attachment?" | People in the community (disaster survivors) | Qualitative longitudinal research | Individual interviews | 6 months | 36 | 65 interviews |
| Day, M. R., Thompson, A. R., Poulter, D. R., Stride, C. B., & Rowe, R. (2018). Why do drivers become safer over the first three months of driving? A longitudinal qualitative study. Accident; Analysis And Prevention, 117, 225-231. | UK | "We adopted an innovative longitudinal qualitative design, with thirteen newly qualified drivers completing a total of 36 semi-structured interviews, one, two and three months after acquiring a full UK driving license. The interviews probed high-risk factors for new drivers, as well as allowing space for generating novel road safety issues." (from abstract) | People in the community (new drivers) | qualitative longitudinal research | Individual interviews | 3 months | 13 | 36 interviews |
| De Clercq, M., Roland, N., Brunelle, M., Galand, B., & Frenay, M. (2018). The delicate balance to adjustment: A qualitative approach of student's transition to the first year at university. Psychologica Belgica, 58(1), 67-90. | Belgia | "More precisely, two main objectives underlie this approach: (1) to identify the key determinants and events of adjustment regarding the student’s experience; (2) to understand how these determinants and events interact in the adjustment process across the first year." | Students (students in biology) | qualitative longitudinal research | Individual interviews | 12 months | 17 | Unclear |
| DeGuzman, P. B., Colliton, K., Nail, C. J., & Keim-Malpass, J. (2017). Survivorship care plans: Rural, low-income breast cancer survivor perspectives. Clinical Journal of Oncology Nursing, 21(6), 692-698. | US | "This pilot study explores post-treatment survivorship care planning execution, perception, and needs among rural, low-income cancer survivors." (from abstract) | Patients (rural, low-income cancer survivors) | Qualitative longitudinal research | Individual interviews | 6 months | 7 | 15 interviews |
| den Herder-van der Eerden, M., Hasselaar, J., Payne, S., Varey, S., Schwabe, S., Radbruch, L., Van Beek, K., Menten, J., Busa, C., Csikos, A., Vissers, K., & Groot, M. (2017). How continuity of care is experienced within the context of integrated palliative care: A qualitative study with patients and family caregivers in five European countries. Palliative Medicine, 31(10), 946-955. | Belgium, Germany, Hungary, the Netherlands and UK | "Therefore, this article examines how relational, informational and management continuity of care are experienced by patients with advanced cancer, chronic obstructive pulmonary disease (COPD) or heart failure and family caregivers receiving care from integrated palliative care initiatives in five European countries." | Several: Patients and family members (patients with advanced cancer, chronic obstructive pulmonary disease or heart failure and their family caregivers) | Qualitative longitudinal research | Individual interviews | 3 months | 244 | 388 interviews |
| Denney-Koelsch, E. M., Côté-Arsenault, D., & Jenkins Hall, W. (2018). Feeling cared for versus experiencing added burden: Parents' interactions with health-care providers in pregnancy with a lethal fetal diagnosis. Illness, Crisis & Loss, 26(4), 293-315. | US | "The focus of this report is parents’ perspectives on health-care provider interactions from the time of prenatal diagnosis through postpartum." | Family members (parents pregnant with a fetus with a lethal fetal diagnosis) | phenomenology | Individual interviews and joint interviews | Unclear, "The goal was to interview participants individually and jointly twice during pregnancy and twice after the baby’s birth and subsequent death." | 30 | Unclear |
| Donnellan, W. J., Bennett, K. M., & Soulsby, L. K. (2018). How does carer resilience change over time and care status? A qualitative longitudinal study. Aging & Mental Health, 1-7. | UK | "Based on our original sample of 20 spousal dementia carers, the current study uses qualitative longitudinal methods to follow the care status transitions of 13 current and former spousal dementia carers, including: continuing home carers; former carers (institutionalised); and former carers (widowed). We address the following research objectives: i. To examine trajectories of resilience in spousal dementia carers over time and across care status, and ii. To identify which assets and resources from the resilience framework (Windle & Bennett, 2011) are associated with resilience and care status transitions." | Family members (care givers to spous with dementia) | Qualitative longitudinal research | individual interviews | 18 to 36 months | 23 | 36 interviews |
| Dowell, A., Stubbe, M., Macdonald, L., Tester, R., Gray, L., Vernall, S., Kenealy, T., Sheridan, N., Docherty, B., Hall, D.-H., Raphael, D., & Dew, K. (2018). A longitudinal study of interactions between health professionals and people with newly diagnosed diabetes. Annals of Family Medicine, 16(1), 37-44. | New Zealand | "We undertook video observation of a cohort of patients with newly diagnosed diabetes to gain an in-depth picture of communication and miscommunication processes within a primary health care team over time." | Patients (patients with newly diagnosed diabetes) | not described | Observations (video recordning) | 6 months | 32 | Unclear |
| Draaisma, A., Meijers, F., & Kuijpers, M. (2017). Towards a strong career learning environment: Results from a Dutch longitudinal study. British Journal of Guidance & Counselling, 45(2), 165-177. | the Netherlands | "This longitudinal study is designed to gain theoretical and practical insight into the influence of the project ‘COG/SVE’ on the creation of a strong career learning environment. Moreover, this article describes how teachers and project managers of these schools perceive the developments in their own learning environment and the learning environment of their students, since the start of the project. Three research questions will therefore be investigated: (1) How and to what extent does the project ‘COG/SVE’ accomplish strong career learning environments that are dialogical, practice- and inquiry-based? (2) To what extent does the project stimulate collective learning of the teachers? (3) To what extent is transformational leadership present, needed to promote the development of a strong career learning environment?" | Several: Teachers and project managers | Qualitative longitudinal research | Individual interviews | 5 to 6 months | 83 | 136 interviews |
| Drake, E. K., & Urquhart, R. (2019). "Figure out what it is you love to do and live the life you love": The experiences of young adults returning to work after primary cancer treatment. Journal of Adolescent & Young Adult Oncology, 8(3), 368-372. | Canada | "The aim of this study was to explore younger adults (YA) cancer survivors’ perspectives on and experiences with return to work (RTW) following primary cancer treatment." | Patients (younger adults cancer survivors) | Phenomenology and qualitative longitudinal research | Individual interviews | 9 months | 5 | 14 interviews |
| Dubé, T., Schinke, R., & Strasser, R. (2019). It takes a community to train a future physician: Social support experienced by medical students during a community-engaged longitudinal integrated clerkship. Canadian Medical Education Journal, 10(3), e5-e16. | Canada | "The purpose of our study was to learn which sources of social support students sought out during a rural-based LIC [longitudinal integrated clerkship]. Therefore, our purpose was to answer the following research question: Which, if any, sources of social support do students experience during a LIC in the context of rural family practice?" | Students (medical students year 3) | social constructivistic | Individual interviews | 9 months | 12 | Unclear, three time-points planed but no information regarding drop-outs. |
| Due‐Christensen, M., Willaing, I., Ismail, K., & Forbes, A. (2019). Learning about type 1 diabetes and learning to live with it when diagnosed in adulthood: Two distinct but inter‐related psychological processes of adaptation a qualitative longitudinal study. Diabetic Medicine, 36(6), 742-752. | UK and Denmark | "… explored the adaptation processes that adults experience after their diagnosis of Type 1 diabetes in order to elicit areas for supportive intervention to enhance the psychosocial well-being of adults during this phase of life with Type 1 diabetes." | Patients (adults diagnosed with type 1 diabetes within the last 3 years) | Narrative research & qualitative longitudinal research | Individual interviews | 6 months | 30 | 58 interviews |
| Dunivan, G. C., McGuire, B. L., Rishel Brakey, H. A., Komesu, Y. M., Rogers, R. G., & Sussman, A. L. (2019). A longitudinal qualitative evaluation of patient perspectives of adverse events after pelvic reconstructive surgery. International Urogynecology Journal. 30, 2023–2028. | US | "… to longitudinally examine how patients view surgical AEs [adverse events] overtime and explore how such perspectives may change from the preoperative period to 6 months after surgery." | Patients (women planning pelvic floor disorder surgery) | mixed methods | Individual interviews | approximatley 11 months | 20 | 60 interviews |
| Dury, S. (2018). Dynamics in motivations and reasons to quit in a Care Bank: A qualitative study in Belgium. European Journal of Ageing, 15(4), 407-416. | Belgium | "Given the previously established importance of examining (changes in) motives for and reasons to quit time bank volunteering, we investigated what motivates people to start and what encourages or prevents them from continuing, and whether these motivations change throughout the project. In addition, we examined the moderating effects of individual and contextual factors that may play a role in the changes in motives and reasons to quit volunteering with a time bank. To achieve our aim, we posed the following research questions: 1a. What motivates volunteers to start participating in the Neighborhood Pension project? 1b. How do these motives change over time? 1c. Which moderating factors change participants’ motives over time? 2a. What reasons to quit do volunteers express at the start of their participation in the project? 2b. How do these reasons change over time? 2c. Which moderating factors change participants’ reasons over time?" | People in the community (volunteers) | qualitative longitudinal research | focus group interviews, and questionnaire | 11 months | 13 | 4 focus group interviews |
| Eaton Russell, C., Widger, K., Beaune, L., Neville, A., Cadell, S., Steele, R., Rapoport, A., Rugg, M., & Barrera, M. (2018). Siblings’ voices: A prospective investigation of experiences with a dying child. Death Studies, 42(3), 184-194. | Canada | "study aimed to fill some of the research gaps by examining healthy siblings’ perspectives of their experiences when a brother or sister was dying. Specifically, we investigated: (a) siblings’ involvement with the dying child and (b) the impact of involvement on siblings’ grief, growth, and coping and their interpersonal world within and outside the family." | Family members (siblings to dying children) | interpretative description & qualitative longitudinal research | Individual interviews | 12 weeks | 10 | 19 interviews |
| Eg, M., Frederiksen, K., Vamosi, M., & Lorentzen, V. (2017). How family interactions about lifestyle changes affect adolescents' possibilities for maintaining weight loss after a weight-loss intervention: A longitudinal qualitative interview study. Journal of Advanced Nursing (John Wiley & Sons, Inc.), 73(8), 1924-1936. | Denmark | "Thus, the purpose of this study is to examine how the family interactions related to lifestyle changes influence the adolescent’s potential for maintaining weight loss after participating in a weight-loss treatment programme." | Family members (adolescents and the parents) | hermeneutic tradition & qualitative longitudinal research | Individual interviews | 5 years | 38 | 79 interviews |
| Elberg Dengsø, K., Tjørnhøj-Thomsen, T., Oksbjerg Dalton, S., Christensen, B. M., Hillingsø, J., & Thomsen, T. (2019). It's all about the CA-19-9. A longitudinal qualitative study of patients' experiences and perspectives on follow-up after curative surgery for cancer in the pancreas, duodenum or bile-duct. Acta Oncologica, 58(5), 642-649. | Denmark | "In this study, we aimed to explore patients’ experiences of follow-up to get a sense of their perspectives on the rehabilitative scope of the current follow-up within the first year after surgery and adjuvant chemotherapy with curative intent." | Patients (patients attending current follow-up after treatment for cancer in the pancreas, duodenum or bileduct) | longitudinal qualitative research | Individual interviews | 9 months | 12 | 36 interviews |
| Elliott, K., & McVicar, A. (2018). The impact of prolonged disorders of consciousness on the occupational life of family members. Neuropsychological Rehabilitation, 28(8), 1375-1391. | UK | "To explore the process of occupational adaptation within a group of primary caregivers." | Family members (partners/primary caregivers) | mixed methods | Individual interviews, time diaries, and questionnaires | 6 months | 6 | Unclear |
| Elliott, M. C., Shuey, E. A., Zaika, N., Mims, L., & Leventhal, T. (2017). Finding home: A qualitative approach to understanding adolescent mothers' housing instability. American Journal of Community Psychology, 60(1), 55-65. | US | "The aim of this study was to use a qualitative approach to explore the unique experiences of low-income Latina adolescent mothers who depended on their families of origin for housing and parenting support and had unstable living conditions (into another doubling up situation—a horizontal move—or into independent housing—a vertical move)." | People in the community (low-income Latina adolescent mothers) | Ethnography | Individual interviews | 3 years | 15 | Unclear |
| Eriksson, C., Erikson, A., Tham, K., & Guidetti, S. (2017). Occupational therapists experiences of implementing a new complex intervention in collaboration with researchers: A qualitative longitudinal study. Scandinavian Journal of Occupational Therapy, 24(2), 116-125. | Sweden | "The aim of this study was to identify and describe the process of how OTs [occupational therapists] in collaboration with researchers implemented a client-centred ADL [activities of daily living] intervention for persons with stroke." | Health care providers (occupational therapists) | Qualitative longitudinal research | Focus group interviews | 10 months | 33 | 15 focus group interviews |
| Essery, R., Kirby, S., Geraghty, A. W. A., & Yardley, L. (2017). Older adults' experiences of internet-based vestibular rehabilitation for dizziness: A longitudinal study. Psychology & Health, 32(11), 1327-1347. | UK | "...the study aimed to determine the acceptability and accessibility of an internet-based intervention for delivering VR [viritual reality] to older adults with dizziness, and to provide insight into how they engage with, and utilise, such an intervention. To achieve this, the main objectives of the study were to gain a greater understanding of: how older adults experience internet-based VR, including their perceptions of its impact upon their symptoms; their perceptions of what may help or hinder their engagement with a self-directed VR programme; and how their experiences change over the intervention period." | Patients (older adults with dizziness) | Phenomenology and qualitative longitudinal study | Individual interviews | 6 weeks | 18 | 47 interviews |
| Evans, B. C., Coon, D. W., Belyea, M. J., & Ume, E. (2017). Collective care: Multiple caregivers and multiple care recipients in mexican american families. Journal Of Transcultural Nursing: Official Journal Of The Transcultural Nursing Society, 28(4), 398-407. | US | "We will describe the understudied phenomenon of multiple caregiving in MA [Mexican American] families, asking, “How do MA families adapt to the informal care needs of more than one older family member?”" | Family members (Mexican American families) | Case study | Interviews (unclear if individual or joint), and standardized instruments | 15 months | 47 | Unclear |
| Fadyl, J. K., Theadom, A., Channon, A., & McPherson, K. M. (2019). Recovery and adaptation after traumatic brain injury in New Zealand: Longitudinal qualitative findings over the first two years. Neuropsychological Rehabilitation, 29(7), 1095-1112. | New Zealand | "To investigate the subjective experiences of recovery and adaptation over the first two years after having a TBI [traumatic brain injury] from the perspective of people with the injury and the family and/or friends most closely involved in their recovery (who we termed “significant others”). The specific research questions focused on what helped or hindered recovery and adaptation over time following TBI. " | Several: Patients and family members (people with traumatic brain injury, their family and /or friends closely involved in the recovery) | longitudinal qualitative research | Individual interviews and joint interviews | about 2 years | 62 | 152 interviews |
| Farr, M. (2018). Power dynamics and collaborative mechanisms in co-production and co-design processes. Critical Social Policy, 38(4), 623-644. | UK | "describe empirical examples of co-production and co-design processes within public services" | Several: Patients and health-care providers (Breast cancer patients and staff, e.g., policy managers, senior managers, service managers, project co-ordinators and front-line staff) | Case study | Observations, feedback sheets from co-design events, organisational documents and reports, individual interviews and focus group interviews | 19 to 22 month (differs between cases) | 71 | 43 interviews, 3 focus group interviews, 71 feedback sheets |
| Felice, J. P., Geraghty, S. R., Quaglieri, C. W., Yamada, R., Wong, A. J., & Rasmussen, K. M. (2017). 'Breastfeeding' without baby: A longitudinal, qualitative investigation of how mothers perceive, feel about, and practice human milk expression. Maternal & Child Nutrition, 13(3), e12426. | US | "Here, we describe results related to the mothers' perspective: their attitudes and perceptions of, strategies for, and experiences with pumping, including their motivations to pump, how they incorporated pumping into infant feeding practices and other obligations, and how their attitudes, perceptions, practices, and experiences changed over time." | People in the community (mothers pumping breast milk) | Qualitative longitudinal research | Individual interviews and observations | Up to one year | 20 | 108 interviews |
| Fletcher, A. C., & Blair, B. L. (2018). Youth disclosure about friendships across the transition to middle school. Journal of Early Adolescence, 38(5), 606-628. | US | "To considered youth disclosure to parents about friends as well as parents’ responses to such disclosure across the transition to middle school. Research Question 1: How does disclosure to parents about friends change or remain stable across the transition to adolescence? Given that adolescence is a time of increased desire for independence from parents, we hypothesize that the most typical pattern of disclosure over time will involve adolescents disclosing less to parents as they transition into adolescence. Research Question 2: What are the emotional components of adolescent disclosure to parents? Based on research indicating that levels of disclosure vary based on adolescent involvement in problem behaviors and perceptions regarding how parents will react to disclosure, we hypothesize that youth will perceive both positive and negative components of disclosure, depending in part on the nature of the information being disclosed and in part on the manner in which parents react to disclosure. Research Question 3: How do youth perceptions regarding their parents and their parents’ reactions to disclosure shape stability and change in disclosure? Consistent with quantitative research findings, we hypothesize that good parenting, encouragement by parents to share information, feelings of trust and security in relationships with parents, and perceptions that parents respect adolescent desires for autonomy will all shape adolescent decisions regarding disclosure." | Students (children grade 5, 6, 7) | mixed methods & qualitative longitudinal research | Individual interviews | about 3 years | 20 | 60 interviews |
| Forslund, A.-S., Jansson, J.-H., Lundblad, D., & Söderberg, S. (2017). A second chance at life: People's lived experiences of surviving out-of-hospital cardiac arrest. Scandinavian Journal of Caring Sciences, 31(4), 878-886. | Sweden | "The aim of this study was to elucidate meanings of people’s lived experiences and changes in daily life during their first year after surviving OHCA [out-of-hospital cardiac arrest]." | Patients (people surviving out-of-hospital cardiac arrest) | phenomenological hermeneutic interpretation & qualitative longitudinal research | Individual interviews | 6 months | 11 | unclear, probably 22 interviews |
| Foster, K., Mitchell, R., Van, C., Young, A., McCloughen, A., & Curtis, K. (2019). Resilient, recovering, distressed: A longitudinal qualitative study of parent psychosocial trajectories following child critical injury. Injury, 50, 1605-1611. | Australia | "This study aimed to explore parent experiences and psychosocial support needs and identify parent psychosocial trajectories in the 12 months following child critical injury. Research questions were: What are the psychosocial trajectories for parents of critically injured children in the 12 months following injury? What factors facilitate or hinder the psychosocial trajectories of parents of critically injured children in the 12 months following injury?" | Family members (parents to a critically injured child) | longitudinal qualitative research | Individual interviews | 12 months | 27 | 81 interviews |
| Freytag, J., Jiang, Z. J., Giordano, T. P., Westbrook, R. A., McCurdy, S. A., Njue-Marendes, S., & Dang, B. N. (2019). What patient involvement means to new patients at two HIV clinics: A longitudinal, qualitative study. Patient Education & Counseling, 102(8), 1535-1540. | US | "...we aim to: 1) present definitions of patient involvement from the perspectives of patients seeing a new provider, 2) examine physician behaviors that patients identify as cultivating their involvement, and 3) identify some of the ways new patient expectations of involvement change after their first visit with a new provider." | Patients (adults with HIV) | Qualitative longitudinal research | Individual interviews | 6 to 12 months | 56 | 138 interviews |
| Froh, E. B., Deatrick, J. A., Curley, M. A. Q., & Spatz, D. L. (2017). Mothers of infants with congenital diaphragmatic hernia describe "breastfeeding" in the neonatal intensive care unit: "As long as it's my milk, i'm happy". Journal Of Human Lactation, 33(3), 524-532. | US | "This article focuses explicitly on the mothers’ descriptions of what breastfeeding truly means in the context of a diagnosis of CDH [Congenital Diaphragmatic Hernia] for their infants and the course of care in the NICU setting." | Family members (mothers with an infant with a diagnosis of Congenital Diaphragmatic Hernia) | Qualitative longitudinal research | Individual interviews | Unclear, during time at hospital | 11 | Unclear |
| Frost, J., Wingham, J., Britten, N., Greaves, C., Abraham, C., Warren, F. C., Jolly, K., Doherty, P. J., Miles, J., Singh, S. J., Paul, K., Rod Taylor, Dalal, H. (2019). Home-based rehabilitation for heart failure with reduced ejection fraction: Mixed methods process evaluation of the REACH-HF multicentre randomised controlled trial. BMJ Open, 9(8), e026039-e026039. | UK | "The work reported here constituted part of the REACH-HF process evaluation that assessed intervention fidelity, patients’ and caregivers’ experiences of trial participation and sought to identify change processes that may be responsible for change in HRQoL (the trial primary outcome)." | Several: Patients, family members and health-care providers (people with heart failure taking part in an patient education program, caregivers and facilitators in the program) | Case study and mixed methods | Individual interviews, fieldnotes, and recorded consultations | 12 months | 19 | Unclear. |
| Fu, F., Chen, L., Sha, W., Chan, C. L. W., Chow, A. Y. M., & Lou, V. W. Q. (2018 published online). Mothers' grief experiences of losing their only child in the 2008 sichuan earthquake: A qualitative longitudinal study. Omega – Journal of Death and Dying, 2020, 81(1) 3–17. | China | "Thus, this study not only explores bereaved mothers’ longitudinal grief experiences since the earthquake but also examines their experiences within the Chinese sociocultural contexts." | Family members (bereaved mothers’) | phenomenological & qualitative longitudinal research | Individual interviews | 2 years | 6 | 24 interviews |
| Gallagher, K., Partridge, C., Tran, H. T., Lubran, S., & Macrae, D. (2017). Nursing & parental perceptions of neonatal care in Central Vietnam: A longitudinal qualitative study. BMC Pediatrics, 17(1), 161-161. | Vietnam | "The aim of this study was to explore changes in the perceptions and attitudes of nurses and parents towards their experiences in the neonatal unit following a neonatal nursing education intervention in a single neonatal unit in central Vietnam. | Several: Family members and health-care providers (parents of infants admitted to the neonatal unit and neonatal nurses who had undertaken the education intervention) | Qualitative longitudinal research | Individual interviews | 18 months | 83 | 115 interveiws |
| Gammons, R. W., Carroll, A. J., & Carpenter, L. I. (2018). "I never knew I could be a teacher": A student-centered MLIS fellowship for future teacher-librarians. Portal: Libraries & the Academy, 18(2), 331-362. | US | "...results from a mixed methods and longitudinal study identifying the successful components of RTF [Research and Teaching Fellowship] and charting the development of teacher efficacy and identity among participants" (from abstract). "Our research had two directives: (1) to identify successful components of RTF and better understand how these elements contributed to the growth and development of participants; and (2) to chart the development of teacher efficacy and teacher identity among participants." (from background] | Several: Students and teachers (students seeking a master’s of library and information science, their teachers, mentors and administratve staff) | grounded theory and mixed methods | Indivudual interviews, focus group interviews, written reflections, and questionnaires | 18 months | 17 | Unclear |
| Garbett, K., Harcourt, D., & Buchanan, H. (2017). Using online blogs to explore positive outcomes after burn injuries. Journal of Health Psychology, 22(13), 1755-1766. | UK, US and Australia | "This study aims to build on current research by qualitatively exploring the positive outcomes that may be present following a burn injury since previous literature offers limited knowledge into the specific positive aspects that may arise in this growing population. Acknowledging that they can exist, and exploring specifically what they are, is an important step in guiding burn care practice and may help to challenge assumptions about the ubiquity of negative impacts following burn injuries." | Patients (burn survivors) | not described | blog texts | Unclear | 10 | Unclear |
| Garner, C. D., McKenzie, S. A., Devine, C. M., Thornburg, L. L., & Rasmussen, K. M. (2017). Obese women experience multiple challenges with breastfeeding that are either unique or exacerbated by their obesity: Discoveries from a longitudinal, qualitative study. Maternal & Child Nutrition, 13(3), e12344. | US | "Our aim was to understand obese women’s experiences and perceptions longitudinally, with a normal-weight comparison group, beginning in late pregnancy and continuing through 3months post-partum, to identify key experiences and barriers that are unique to or more common among obese women." | Patients (obese women’s with an infant) | Qualitative longitudinal research | individual interviews | 6 months | 22 | Unclear |
| Garrett, S. B., Abramson, C. M., Rendle, K. A., & Dohan, D. (2019). Approaches to decision-making among late-stage melanoma patients: A multifactorial investigation. Supportive Care in Cancer, 27(3), 1059-1070. | US | "...to characterize late-stage melanoma patients holistically as treatment decision-makers." | Patients (late-stage melanoma patients) | Ethnography | Observations, and individual interviews | Up to 2 years | 13 | Unclear. |
| Gaskin, K. L., Wray, J., & Barron, D. J. (2018). Acceptability of a parental early warning tool for parents of infants with complex congenital heart disease: A qualitative feasibility study. Archives of Disease In Childhood, 103(9), 880-886. | UK | "To explore the acceptability and feasibility of a parental early warning tool, called the Congenital Heart Assessment Tool (CHAT), for parents going home with their infant between first and second stage of surgery for complex congenital heart disease." | Family members (parents of infants with complex congenital heart disease needing sugery) | mixed methods | Individual interviews and joint interviews | Unclear, a few months, (time point 3 was 8 weeks after time point 1, time point 4 unclear) | 12 | 22 interviewes |
| Gelpí-Acosta, C., Guarino, H., Benoit, E., Deren, S., Pouget, E. R., & Rodríguez, A. (2019). Injection risk norms and practices among migrant Puerto Rican people who inject drugs in New York City: The limits of acculturation theory. International Journal of Drug Policy, 69, 60-69. | US | "This study identifies the P.R. [Puerto Rico] native norms supporting the continued injection risk behavior of migrant Puerto Rican PWID [people who inject drugs] in NYC [New Yourk City] to inform a culturally appropriate risk-reduction intervention." (from abstract) | People in the community (migrant Puerto Rican adults who inject drugs) | Grounded theory & qualitative longitudinal research | Individual interviews | 12 months | 40 | 74 interviews (including base-line and exit interviews), and monthly follow-ups with 35 participants. |
| Gilliland, S. (2017). Physical therapist students' development of diagnostic reasoning: A longitudinal study. Journal of Physical Therapy Education, 31(1), 31-48. | US | "...this qualitative study examined how PT [physical therapy] students’ diagnostic reasoning processes develop through their coursework and clinical experiences." | Students (physio therapy students) | not described | Individual interviews and field notes | about 18 months | 6 | Unclear, probably 18 interviews |
| Godino, L., Jackson, L., Turchetti, D., Hennessy, C., & Skirton, H. (2018). Decision making and experiences of young adults undergoing presymptomatic genetic testing for familial cancer: A longitudinal grounded theory study. European Journal Of Human Genetics, 26(1), 44-53. | Italy | "...explore the psychosocial implications of pre-symptomatic testing for hereditary cancer in Italian young adults aged 18–30 years" | Patients (new young consult and making an appointment for the cancergenetics clinic) | Grounded theory | Individual interviews | about 7 months | 15 | 42 inteviews |
| Goedken, C. C., Moeckli, J., Cram, P. M., & Reisinger, H. S. (2017). Introduction of Tele-ICU in rural hospitals: Changing organisational culture to harness benefits. Intensive & Critical Care Nursing, 40, 51-56. | US | "Our study expands this research through a qualitative investigation of rural ICU [intensive care unit] staff perceptions of Tele-ICU. We conducted a longitudinal qualitative study of three rural ICUs located in the upper Midwest of the United States to illuminate ways in which Tele-ICU can best serve rural facilities. For our study, clinicians and ICU administrators in rural ICUs were asked to discuss perceptions of Tele-ICU on care processes, practices and perceived need before and after implementation." | Health care providers (rural ICU staff) | Qualitative longitudinal research | Individual interviews and focus group interviews | 12 months | 24 | Unclear |
| González, M. G., Kelly, K. N., Dozier, A. M., Fleming, F., Monson, J. R. T., Becerra, A. Z., Aquina, C. T., Probst, C, P., Hensley B. J., Sevdalis, N., & Noyes, K. (2017). Patient perspectives on transitions of surgical care: Examining the complexities and interdependencies of care. Qualitative Health Research, 27(12), 1856-1869. | US | "The goal of this study is to examine the surgical care pathway from the patient’s point of view to identify experiences and events that influence patient outcomes (e.g., a patient’s satisfaction, anxiety, and discharge readiness) in an effort to improve care transitions and reduce patient burden." | Patients (adult patients undergoing colon or rectal resection) | Grounded theory | Individual interviews | one month | 20 | 61 interviews |
| Gordon, L., Jindal-Snape, D., Morrison, J., Muldoon, J., Needham, G., Siebert, S., & Rees, C. (2017). Multiple and multidimensional transitions from trainee to trained doctor: A qualitative longitudinal study in the UK. BMJ Open, 7(11), e018583-e018583. | UK | "...to address the research gaps identified above by including trainees working in a range of specialties and contexts, and through its longitudinal study design, follows participants as they move from trainee to trained doctor roles. Our research questions are: (1) What MMTs [multiple and multidimensional transitions] are experienced as participants move from trainee to trained doctor? (2) What facilitates and hinders doctors’ successful transition experiences? (3) What is the impact of MMTs on trained doctors and their significant others?" | Health care providers (trainee doctors) | Narrative research & qualitative longitudinal research | Individual interviews, and audio-diaries | 12 months | 20 | 38 interviews, 18 participants contributed with diary entries |
| Graham-Wisener, L., Hanna, J., Collins, L., & Dempster, M. (2019). Psychological adjustment in patients post-curative treatment for oesophageal cancer: A longitudinal interview study. Psychology & Health, 34(8), 901-921. | Ireland | "In contrast to earlier research, this study will involve a sample of survivors for which less time has elapsed since treatment-end and will utilise a longitudinal qualitative design, recommended to suggest mechanisms involved in change during key transition periods by exploring how and why experiences change over time." | Patients (oesophageal cancer patients) | Phenomenology & qualitative longitudinal research | Individual interviews | 6 months | 6 | 10 interviews |
| Granbom, M., Taei, A., & Ekstam, L. (2017). Cohabitants' perspective on housing adaptations: A piece of the puzzle. Scandinavian Journal of Caring Sciences, 31(4), 805-813. | Sweden | "The aim of this study was therefore to describe the cohabitants´ expectations and experiences of how a housing adaptation, intended for the partner, impacted on everyday life." | Family members (cohabitants of persons applying for housing adaption) | Grounded theory & qualitative longitudinal research | Individual interviews | avarage of 7 months | 9 | 18 interviews |
| Graney, B. A., Wamboldt, F. S., Baird, S., Churney, T., Fier, K., Korn, M., McCormick, M., Vierzba, T., & Swigris, J. J. (2017). Looking ahead and behind at supplemental oxygen: A qualitative study of patients with pulmonary fibrosis. Heart & Lung, 46(5), 387-393. | US | "In this study, we sought to better understand how patients with PF [pulmonary fibrosis] view and experience O2 [oxygene]- its benefits and challenges - at various stages of their illness." "Interviews were designed to gain appreciation for patients’ understanding of the process of when, why and how O2 was prescribed, and to examine their changing views of the benefits and challenges of O2 over time." | Patients (patients with pulmonary fibrosis) | mixed methods | Individual interviews , observations, questionnaires | 10 to 13 months | 5 | 20 interviews |
| Grossman, J. M., Jenkins, L. J., & Richer, A. M., (2018). Parents' perspectives on family sexuality communication from middle school to high school. International Journal Of Environmental Research And Public Health, 15(1). | US | "The current study provides a unique longitudinal examination of parents’ perceptions of continuity and change in teen-parent communication from middle school to high school. The knowledge gained from this study will guide our understanding of how parents do or do not adapt their approaches to sexuality communication to teens’ changing development and sexuality. It also explores the role of teen gender in shaping parents’ approaches to talk with teens about sex and relationships and the content of these conversations." | Family members (parents of adolescents from three schools that participated in an evaluation of Get Real) | qualitative longitudinal research | Individual interviews | about 3 years | 29 | 52 interviews |
| Grylka-Baeschlin, S., Meyer, T., Lengler, L., van Teijlingen, E., Pehlke-Milde, J., & Gross, M. M. (2019). Postnatal quality of life — A content analysis of qualitative results to the Mother-Generated Index. Women & Birth, 32(2), e229-e237. | Germany and Switzerland | "The aims of this paper were therefore to investigate: (a) details and particularities of the are as of life affected after child birth and thus to identify specific domains and subdomains defining postnatal QoL; (b) changes in the importance of domains specifying QoL within the first weeks postpartum; and (c) the potential role of cultural differences with regard to the content of QoL definitions." | People in the community (women in post natal care) | not described | Open ended questions in questionnairs | 6 weeks | 124 | Data from 221 questionnairs |
| Gulbas, L. E., Guz, S., Hausmann-Stabile, C., Szlyk, H. S., & Zayas, L. H. (2019). Trajectories of well-being among latina adolescents who attempt suicide: A longitudinal qualitative analysis. Qualitative Health Research, 29(12), 1766-1780. | US | "Research Question 1: How do trajectories of well-being vary among Latina teens after a suicide attempt? Research Question 2: What risk and protective factors might contribute to different trajectories?" | Patients (Latina teens after a suicide attempt) | longitudinal qualitative research | Individual interviews | about one year | 17 | 34 interviews |
| Guldager, R., Willis, K., Larsen, K., & Poulsen, I. (2019). Relatives' strategies in subacute brain injury rehabilitation: The warrior, the observer and the hesitant. Journal Of Clinical Nursing, 28(1), 289-299. | Denmark | "The aim of this study was to undertake theoretical‐empirical analysis of relatives’ strategies and practices in the rehabilitation process as evident in meetings with providers. We explored the experience of the rehabilitation process from the perspectives of relatives of patients with a traumatic brain injury [TBI]. Our research question was 'what kind of strategies do relatives of patients with a TBI apply and use in the rehabilitation process?' " | Family members (relatives of patients with a TBI) | qualitative longitudinal research | Observations and individual interviews | Unclear, over a few weeks | 11 | 22 meetings were observed |
| Hamblin, K. (2017). Telecare, obtrusiveness, acceptance and use: An empirical exploration. British Journal of Occupational Therapy, 80(2), 132-138. | UK | "...to examine the daily experiences of telecare. The research questions addressed in this paper are therefore: (1) whether Hensel et al.’s (2006) obtrusiveness framework is applicable empirically to the English context; and (2) what is the impact of the dimensions of obtrusiveness on the acceptance and use of telecare?" | Patients (people over 65 years, had memory problems and/or susceptibility to falls, were living in the communit, and were either ‘new’ or established telecare users) | ethnography & qualitative longitudinal research | Individual interviews | 11 to 24 months (differed between the two data collection sites) | 25 | 43 interviews |
| Hanrahan, F., & Banerjee, R. (2017). ‘It makes me feel alive’: The socio-motivational impact of drama and theatre on marginalised young people. Emotional & Behavioural Difficulties, 22(1), 35-49. | UK | "We aimed to explore the participants’ experiences of long-term involvement in drama and theatre work from an idiographic, phenomenological perspective, and to consider the young people’s narratives in relation to the psychological mechanisms identified by our model of disaffection/engagement." | People in the community (young people conducting a theatre project) | Phenomenology & qualitative longitudinal research | Individual interviews | 22 months | 4 | 12 interviews |
| Hansen, C. A., Abrahamsen, B., Konradsen, H., & Pedersen, B. D. (2017). Women's lived experiences of learning to live with osteoporosis: A longitudinal qualitative study. BMC Women's Health, 17(1), 17-17. | Denmark | "The objective was to explore what characterizes women’s experiences of living with osteoporosis in the first year after diagnosis, when patients are prescribed anti-osteoporotic treatment, without experiencing an osteoporotic fracture." | Patients (women 65 years or older who attended DXA scan and diagnosed with osteoporosis) | Phenomenology & qualitative longitudinal research | Individual interviews | one year | 15 | 42 interviews |
| Hansen, F., Berntsen, G. K. R., & Salamonsen, A. (2018). "What matters to you?" A longitudinal qualitative study of Norwegian patients' perspectives on their pathways with colorectal cancer. International Journal of Qualitative Studies on Health And Well-Being, 13(1), 1548240-1548240. | Norway | "We aim to offer rich descriptions of the participants’ lives lived with colorectal cancer and what they themselves emphasize as important. The research questions analysed in this article are: What is important for persons diagnosed with colorectal cancer during their patient pathways? And, based on these findings, which significant features do the patient pathways share?" | Patients (adults between 18-70 with rectal cancer in treatment) | qualitative longitudinal research | Individual interviews and diaries | One year | 10 | 46 interviews |
| Hansen, L., Rosenkranz, S. J., Wherity, K., & Sasaki, A. (2017). Living with hepatocellular carcinoma near the end of life: Family caregivers' perspectives. Oncology Nursing Forum, 44(5), 562-570. | US | "To explore family caregivers perspectives about caring for patients with terminal HCC [Hepatocellular carcinoma] as the patients approached the end of life" | Family members (family caregivers of adult patients with a diagnosis of HCC) | Qualitative longitudinal research | Individual interviews | 6 months | 13 | 78 interviews |
| Hanson, C. L., Oliver, E. J., Dodd-Reynolds, C. J., & Allin, L. J. (2019). How do participant experiences and characteristics influence engagement in exercise referral? A qualitative longitudinal study of a scheme in Northumberland, UK. BMJ Open, 9(2), e024370-e024370. | UK | "This longitudinal qualitative study aimed to gain an insight into differential engagement through understanding participant experiences of an ERS [exercise referral scheme]" | People in the community (participants in ERS program) | qualitative longitudinal research | Individual interviews | 12 to 20 weeks | 15 | 26 interviews |
| Harris, M. (2017). Managing expense and expectation in a treatment revolution: Problematizing prioritisation through an exploration of hepatitis C treatment 'benefit'. The International Journal of Drug Policy, 47, 161-168. | UK | "To explore participants’ narratives of HCV [hepatitis C virus] treatment expectation and map their anticipatory accounts to those of post treatment ‘benefit." "This aim of this paper is to present analyses pertaining to participant treatment decision making, expectations and outcomes in a period of HCV biomedical transition and, through doing so, to explore the relevance and fit of contemporary public health discourses regarding ‘patient important benefits’ and HCV treatment prioritisation for this population." | Several: Patients and health-care providers (people living with hepatitis C and providers) | Qualitative longitudinal research | Individual interviews, and observations | 18 months | 28 | One hundred hours of clinic observations, number of interviews are not described |
| Harris, M., & Rhodes, T. (2018). Caring and curing: Considering the effects of hepatitis C pharmaceuticalisation in relation to non-clinical treatment outcomes. The International Journal On Drug Policy, 60, 24-32. | UK | "We consider the implications of simplified treatment provision in the era of direct acting antivirals (DAAs) for the realisation of non-clinical benefits., how engagement in HCV care is giving meaning for patient and provider" | Several: Patients and Health-care providers (patient with hepatitis C, and providers) | Ethnography & qualitative longitudinal research | Individual interviews, observations and field notes | Over 3 years | 38 | 84 interviews and over 100 h of observtions |
| Harvey, D., Foster, M., Quigley, R., & Strivens, E. (2018). Care transition types across acute, sub-acute and primary care. Journal of Integrated Care, 26(3), 189-198. | Australia | "The purpose of this analysis is to distil the types and commonalities of care transitions of older people by examining the individual experiences of older people, and their carers, who transitioned from community through acute and sub-acute care to home as a case study of transition." | Several: Patients and family members (older people and their carers) | Case study | Individual interviews, and service use data from medical records | about one month | 19 | 93 interviews |
| Harvey, D., Foster, M., Strivens, E., & Quigley, R. (2017). Improving care coordination for community-dwelling older Australians: A longitudinal qualitative study. Australian Health Review, 41(2), 144-150. | Australia | "To (1) describe care transition experiences from multiple perspectives and identify personal, systemic and local factors affecting these experiences; and (2) identify applied solutions that could be used to enhance implementation and capacity of the GEM [Geriatric Evaluation and Management] model." | Several: Patients, family members and health-care providers (patients, carers and service providers) | Case study | Individual interviews and focus groups interviews | about one month | 39 | 97 semistructured interviews |
| Harvey-Lloyd, J. M., Morris, J., & Stew, G. (2019). Being a newly qualified diagnostic radiographer: Learning to fly in the face of reality. Radiography, 25(3), e63-e67. | UK | "This study explored the experience of transition from student to practitioner in diagnostic radiography and in particular focused on being and becoming a radiographer." | Students (students in radiography) | Phenomenology | Individual interviews | 12 months | 9 | 27 interviews |
| Hatcher, A. M., Hofstedler, E. L., Doria, K., Dworkin, S. L., Weke, E., Conroy, A., Bukusi, E. A., Cohen, C. R., & Weiser, S. D. (first published 2019). Mechanisms and perceived mental health changes after a livelihood intervention for HIV-positive Kenyans: Longitudinal, qualitative findings. Transcultural Psychiatry, 2000, 57(1) 124–139. | Kenya | "We examined how a multi-sectoral livelihood intervention affected mental health for HIV positive farmers in Kenya. Using qualitative methodology, we explored participant definitions of mental health, perceived changes in mental health due to the intervention, and mechanisms through which the intervention may have influenced mental health outcomes." | Patients ( HIV-positive farmers in Kenya) | qualitative longitudinal research | Individual interviews | 12 months | 54 | 85 interviews |
| Henderson, G. E., Waltz, M., Meagher, K., Cadigan, R. J., Jupimai, T., Isaacson, S., Ormsby, N. Q., Colby, D. J., Kroon, E., Phanuphak, N., Ananworanich, J., Peay, H. L. (2019). Going off antiretroviral treatment in a closely monitored HIV "cure" trial: Longitudinal assessments of acutely diagnosed trial participants and decliners. Journal of the International AIDS Society, 22(3), e25260-e25260. | Thailand | "We aim to understand motivations to join or decline and whether, how, and why decision satisfaction may change over time" | Patients (people with HIV in ART trails) | not described | Individual interviews and questionnairs | 3 to 4 months | 14 | 36 interviews |
| Hill, C., Knafl, K. A., Docherty, S., & Santacroce, S. J. (2019). Parent perceptions of the impact of the Paediatric Intensive Care environment on delivery of family-centred care. Intensive & Critical Care Nursing, 50, 88-94. | US | "We conducted a secondary analysis of data from a longitudinal study of parent involvement in decision-making in an intensive care environment to further elaborate the role of the physical and cultural environment in parent perception of the delivery of FCC [family-centered care]." | Family members (parents to an infant in ICU) | Case study | Interviews (unclear if individual interviews or joint interviews) | about 12 months (varied between participants) | 6 | 61 interviews, approximately 1500 pages of data. |
| Hoag, J., Igler, E., Karst, J., Bingen, K., & Kupst, M. J. (2019). Decision-making, knowledge, and psychosocial outcomes in pediatric siblings identified to donate hematopoietic stem cells. Journal of Psychosocial Oncology, 37(3), 367-382. | US | "The current study was conducted to (1) describe the decision-making experience and psychosocial outcome of sibling donors utilizing a mixed-methods approach, and (2) determine the feasibility of completing a prospective and longitudinal assessment of sibling donors at a single institution." | Family members (sibling donors of Hematopoietic stem cell) | mixed methods | Interviews and questionnairs | Unclear, 6 months between time point 2 and 3 | 12 | 34 interviews |
| Hodson, T., Gustafsson, L., & Cornwell, P. (2019). "Just got to live life as it comes": A case study of the spousal-dyad longitudinal mild stroke transitional experience. Brain Injury, 33(9), 1200-1207. | Australia | "Consequently, the aim of this study was to provide an in-depth exploration of the mild stroke experience of one couple who received a novel health service within the Australian context. Specifically, this research examined the experience of transitioning from acute hospital care to the community following a mild stroke, for a spousal dyad in the first 9-months post-hospital discharge." | Several: Patients and family members (patient and spouse following a mild stroke) | Phenomenology & case study | Individual interviews | 9 months | 2 | 8 interviews |
| Hopia, H., Miettinen, S., Miettinen, M., & Heino-Tolonen, T. (2019). The voice of paediatric oncology nurses: A longitudinal diary study of professional development. European Journal of Oncology Nursing, 42, 28-35. | Finland | "The purpose of this paper is to describe how nurses perceive their professional development in paediatric oncology nursing by answering the question regarding the factors associated with professional development during the two-year training period. The rationale was that a deeper insight into the various aspects of professional development in paediatric oncology nursing from the nurses’ perspective would be gained in this. Furthermore, the objective was to conceptualise the phenomenon under the study." | Health care providers (nurses) | Qualitative longitudinal research | Diaries | 20 months | 17 | 304 pages of text |
| Horter, S., Bernays, S., Thabede, Z., Dlamini, V., Kerschberger, B., Pasipamire, M., Rusch, B., & Wringe, A. (2019). "I don't want them to know": How stigma creates dilemmas for engagement with Treat-all HIV care for people living with HIV in Eswatini. African Journal of AIDS Research, 18(1), 27-37. | Eswatini | "...we aim to examine how stigma shapes people living with HIV experiences with HIV, and engaging with HIV treatment and care services under Treat-all in Shiselweni, Eswatini (formerly Swaziland)." | Patients (people living with HIV) | Qualitative longitudinal research | Individual interviews | up to 12 months | 30 | 106 interviews |
| Horter, S., Wringe, A., Thabede, Z., Dlamini, V., Kerschberger, B., Pasipamire, M., Lukhele, N., Rusch, B., & Seeley, J. (2019). "Is it making any difference?" A qualitative study examining the treatment-taking experiences of asymptomatic people living with HIV in the context of Treat-all in Eswatini. Journal of The International AIDS Society, 22(1), e25220-e25220. | Eswatini | "We examine asymptomatic PLHIV’s [people living with HIV] experiences engaging with Treat-all care in the Kingdom of Eswatini (formerly named Swaziland), including how treatment-taking is navigated and motivated over the longer term (at least 12 months after initiation)." | Several: Patients and health-care providers (people with HIV and health care providers) | Qualitative longitudinal research | individual interviews and fieldnotes | about 8 months | 37 | 71 interviews |
| Horwood, C., Jama, N. A., Haskins, L., Coutsoudis, A., & Spies, L. (2019). A qualitative study exploring infant feeding decision-making between birth and 6 months among HIV-positive mothers. Maternal & Child Nutrition, 15(2), e12726-e12726. | South Africa | "This paper presents results from a study to explore infant feeding practices among HIV positive women and identify key role players influencing their infant feeding decisions. […] This study adopted a longitudinal qualitative design to prospectively capture critical moments and processes involved in infant feeding choices made by HIV‐infected women over the period from birth to 6 months. This methodology was chosen as the most appropriate method to explore the lived experience of change and capturing transitions" | Patients (HIV-positive mothers to infants) | Qualitative longitudinal research | Individual interviews | about 6 months | 11 | 61 interviews |
| Jama, N. A., Wilford, A., Haskins, L., Coutsoudis, A., Spies, L., & Horwood, C. (2018). Autonomy and infant feeding decision-making among teenage mothers in a rural and urban setting in KwaZulu-Natal, South Africa. BMC Pregnancy and Childbirth, 18(1), 52-52. | South Africa | "...explore teenage mothers' narratives about infant feeding choices and practices from birth to 6 months" | People in the community (women aged 15 years or older who were more than 36 weeks pregnant) | Qualitative longitudinal research | Individual interviews | 6 months | 10 | 60 interviews |
| Jama, N. A., Wilford, A., Masango, Z., Haskins, L., Coutsoudis, A., Spies, L., & Horwood, C. (2017). Enablers and barriers to success among mothers planning to exclusively breastfeed for six months: A qualitative prospective cohort study in KwaZulu-Natal, South Africa. International Breastfeeding Journal, 12, 1-13. | South Africa | "We report the findings of a longitudinal cohort study, conducted among mothers who planned to exclusively breastfeed their infants for six months, and describe the factors that facilitated or acted as barriers to achieving their goal." | People in the community (women aged 15 years or older who were more than 36 weeks pregnant) | Qualitative longitudinal research | Individual interviews | 6 months | 22 | 125 interviews |
| Janeiro, L. d. B., Ribeiro, E. M., & Lopez Miguel, M. J. (2018). What is inside the “black box”? Therapeutic community residents’ perspectives on each treatment phase. Addiction Research & Theory, 26(4), 294-305. | Portugal | "This study aims to describe residents’ perspectives on change through each of the three treatment phases The TC [therapeutic communication] residents’ perspectives on their change can contribute towards an understanding of this process of change. More specifically, we aimed to show how the residents perceived the contextual conditions of treatment during each phase, how they related to the perceived treatment and which outcomes they felt that they had attained." | People in the community (residents of therapeutic community) | Grounded theory | Individual interviews and questionnairs | 18 months | 25 | 427 documents |
| Jarvie, R. (2017). Lived experiences of women with co-existing BMI≥30 and Gestational Diabetes Mellitus. Midwifery, 49, 79-86. | UK | "The aim of the study was to explore the lived experiences of women with co-existing maternal obesity (BMI ≥ 30) and GDM [Gestational Diabetes Mellitus] during pregnancy and the post-birth period (< 3 months post-birth)." | Patients (pregnant women having co-existing maternal obesity and Gestational Diabetes Mellitus) | A qualitative, sociological design | Individual interviews and reflective field notes | 6 to 9 months | 27 | 63 interviews. |
| Jee, S. D., Schafheutle, E. I., & Noyce, P. R. (2017). Using longitudinal mixed methods to study the development of professional behaviours during pharmacy work-based training. Health & Social Care in the Community, 25(3), 975-986. | UK | "the aim of this study was to explore factors associated with changes in professional behaviours of trainees longitudinally during pre-registration training in pharmacy" | Several: Students and tutors (trainees and their main supervisors in pharmacies) | mixed methods | Individual interviews | 4 months | 41 | 120 interviews |
| Jensen, J. F., Overgaard, D., Bestle, M. H., Christensen, D. F., & Egerod, I. (2017). Towards a new orientation: A qualitative longitudinal study of an intensive care recovery programme. Journal of Clinical Nursing, 26(1), 77-90. | Denmark | "The aim of the study was to describe the patient experience of ICU [intensive care unit] recovery from a longitudinal perspective by analysing follow-up consultations at three time-points." | Patients (patient at ICU) | Qualitative longitudinal research | Audio-recordings of consultations, patient photographs, and reflection sheets | 9 to 11 months (depending upon case) | 12 | 36 audio-recorded consultations and 68 photographs |
| Jensen, A. M., Pedersen, B. D., Olsen, R. B., & Hounsgaard, L. (2019). Medication and care in Alzheimer's patients in the acute care setting: A qualitative analysis. Dementia, 18(6), 2173-2188. | Denmark | "The aim of the study was to investigate how oral medicine was administered to hip fracture patients with Alzheimer’s disease during acute hospital admission on an orthopaedic ward." | Patients (patients with an Alzheimer’s diagnosis) | Phenomenological and ethnogaphy | Observations and informal interviews | 4 days to 2 weeks (varied between cases) | 3 | 257 hours of observation |
| Jensen, A. M., Pedersen, B. D., Olsen, R. B., Wilson, R. L., & Hounsgaard, L. (first published 2018). "If only they could understand me!" Acute hospital care experiences of patients with Alzheimer's disease. Dementia, 2020, 19(7) 2332-2353. | Denmark | "The aim of this study was to investigate the observed experiences of patients with Alzheimer’s of receiving care in an acute hospital setting. This paper investigates the care of patients with Alzheimer’s disease in an acute setting, as co-morbidity to a hip fracture, and focuses on patients’ experiences of care during admission." | Patients (patients with dementia receiving acute care) | Ethnography | Observations and field notes | 4 to 14 days | 3 | Observations were conducted during 37 shifts (257 h) |
| Jindal-Snape, D., Johnston, B., Pringle, J., Kelly, T. B., Scott, R., Gold, L., & Dempsey, R. (2019). Multiple and multidimensional life transitions in the context of life-limiting health conditions: Longitudinal study focussing on perspectives of young adults, families and professionals. BMC Palliative Care, 18(1), 30-30. | UK | "(1) What multiple and multi-dimensional transitions are Young Adults [YA] experiencing due to their life-limiting health conditions and developmental stage? (2) What multiple and multi-dimensional transitions are significant others experiencing due to the life-limiting health conditions and developmental stage of the YAs?" | Several: Patients, family members and health-care providers (young adults with life limiting disease, their significant others, and health-care providers) | mixed methods & qualitative longitudinal research | Individual interviews | 6 months | 31 | 58 interviews |
| Johannessen, A., Engedal, K., Haugen, P. K., Dourado, M. C. N., & Thorsen, K. (2018). "To be, or not to be": Experiencing deterioration among people with young-onset dementia living alone. International Journal of Qualitative Studies on Health and Well-Being, 13(1), 1490620-1490620. | Norway | "aiming to explore the existential experiences and coping of people with YOD (age at onset of dementia before 65 years of age) , as they narrate the deterioration due to dementia that they go through over time." | Patients (people with young onset dementia living alone) | Grounded theory | Individual interviews | about 2 years | 10 | 42 interviews |
| Johannessen, A., Engedal, K., Haugen, P. K., Dourado, M. C. N., & Thorsen, K. (2019). Coping with transitions in life: A four-year longitudinal narrative study of single younger people with dementia. Journal of Multidisciplinary Healthcare, 12, 479-492. | Norway | "Therefore, we have performed a longitudinal study aiming to explore how people living alone with YOD [young people with dementia] experiences of coping with transitions during the progression of dementia." | Patients (people living alone with younger onset dementia) | Qualitative longitudinal research | Individual interviews | up to 48 months | 10 | 60 interviews |
| Johansen, F., Loorbach, D., & Stoopendaal, A. (2018). Exploring a transition in Dutch healthcare. Journal Of Health Organization And Management, 32(7), 875-890. | the Netherlands | "The purpose of this paper is to explore the contours of this transition in the Netherlands." | Policy-makers (health care organisations/project manager) | other | Individual interviews | 3 years | 22 | 33 interviews |
| Jones, K. F., Dorsett, P., Simpson, G., & Briggs, L. (2018). Moving forward on the journey: Spirituality and family resilience after spinal cord injury. Rehabilitation Psychology, 63(4), 521-531. | Australia | "The aim of this exploratory study was to consider how spirituality (encompassing meaning, hope and purpose), may facilitate family resilience after SCI [spinal cord injury] over time." | Several: Patients and family members (individuals with SCI, and family members) | Phenomenology | Joint interviews | 6 months | 20 | 19 interviews |
| Kerrissey, M., Satterstrom, P., Leydon, N., Schiff, G., & Singer, S. (2017). Integrating: A managerial practice that enables implementation in fragmented health care environments. Health Care Management Review, 42(3), 213-225. | US | "Our uniquely granular longitudinal data from 16 primary care clinics enable us to deeply explore the mechanisms that frontline workers use to overcome implementation barriers." | Health care providers (frontline workers at primary care clinics) | case study | Individual interviews, field notes from observations, and documents (e.g., meeting minutes) | 15 months | unclear, 16 clinics | 15 hours of observations, documents from 51 meetings, 18 interviews |
| Kirshbaum, M. N., Ennis, G., Waheed, N., & Carter, F. (2017). Art in cancer care: Exploring the role of visual art-making programs within an Energy Restoration Framework. European Journal Of Oncology Nursing, 29, 71-78. | Australia | "The aim of the study was to explore the experience of participation in a visual art-making program for people during or after cancer treatment in the Northern Territory of Australia, using a framework for energy restoration. The objectives of the study were: 1. To set up and facilitate an eight-week group experiential visual art-making program for people who have been diagnosed with cancer. 2. To document and analyse the participants' experiences of the arts program. 3. To interpret the findings using the Energy Restoration Framework." | Patients (people who have been diagnosed with cancer) | Qualitative longitudinal research | individual interviews and group discussions | 8 weeks | 8 | Unclear |
| Klinga, C., Hasson, H., Andreen Sachs, M., & Hansson, J. (2018). Understanding the dynamics of sustainable change: A 20-year case study of integrated health and social care. BMC Health Services Research, 18(1), 400-400. | Sweden | "The specific objective of this study is to gain insight into the dynamics of sustainable changes in integrated health and social care through an analysis of local actions that were trigged by a national policy." | Policy-makers (minutes of the model organization's steering-committee) | Case study | Documents ( meeting minutes and notes) | 20 years | Unclear | 98 documents |
| Kowalski, C. P., McQuillan, D. B., Chawla, N., Lyles, C., Altschuler, A., Uratsu, C. S., Bayliss, E. A., Heisler, M., & Grant, R. W. (2018). 'The hand on the doorknob': Visit agenda setting by complex patients and their primary care physicians. Journal of the American Board of Family Medicine, 31(1), 29-37. | US | "to investigate how patients and physicians prepare for visits, how visit agendas are determined, and how discussion priorities are established during time-limited visits. 1) examine how patients and physicians prepared for upcoming visits; 2) gain further insight into how agendas are set during visits; and 3) identify factors that facilitate or impede alignment of visit agendas." | Several: Patients and health-care providers (patients and their primary care physicians) | Qualitative longitudinal research | Individual interviews | Unclear, a few weeks | 57 | unclear, around 97 interviews |
| Križaj, T., Warren, A., & Slade, A. (2018). "Holding on to what I do": Experiences of older slovenians moving into a care home. Gerontologist, 58(3), 512-520. | Slovenia | "...to explore Slovenian older people’s experiences of transition into a care home and how it influenced their everyday engagement in meaningful occupations" | People in the community (older people) | Phenomenology | Individual interviews | 6 months | 6 | 18 interviews |
| Laerkner, E., Egerod, I., Olesen, F., & Hansen, H. P. (2017). A sense of agency: An ethnographic exploration of being awake during mechanical ventilation in the intensive care unit. International Journal of Nursing Studies, 75, 1-9. | Denmark | "The study aimed to explore patients’ experiences of being awake during critical illness and mechanical ventilation in the ICU {intensive care unit]." | Patients (patients’ being awake during critical illness and mechanical ventilation in the ICU) | other | Individual interviews and observations | 2 to 4 months (differ between participants) | 28 | 33 interviews, 102 days with observations between two and 12 hours a day |
| Lagsten, J., & Andersson, A. (2018). Use of information systems in social work - challenges and an agenda for future research. European Journal of Social Work, 21(6), 850-862. | Sweden | "The aim of this paper is therefore to contribute to the emerging social work information systems research agenda. Research questions: What are the challenges in the use of a social work information system in a Swedish social work agency according to the stakeholders? How do the empirically grounded challenges relate to the literature on the use of social work information systems? What are the important areas for further study in the evolving social work information systems research agenda?" | Health care providers (social workers) | Case study | Individual interviews, dialogue seminars, document, and observations | 10 years | 70 | Unclear |
| Lahav, O., Daniely, N., & Yalon-Chamovitz, S. (2018). Interpersonal social responsibility model of service learning: A longitudinal study. Scandinavian Journal of Occupational Therapy, 25(1), 61-69. | Israel | "The objective of this longitudinal study was to explore a structured model of Service-Learning (ISRSL), aimed towards the development of professional identity among OT [occupational therapy] students. In order to move beyond description a grounded theory approach was utilized to explore the perceptions and experiences of OT students who participated and experienced firsthand ISR-SL during their first academic year." | Students (occupational therapy students) | Grounded theory | Open-ended questions in questionnairs | 5 years | 105 | 183 questionnaires |
| Lang, H., France, E. F., Williams, B., Humphris, G., & Wells, M. (2018). The existence and importance of patients' mental images of their head and neck cancer: A qualitative study. Plos One, 13(12), e0209215-e0209215. | UK Scotland | "The aim of our study was to explore in detail the existence and importance of mental images of cancer among people with HNC in terms of the perceived origins and meaning of mental images, their development over time, and the relationship of their mental images to illness beliefs." | Patients (people with consecutive, newly-diagnosed head and neck cancer) | Phenomenology & qualitative longitudinal research | individual interviews, and images/drawings | 3 to 9.5 months (avarage 5 months) | 25 | 44 interviews |
| Larsen, S. M., Hounsgaard, L., Brandt, Å., & Kristensen, H. K. (2019). "Becoming acquainted": The process of incorporating assistive technology into occupations. Journal of Occupational Science, 26(1), 77-86. | Denmark | "The purpose of this study was, therefore, to investigate older adults’ experiences of the process of incorporating Ats [Assistive Technology] into occupations." | People in the community (older adults living in their usual home and who had applied to the municipality for an AT) | Qualitative longitudinal research and phenomenology | Individual interviews and observations | up to 10 months, most participants were followed 1,5 months | 8 | A total of 15 hours of individual interviews, and 16 hours of observation |
| Laur, C., Bell, J., Valaitis, R., Ray, S., & Keller, H. (2018). The Sustain and Spread Framework: Strategies for sustaining and spreading nutrition care improvements in acute care based on thematic analysis from the More-2-Eat study. BMC Health Services Research, 18(1), 930-930. | Canada | "The aim of this manuscript is to develop a potential framework of strategies to sustain and spread the successful implementation of INPAC [nutrition care improvements in acute care]." | Several: health-care providers and managers (Staff and management at five hospital sites) | not described | Individual interviews, Focus group interviews, and informal group discussions | 14 months | 138 | 57 individual interviews, 4 small group discussions, 11 focus group interviews |
| Lawton, J., Blackburn, M., Rankin, D., Allen, J. M., Campbell, F. M., Leelarathna, L., Tauschmann, M., Thabit, H., Wilinska, M.E., Elleri, D., & Hovorka, R. on behalf of the APCam11 Consortium. (2019). Participants' experiences of, and views about, daytime use of a day-and-night hybrid closed-loop system in real life settings: Longitudinal qualitative study. Diabetes Technology & Therapeutics, 21(3), 119-127. | UK | "To address these objectives, we report findings from interviews undertaken with individuals who used a hybrid day-and-night closed-loop system combined with pump suspend feature over 3 months. Given that studies have overwhelmingly focused upon nighttime use, we focus our reporting on people’s experiences of using the closed-loop system during the day." | Patients and health-care providers (individuals who used a hybrid day-and-night closed-loop system for diabetes treatment including the adolentce participants parents) | Qualitative longitudinal research | Individual interviews | 3 months | 24 | 48 interviews |
| Lawton, J., Blackburn, M., Breckenridge, J. P., Hallowell, N., Farrington, C., & Rankin, D. (2019). Ambassadors of hope, research pioneers and agents of change-individuals' expectations and experiences of taking part in a randomised trial of an innovative health technology: Longitudinal qualitative study. Trials, 20(1), 289-289. | UK | "As part of this investigation, we explored people’s reasons for taking part in the trial as well as their actual experiences of using the closed-loop system during the trial. As we describe in this paper, the findings from this aspect of our study not only prompted us to problematise use of dichotomous categories such as ‘self-interest’ and ‘altruism’, but also to contribute literature on clinical trials by considering how participants’ understandings of the trial and their complex and interweaving agendas for taking part could have profound implications for their conduct during the trial." | Several: Patients and family members (participants in a diabetes trial and parents to some participants that were 13-17 yrs old) | Qualitative longitudinal research | Individual interviews | 3 months | 24 | Unclear, drop outs not adressed |
| Lea, J., & Cruickshank, M. (2017). The role of rural nurse managers in supporting new graduate nurses in rural practice. Journal of Nursing Management, 25(3), 176-183. | Australia | "This paper reports on findings specifically related to the unique role rural nurse managers (NMs) and nurse unit managers (NUMs) play in supporting new graduate nurses transitioning to rural nursing practice." | Several: health-care providers and managers (nurses and nurse units managers) | case study | individual interviews | 9 months | 30 | 60 interviews |
| Lees, S., Marchant, M., & Desmond, N. (first published 2019). Addressing intimate partner violence using gender-transformative approaches at a community level in rural Tanzania: The UZIKWASA program. Journal of Interpersonal Violence, 2021, 36(13-14), NP7791-NP7812. | Tanzania | "Drawing on both innovative and traditional qualitative research methods, this article aims to explore the ways in which UZIKWASA’s [UZIKWASA is a civil society organization] interventions affect attitudes and norms surrounding violence against women and girls." | People in the community | Ethnography | Hearsay ethnographies (e.g., dairies), individual interviews, and focus group interviews | 5 years | 10 | More than a thousand diary entries |
| Leibring, I., & Anderzén-Carlsson, A. (2019). Fear and coping in children 5–9 years old treated for acute lymphoblastic leukemia - A longitudinal interview study. Journal of Pediatric Nursing, 46, e29-e36. | Sweden | "The aim of this study was to use a longitudinal perspective in a group of 5- to 9-year-old children with ALL [Acute lymphoblastic leukemia] to describe their ALL-related fears, the strategies they use to cope with those fears, and changes in their fears and strategies over time. 1. What fears do children with ALL experience at various time points during their treatment? 2. How do the children cope with their fears?" | Patients ( 5- to 9-year-old children with Acute lymphoblastic leukemia) | Qualitative longitudinal research | Individual interviews | about 2.5 years | 13 | 35 interviews |
| Lennon, M. R., Bouamrane, M.-M., Devlin, A. M., O'Connor, S., O'Donnell, C., Chetty, U., Agbakoba, R., Bikker, A., Grieve, E., Finch, T., Watson, N., Wyke, S., & Mair, F. S., (2017). Readiness for delivering digital health at scale: Lessons from a longitudinal qualitative evaluation of a national digital health innovation program in the United Kingdom. Journal Of Medical Internet Research, 19(2), e42-e42. | UK | "The aim of our study was to examine barriers and facilitators to implementation of digital health at scale through the evaluation of a £37m national digital health program: Delivering Assisted Living Lifestyles at Scale” (dallas) from 2012-2015. "The aim of this study was to capture barriers and facilitators to implementation of digital health across a wide range of stakeholders and across time, thus allowing us to answer the question of how ready” different people, processes, and systems are for mainstreaming digital health and to identify what measures might be taken to reduce some of the existing and persistent barriers in this area. Here we present our findings and conclude with a set of 10 recommendations to address some of the key readiness barriers identified." | Several: Health-care providers, patients and managers (key implementors, managers, patients, health-care providers of different professions) | Qualitative longitudinal research | Individual interviews, focus group interviews, questionnairs, documents, observations and field notes | 3 years | Unclear | 125 interviews, 7 focus group interviews, 12 project meetings, 16 observation sessions in the communities, 48 questionnairs, and 215 cross program documentary evidence on implementation |
| Lerret, S. M., Johnson, N. L., & Haglund, K. A. (2017). Parents' perspectives on caring for children after solid organ transplant. Journal for Specialists in Pediatric Nursing, 22(3), e12178. | US | "The purpose of this study was to explore parents’ perspectives on the discharge transition from acute hospitalization following SOT [solid organ transplant] to long-term management of a complex chronic condition. The time frame for transition to chronic condition care was defined as the first 6 months at home following SOT. The parents’ perspective may be used to inform discharge care and teaching in order to help providers anticipate issues that children and families may experience during the first 6 months after hospital discharge." | Family members (parents’ to children with solid organ transplant) | mixed methods | individual interviews | 6 months | 48 | 134 interviews |
| Lewis, M., Jones, A., & Hunter, B. (2017). Women's experience of trust within the midwife-Mother relationship. International Journal of Childbirth, 7(1), 40-52. | UK | "The broad aim of the research study was therefore to explore the concept of trust from the individual woman’s perspective with a view to developing a better understanding of trust within the midwife–mother relationship." | People in the community (women) | phenomenology | Individual interviews | 7 to 8 months | 9 | 27 interviews |
| Liang, P., Fleming, J., Gustafsson, L., & Liddle, J. (2017). Occupational experience of caregiving during driving disruption following an acquired brain injury. British Journal of Occupational Therapy, 80(1), 30-38. | Australia | "Therefore, the aim of this study is to explore, using a phenomenological approach, family members’ lived experiences of the occupations they take on during driving disruption following ABI [acute brain injury]." | Family members (family members to a person who have a brain injury) | phenomenology | individual interviews | 6 months | 15 | 42 interviews |
| Liang, P., Gustafsson, L., Liddle, J., & Fleming, J. (2017). Family members’ needs and experiences of driving disruption over time following an acquired brain injury: An evolving issue. Disability & Rehabilitation, 39(14), 1398-1407. | Australia | "Therefore, the aim of this study is to explore the family members’ needs and experiences of driving disruption of persons with ABI [acute brain injury] over time." | Family members (family members to a person who have a brain injury) | phenomenology & qualitative longitudinal research | individual interviews | 6 months | 14 | 41 interviews |
| Liddle, M. J., Baker, S. R., Smith, K. G., & Thompson, A. R. (2018). Young adults' experience of appearance-altering orthognathic surgery: A longitudinal interpretative phenomenologic analysis. The Cleft Palate-Craniofacial Journal, 55(2), 238-247. | UK | "The objective was to gain an experiential account of the processes of change associated specifically with orthognathic surgery in a way that might illuminate the psychological issues involved." | Patients (Patients aged 16-25 years who were scheduled to undergo orthognatic surgery) | Phenomenology | Individual interviews | 6 to 8 weeks | 7 | 14 interviews |
| Lindberg, K., Mørk, B. E., & Walter, L. (2019). Emergent coordination and situated learning in a Hybrid OR: The mixed blessing of using radiation. Social Science & Medicine, 228, 232-239. | Sweden | "Drawing upon a longitudinal, qualitative study of a Hybrid Operating Room in Sweden, we illustrate how the staff from a variety of medical specialties need to coordinate their tasks and competencies, and learn how to use the technology in a safe way." (from abstract) | Several: Health-care providers and managers (nurses and doctors working in the Hybrid OR, but also with hospital technicians, physicists and representatives of the suppliers of the technology) | Ethnography & qualitative longitudinal research | Observations and shadowing, individual interviews, and documents | 4 years | unclear | 60 interviews (80 h), observation and shadowing for a total of 162 hours |
| Lindberg, K., Walter, L., & Raviola, E. (2017). Performing boundary work: The emergence of a new practice in a hybrid operating room. Social Science & Medicine (1982), 182, 81-88. | Sweden | "In this paper, we investigate the boundary work performed in relation to the boundaries in a setting where a new practice is emerging in the midst of other established practices. Rather than analyzing such boundary work as a rhetorical style, like Gieryn, we use Akrich and Latour, (1992) scripting processes as our analytical lens for exploring how boundary work is performed in practice." | Health care providers (surgical nurses, anastesia nurses, surgigal healthcare assistents, radiology nurses) | Qualitative longitudinal research | Observations, individual interviews and documents | about 2.5 years | unclear | 42 interviews, 134 hours of observations |
| Lut, I., Evangeli, M., & Ely, A. (2017). “When I went to camp, it made me free”: A longitudinal qualitative study of a residential intervention for adolescents living with HIV in the UK. Children & Youth Services Review, 79, 426-431. | UK | "This UK study presents a qualitative investigation of young peoples' experience of a residential support camp conducted both at the end of the intervention and six months after, using representative sampling methods. It aimed to answer what the experiences and perceived consequences of attending camp were over time." | Patients ( adolescents living with HIV) | Qualitative longitudinal research | individual interviews | 6 months | 11 | 19 interviews |
| Malin, H., Liauw, I., & Damon, W. (2017). Purpose and character development in early adolescence. Journal of Youth & Adolescence, 46(6), 1200-1215. | US | "The goal of this study was to qualitatively describe how early adolescents show purpose—in what aspects of life and through what types of actions do they pursue purpose? We further sought to describe the relationship that purpose has to other character strengths (gratitude, compassion, and grit) at this early stage of development, to better understand whether they share a developmental trajectory and how they might differ early in development. Moreover, we wanted to explore the possible developmental interactions among the character strengths. For example, does compassion promote purpose development? Does purpose support grit? Our analysis cannot fully answer these developmental questions, but sets the course for further research by qualitatively exploring these questions: What does purpose look like in early adolescence? How is purpose similar to, and different from, other related character strengths? And, how does purpose interact with other character strengths in early adolescence?" | People in the community (early adolescents) | not described | Individual interviews and questionnairs | about a year | 98 participants interviewed, and 1.366 students completed the questionnairs | 2,371 questionnairs, and 182 interviews |
| Marshall, S., Reidlinger, D. P., Young, A., & Isenring, E. (2017). The nutrition and food-related roles, experiences and support needs of female family carers of malnourished older rehabilitation patients. Journal of Human Nutrition and Dietetics, 30(1), 16-26. | Australia | "What are the nutrition and food-related roles, experiences and support needs of female family carers of community dwelling malnourished older adults admitted to rehabilitation units in rural New South Wales (NSW), Australia, both during admission and following discharge?" | Family members (female family carers of community dwelling malnourished older adults) | phenomenology & qualitative longitudinal research | individual interviews | about 2 weeks | 4 | 8 interviews |
| McGeechan, G. J., McPherson, K. E., & Roberts, K. (2018). An interpretative phenomenological analysis of the experience of living with colorectal cancer as a chronic illness. Journal of Clinical Nursing, 27(15), 3148-3156. | UK | "The aim of this study was to qualitatively explore the psychosocial and physical consequences of living with colorectal cancer as a chronic illness and how this changes survivor’s views and plans for their future, over time." | Patients (patients with colorectal cancer who attending oncology follow-up clinics) | Phenomenology & qualitative longitudinal research | Individual interviews | 6 months | 6 | 11 interviews |
| McKay, V. R., Dolcini, M. M., & Catania, J. A. (2017). Impact of human resources on implementing an evidence-based HIV prevention intervention. AIDS and Behavior, 21(5), 1394-1406. | US | "Using the Interactive Systems Framework, we explored staff fluctuation and the subsequent influence on RESPECT, an HIV prevention EBI [evidence based interventions]." (from abstract) | Health care providers (staff responsible for providing RESPECT directly to clients) | not described | Individual interviews and questionnars | 12 months | 53 | 90 interviews |
| McKeganey, N., & Barnard, M. (2018). Change and Continuity in Vaping and Smoking by Young People: A Qualitative Case Study of A Friendship Group. International Journal of Environmental Research and Public Health, 15(5), 1008. | UK Scotland | "In this paper, we report data from a research study that was designed to explore the possible fluidity in young peoples’ perceptions and engagement with e-cigarettes by re-interviewing the members of a small teenage friendship group in Glasgow, Scotland, focusing on how their relationship and perception of e-cigarettes changed over a six-month period." | People in the community (young people) | Case study | Individual interviews | 6 months | 8 | 15 interviews |
| McKenzie, S. A., Rasmussen, K. M., & Garner, C. D. (2018). Experiences and perspectives about breastfeeding in “public”: A qualitative exploration among normal-weight and obese mothers. Journal of Human Lactation, 34(4), 760-767. | US | "The aims of this study were to (a) describe U.S. women’s experiences with breastfeeding in public and (b) describe how obese women’s experiences compared with normal-weight women’s experiences." | People in the community (pregnant women in central New York who intended to breastfeed) | Qualitative longitudinal research | Individual interviews and fieldnotes | about 9 months | 26 | 103 interviews |
| Meijer, E., Vangeli, E., Gebhardt, W. A., & van Laar, C. (first published 2018). Identity processes in smokers who want to quit smoking: A longitudinal interpretative phenomenological analysis. Health, 2020, 24(5), 493-517. | The Netherlands | "We investigated in-depth how smokers’ sense of identity may change during the process of quitting, and what happens to their sense of identity if they cannot quit successfully." | People in the community (daily smokers who intended to quit smoking within 2 months) | Phenomenology & qualitative longitudinal research | Individual interviews and questionnairs | 22 months | 10 | 30 interviews and 9 follow up surveys |
| Milbourn, B., McNamara, B., & Buchanan, A. (2017). A qualitative study of occupational well-being for people with severe mental illness. Scandinavian Journal Of Occupational Therapy, 24(4), 269-280. | Australia | "The purpose of this paper is to investigate the occupational well-being of Western Australian people diagnosed with an SMI [severe mental illness], who are under ACT [Assertive Community Treatment] care and living in the community, by use of the Occupational Well-being framework. The study seeks to determine if their occupational needs, as presented in the framework, are being met." | Patients (people diagnosed with an severe mental illness) | not described | individual interviews | 12 months | 11 | 80 interviews |
| Minton, C., Batten, L., & Huntington, A. (2019). A multicase study of prolonged critical illness in the intensive care unit: Families' experiences. Intensive & Critical Care Nursing, 50, 21-27. | New Zealand | "The purpose of this study, which forms one part of a larger study, is to explore the experiences of family of ICU [intensive care unit] patients with a prolonged critical illness." | Family members (family of ICU patients) | Case study | Observation described in field notes, in-depth interviews and informal conversations | 17 to 66 weeks | 16 | 160h of observations |
| Minton, C., Batten, L., & Huntington, A. (2018). The impact of a prolonged stay in the ICU on patients’ fundamental care needs. Journal of Clinical Nursing, 27(11), 2300-2310. | New Zealand | "To explore patients’, families’ and health professionals’ experiences of a long-stay patient in an intensive care unit." (from abstract) | Several: Patients, family members and health-care providers (patients’, families’ and health professionals’ in intensive care unit) | case study | observations, interviews, informal conversations, field notes and documents. | 17 to 66 days | 47 | Unclear |
| Mitterlechner, M. (2018). Governing integrated care networks through collaborative inquiry. Journal of Health Organization and Management, 32(7), 860-874. | Switzerland | "The purpose of this paper is to develop a theory of governing in integrated care networks. Asking how and why the governance of these networks emerges and evolves over time, it responds to calls for more innovative thinking in this field." | Policy-makers (project meetings at healthcare Centres) | case study | semi-structured interviews, participant observations and archival data. | Data was collected prospectively over 4 years, documents were collected another 5 years back | Unclear | 35 individual interviews. Observations at 96 meetings (234 pages of observational notes and 92 pages of field notes), documents (e.g., e-mails, meeting documents and minutes) encompassed 2,500 pages. |
| Monaro, S., West, S., Pinkova, J., & Gullick, J. & Pinkova, J. (2018). The chaos of hospitalisation for patients with critical limb ischaemia approaching major amputation. Journal of Clinical Nursing, 27(19), 3530-3543. | Australia | "To illuminate the hospital experience for patients and families when major amputation has been advised for critical limb ischaemia (CLI)." ( from abstract) "This study, from the same sample, reports only the experiences of hospitalisation and therefore aims to illuminate the hospital lifeworld of patients with CLI who have been advised to have an amputation, in order to acquire a better understanding of the experience and to inform improvements in person-centred, interdisciplinary hospital care." (from article) | Several: Patients and family members (patients and families when major amputation has been advised for critical limb ischaemia) | phenomenology | Individual interviews | 6 months | 27 | 42 interviews |
| Monrouxe, L. V., Bullock, A., Gormley, G., Kaufhold, K., Kelly, N., Roberts, C. E., Mattick, K., & Rees, C. (2018). New graduate doctors' preparedness for practice: a multistakeholder, multicentre narrative study. BMJ Open, 8(8), e023146-e023146. | UK (England, Northern Ireland, Scotland and Wales) | "We aim to explore issues around preparedness for practice in terms of how the concept is understood across a range of stakeholder groups and to understand aspects in which new medical graduates are deemed prepared (or unprepared) for clinical practice with the following two broad research questions (RQ): ► RQ1: How do stakeholders conceptualise ‘preparedness for practice’? ► RQ2: To what extent do various stakeholders perceive recent medical graduates to be prepared for practice, and what factors do they attribute to this?" | Several: Health-care providers, students, teachers, managers, policy-makers (newly graduated doctors, clinical educators, training programme leads, nurses, pharmacists, managers, policy and government officials) | not described | individual interviews, focus group interviews, and audio diaries | 3 months | 185 | 27 focus group interviews, 84 individual interviews, 254 audio diary entries |
| Moore, A. M., Dennis, M., Anderson, R., Bankole, A., Abelson, A., Greco, G., & Vwalika, B. (2018). Comparing women's financial costs of induced abortion at a facility vs. seeking treatment for complications from unsafe abortion in Zambia. Reproductive Health Matters, 26(52) 1522195. | Zambia | "This longitudinal study assesses the costs of abortion for the woman and her family, comparing women who obtained an abortion at a facility with those who arrived at a health facility experiencing abortion complications." | Several: People in the community, family members (women and their family) | not described | Interviews and questionnairs | 3 to 4 months | 38 | 76 interviews |
| Morris, R. L., & Sanders, C. (2018). Critical moments in long-term condition management: A longitudinal qualitative social network study. Chronic Illness, 14(2), 119-134. | UK | "The aim of this paper is to explore how long-term condition management changes over time and the influence of social network members on where and how people seek support." | Patients (Individuals who had either diabetes, irritable bowel syndrome, or chronic obstructive pulmonary disease) | not described | Individual interviews and drawings/maps | 12 months | 30 | 85 interviews, 26 drawings |
| Morrow, V., Tafere, Y., Chuta, N., & Zharkevich, I. (2017). "I started working because I was hungry": The consequences of food insecurity for children's well-being in rural Ethiopia. Social Science & Medicine 182, 1-9. | Ethiopia | "By exploring children's accounts of their experiences, we highlight the effects of food insecurity on children's well-being in Ethiopia, how food insecurity affects crucial decisions over the life course and how these differ by gender; and the importance of sources of support over time." | People in the community (rural children with experience of food insecurity) | Case study & qualitative longitudinal research | Individual interviews, group discussions, and creative methods | 7 years | 8 | unclear |
| Mozaffar, H., Cresswell, K. M., Williams, R., Bates, D. W., & Sheikh, A. (2017). Exploring the roots of unintended safety threats associated with the introduction of hospital ePrescribing systems and candidate avoidance and/or mitigation strategies: A qualitative study. BMJ Quality & Safety, 26(9), 722-733. | UK | "We therefore revisited the large body of ethnographic evidence generated from the cases to review the evidence for roots of reported unintended safety threats associated with the introduction of ePrescribing in design, implementation and use, in order to develop a taxonomy of these factors, and use these insights to shed light on possible risk mitigation strategies." | Several: Health-care providers, managers, policy makers | Ethnography, case study & qualitative longitudinal research | Individual interviews, observation, and documents (e.g., project plans, risk logs and business cases) | 2 years | unclear | 242 interviews, 32.5 h of observations, 55 documents |
| Mueller, A. S., Jenkins, T. M., Osborne, M., Dayal, A., O'Connor, D. M., & Arora, V. M. (2017). Gender differences in attending physicians' feedback to residents: A qualitative analysis. Journal of Graduate Medical Education, 9(5), 577-585. | US | "...in this study, our aim was to use qualitative data to better understand the lagging performance evaluations of female EM [emergency medicine] residents in PGY-3 [postgraduate year 3]." | Teachers | not described | text comments from student evaluations | 2 years | 67 | 47 evaluations |
| Munford, R., & Sanders, J. (2019). Harm, opportunity, optimism: Young people's negotiation of precarious circumstances. International Social Work, 62(1), 185-197. | New Zealand | "The study aimed to develop an understanding of the lived experiences, contexts and transitions of young people who faced high levels of adversity throughout their childhood. Of particular interest was investigating identity development and experiences of education and employment. The first-person accounts and those of their trusted others enabled an exploration of how these young people mediated challenging circumstances and negotiated for resources and support from both informal networks and formal service systems." | Several: People in the community, family members (young people who faced high levels of adversity, and their trusted others) | not described | Individual interviews | about 3 years | 107 | 507 interviews |
| Musesengwa, R., Chimbari, M. J., & Mukaratirwa, S. (2017). Initiating community engagement in an ecohealth research project in Southern Africa. Infectious Diseases of Poverty, 6(1), 22-22. | Botswana, South Africa and Zimbabwe | "This paper aims to outline the process of initiating community engagement in an ecohealth study and to describe the issues emerging from its development and implementation." | Several: People in the community, policy-makers, health-care providers (headmen, community liaison officers, principal investigators, country coordinators, project team members, CAB members, community researcher assistants, nurses) | Case study | Individual interviews, participatory workshops, unstructured interviews and direct observations | 18 months | 17 | unclear |
| Musto, M. (2019). Brilliant or bad: The gendered social construction of exceptionalism in early adolescence. American Sociological Review, 84(3), 369-393. | US | "...to identify the processes by which educators’ differential responses to boys’ rule-breaking by course level produced gender differences in students’ perceptions of intelligence. AND to illustrate how race intersected with gender when shaping students’ perceptions of intelligence. AND Do students’ gender beliefs about intelligence and exceptionalism vary by course level? If so, what are the processes encouraging students to perceive girls and boys as having different dispositions toward school, and how do their beliefs differ by course level? Does race intersect with gender when shaping higher- and lower-level students’ gender beliefs about intelligence and exceptionalism? If so, how?" | Several: Students and teachers | Ethnography | Individual interviews, focus group interviews, and observations | about 2.5 years | 196 | 196 interviews. Number/hours of observations, not described. |
| Myrin Westesson, L., Wallengren, C., Baghaei, F., & Sparud-Lundin, C. (2018). Reaching independence through forced learning: Learning processes and illness management in parents of children affected by hemophilia. Qualitative Health Research, 28(14), 2142-2154. | Sweden | "The aim of this study therefore is to explore parents’ learning processes and illness management in daily life during the first year after the start of their child’s treatment." | Family members (parents of children with hemophilia) | grounded theory & qualitative longitudinal research | individual interviews and joint interviews | 12 to 14 months | 8 | 30 interviews |
| Namukwaya, E., Murray, S. A., Downing, J., Leng, M., & Grant, L. (2017). 'I think my body has become addicted to those tablets'. Chronic heart failure patients' understanding of and beliefs about their illness and its treatment: A qualitative longitudinal study from Uganda. Plos One, 12(9), e0182876-e0182876. | Uganda | "This study addresses a gap identified by Selman et al in a review of literature on HF [heart failure] in Africa in 2015 which highlighted the need for culturally sensitive research on patients’ experiences to explore if issues such as communication difficulties observed in high-income countries also exist for HF patients in Uganda." | Patients (patients with heart failure) | grounded theory & qualitative longitudinal research | individual inteviews and joint interviews | 6 months | 21 | 40 individual interviews, 4 joint interviews |
| Naraine, M. D., Fels, D. I., & Whitfield, M. (2018). Impacts on quality: Enjoyment factors in blind and low vision audience entertainment ratings: A qualitative study. Plos One, 13(12), e0208165-e0208165. | Canada | "The research questions are: 1) what is the longitudinal impact on B/LV [blind and low vision] audiences of the Canadian integrated model of description for an eight-part television comedy; and 2) what are the positive and negative factors identified by users for the AD [audion description] and the show? We hypothesize that the longitudinal enjoyment of the show’s and the quality of the AD (as assessed by viewers) will be positive and that there will be a positive response to the integrated AD related to fit with the show style and describer’s voice characteristics such as pace, language and emotional match. We examine and report on the impact this Canadian-originated approach [2] has on Canadian B/LV [Blind and low vision] viewers over time, considering the emotional impact on B/LV viewers and their responses to this AD [audio description] approach." | People in the community (people being blind or low vision) | not described | open ended questions in questionnaires | 2 months | 24 | Unclear |
| Nash, B. H., & Mitchell, A. W. (2017). Longitudinal study of changes in occupational therapy students' perspectives on frames of reference. The American Journal Of Occupational Therapy, 71(5), 7105230010p7105230011-7105230010p7105230017. | US | "The purpose of this longitudinal study was to explore students’ views of FoR [frames of reference] as they progressed through the didactic portion of an occupational therapy program and participated in Level I fieldwork. The existing research has tended to focus on how students learn and apply theory, MoP, [models of practice] and FoR throughout coursework and Level II fieldwork. This study was designed to address the following question: How do occupational therapy students’ perspectives of the value of FoR change over the course of the didactic portion of an occupational therapy program?" | Students (occupational therapy students) | phenomenology | Individual interviews | 15 months | 34 | unclear |
| Nešporová, O. (2019). Hazy transition to fatherhood: The experiences of Czech fathers. Journal of Family Issues, 40(2), 143-166. | Czech Republic | "The study focuses on the impacts of fatherhood on the everyday lives of new fathers, while taking into account issues surrounding the involvement of fathers and the various cultural constructs of fatherhood." | Family members (fathers, but also a few mothers) | Qualitative longitudinal research | Individual interviews | up to 21 months | 32 | 64 interviews |
| Nicholas, D. B., Barrera, M., Granek, L., D'Agostino, N. M., Shaheed, J., Beaune, L., Bouffet, E., & Antle, B. (2017). Parental spirituality in life-threatening pediatric cancer. Journal of Psychosocial Oncology, 35(3), 323-334. | Canada | "...this paper specifically focuses on how parents experienced and navigated spirituality and faith during the illness trajectory. The following questions are addressed: (1) What is the role of spirituality? (2) What is the relationship between spirituality and hope? and (3) How may spirituality change as a result of having a child with a poor prognosis?" | Family members (parents having a child with a poor prognosis) | grounded theory | Individual interviews | 9 months | 35 | 92 interviews |
| Nichols, V. P., Williamson, E., Toye, F., & Lamb, S. E. (2017). A longitudinal, qualitative study exploring sustained adherence to a hand exercise programme for rheumatoid arthritis evaluated in the SARAH trial. Disability & Rehabilitation, 39(18), 1856-1863. | UK | "The aim of this parallel interview study was to explore the trial participants’ experiences of the exercise programme and, in particular, how successfully they adhered to the programme over time. We chose a longitudinal study design to investigate the transition from supervised exercise to independent exercise, seeking insight into facilitators and barriers to exercise and changes in symptoms/experience over time. Understanding this process is a crucial part of developing an effective implementation strategy to facilitate the uptake of the SARAH exercise programme into clinical practice." | Patients ( adults diagnosed with RA reporting pain and dysfunction of hands and who were either not on medication or on a stable drug regime for three months or more) | phenomenology & qualitative longitudinal research | individual interviews | 8 months | 14 | 27 interviews |
| Nightingale, J., Hardy, M., & Snaith, B. (2018). Embedding consultant radiographer roles within radiology departments: A framework for success. Radiography, 24(4), 289-297. | UK | "This article discusses the design, implementation and validation of an outcomes framework for benchmarking competencies for trainee or new-in-post consultant radiographers." | Health care providers (radiographers) | Phenomenology & qualitative longitudinal research | Individual interviews | 5 years | 5 | Unclear |
| Nilsson, K., Bååthe, F., Andersson, A. E., Wikström, E., & Sandoff, M. (2017). Experiences from implementing value-based healthcare at a Swedish university hospital - An longitudinal interview study. BMC Health Services Research, 17(1), 169-169. | Sweden | "This study explores how the representatives of four pilot project teams experienced implementing VBHC [value based healthcare] over a period of 2 years in four different groups of patients at a large Swedish University Hospital." | Several: Policy-maker, managers, health care providers (healthcare developer, physicians and heads of department) | not described | individual interviews | about 20 months | 20 | 59 interviews |
| Nixon, S. A., Bond, V., Solomon, P., Cameron, C., Mwamba, C., Hanass-Hancock, J., Margaret C. Maimbolwa, J. Menon, A., Simwaba, P., Sinyinza, R., Siwale, M., Tattle, S., & Yates, T. (2018). Optimism alongside new challenges: Using a rehabilitation framework to explore experiences of a qualitative longitudinal cohort of people living with HIV on antiretroviral treatment in Lusaka, Zambia. AIDS Care, 30(3), 312-317. | Zambia | "This longitudinal qualitative study used a rehabilitation science approach to explore the experiences over time of women and men living with HIV and on antiretroviral therapy (ART) in the high HIV-prevalence setting of Lusaka, Zambia." | Patients (women and men living with HIV and on ART) | Qualitative longitudinal research | Individual interviews | about 12 months | 35 | 99 interviews |
| Nizza, I. E., Smith, J. A., & Kirkham, J. A. (2018). 'Put the illness in a box': A longitudinal interpretative phenomenological analysis of changes in a sufferer's pictorial representations of pain following participation in a pain management programme. British Journal of Pain, 12(3), 163-170. | UK | "This article presents a single case from a wider study where IPA [interpretive phenomenological analysis] interviews with drawings were used longitudinally, to understand how pain and the sense of identity of sufferers changed following participation in a pain management programme (PMP). " | Patients (woman with fibromyalgia, degenerated discs and depression) | Case study and phenomenology | Individual interviews, and drawings | 9 months | 1 | 6 drawings, 3 interviews |
| Nordin, A., Andersson Gäre, B., & Andersson, A.-C. (2017). Emergent programme theories of a national quality register - A longitudinal study in Swedish elderly care. Journal of Evaluation in Clinical Practice, 23(6), 1329-1335. | Sweden | "The purpose of this study is to examine and establish the PTs [programme theories] of SA [Senior alert] in CMSs [clinical microsystems] at work units in elderly care. By comparing their PTs with that of the initiator, the paper reports on how PTs in CMSs emerge in relation to the established PT." | Policy-makers (senior alert experts) | case study & qualitative longitudinal research | Individual interviews | about 2 years | 15 | 22 interviews |
| Ober, J. L., & Lape, J. E. (2019). Cultivating acute care rehabilitation team collaboration using the kawa model. Internet Journal of Allied Health Sciences & Practice, 17(3), 1-8. | US | "...to investigate the impact of a teambuilding intervention with use of the Kawa model on acute care rehabilitation team collaboration" | Health care providers (rehabilitation staff members) | not described | Questionnairs with open-ended questions | 5 weeks | 8 | 16 questionnairs (3 open-ended questions per questionnair) |
| Pappne Demecs, I., & Miller, E. (2019). Participatory art in residential aged care: A visual and interpretative phenomenological analysis of older residents' engagement with tapestry weaving. Journal of Occupational Science, 26(1), 99-114. | Australia | "In the ‘Tapestry of Home’ project described here, a professional tapestry artist moved her practice and a 1.8 by 2 meter loom into a residential aged care for 6 months to creatively engage residents. This paper explores if and how creative occupation, a participatory art project, might benefit older people living in residential aged care." | Patients (older people living in residential aged care) | phenomenology, case study & participatory research | Observations, field notes, researcher produced photographs and videos, informal conversations, and individual interviews. | 6 months | 3 | Unclear |
| Parappilly, B. P., Mortenson, W. B., Field, T. S., & Eng, J. J. (first published 2019). Exploring perceptions of stroke survivors and caregivers about secondary prevention: A longitudinal qualitative study. Disability and Rehabilitation, 2020, 42(14), 2020-2026. | Canada | "...to explore how the perceived barriers and facilitators associated with participation in secondary prevention activities change over the early stroke recovery period among stroke survivors and their family members." | Several: Patients and family members (stroke survivors and their familiy member) | Qualitative longitudinal research | Individual interviews | 6 months | 28 | 52 interviews |
| Parker, S., & Mayock, P. (2019). "They're always complicated but that's the meaning of family in my eyes": Homeless youth making sense of "family" and family relationships. Journal of Family Issues, 40(4), 540-570. | Ireland | "(a) What is the nature and shape of homeless youths’ relationships with their families? (b) In what way, if at all, do these relationships change over time? and (c) How do homeless young people construct and make sense of “family” in the context of their own family experiences?" | Several: People in the community and family members (homeless youth age 16-24 and their family member) | Qualitative longitudinal research | Individual interviews | about 2 years | 50 | 87 interviews |
| Parkinson, P., & Cashmore, J. (2018). Relocation and the indissolubility of parenthood. Journal of Child Custody, 15(1), 76-92. | Australia | "This article is based upon the findings of a five-year prospective longitudinal study of relocation disputes in Australia, involving interviews with 80 parents and 33 children in 70 families." | Several: People in the community and family members (parents and children i families) | Qualitative longitudinal research | Individual interviews | 18 months to 2 years | 113 | Unclear |
| Payne, S., Eastham, R., Hughes, S., Varey, S., Hasselaar, J., & Preston, N. (2017). Enhancing integrated palliative care: what models are appropriate? A cross-case analysis. BMC Palliative Care, 16(1), 64-64. | UK | "We aimed to investigate accounts of hospice integration with local health care providers, using the framework provided by the model in Fig. 1, to determine how service users and healthcare professionals perceived palliative care services and the extent of integration experienced. In addition, we seek to investigate practices associated with care as experienced by patients, family carers and health professionals which promote or limit integration." | Several: Patients, family members and health-care providers | case study | individual interviews, joint interviews, and focus group interviews | 3 months | 70 | Unclear, around 84 individual/joint interviews and 4 focus group interviews |
| Peek, S. T. M., Luijkx, K. G., Vrijhoef, H. J. M., Nieboer, M. E., Aarts, S., van der Voort, C. S., Rijnaard, M. D., & Wouters, E. J. M. (2019). Understanding changes and stability in the long-term use of technologies by seniors who are aging in place: A dynamical framework. BMC Geriatrics, 19(1), 236-236. | The Netherlands | "In the current qualitative study, DST [Dynamical Systems Theory] is used as a theoretical lens while addressing the following research questions: (1) When and why does the frequency of use of technology by independentliving older adults remain stable over time; and (2) What drives changes in the frequency of use of technology by independent-living older adults." | People in the community (independent living older adults) | not described | Individual interviews, questionnairs, and observations of technologies in the participants homes | About 19 months | 33 | 99 interviews |
| Peek, S. T. M., Luijkx, K. G., Vrijhoef, H. J. M., Nieboer, M. E., Aarts, S., van der Voort, C. S., Rijnaard, M.D., & Wouters, E. J. M. (2017). Origins and consequences of technology acquirement by independent-living seniors: Towards an integrative model. BMC Geriatrics, 17(1), 189-189. | the Netherlands | "The current study aimed to understand the origins and consequences of technology acquirement by independent living older adults. We did this by exploring: (1) how and why technologies are acquired by independent-living older adults; and (2) the implications of the ways in which independent-living older adults acquire technologies." | People in the community (independent-living older adults) | Qualitative longitudinal research | Individual interviews, observations | 18 months | 33 | 99 interviews |
| Pemo, K., Phillips, D., & Hutchinson, A. M. (2019). An exploration of breastfeeding practices by Bhutanese women. Journal of Human Lactation, 35(1), 181-191. | Bhutan | "...to explore Bhutanese women’s views, intentions, and experiences related to exclusive breastfeeding" | People in the community (women pregnant for the first time) | Qualitative longitudinal research | Individual interviews | about 6 weeks | 24 | 46 interviews |
| Perry, J., Wöhlke, S., Heßling, A. C., & Schicktanz, S. (2017). Why take part in personalised cancer research? Patients' genetic misconception, genetic responsibility and incomprehension of stratification-an empirical-ethical examination. European Journal of Cancer Care, 26(5), e12563. | Germany | "We examined the motivation for participation in the clinical trial from the patients' perspective" | Patients (colorectal cancer patients) | longitudinal empirical-ethical study | observations, and individual interviews | 60 weeks | 40 | 36 physician-patient consultations, and 75 Intervews |
| Peruzzolo, D. L., Barbosa, D. M., & Ramos de Souza, A. P. (2018). Occupational therapy and babies treatment in premature intervention from a hypothesis of psychomotor functioning: Single case study. Brazilian Journal of Occupational Therapy, 26(2), 409-421. | Brazil | "To analyze the effectiveness of a Hypothesis of Psychomotor Functioning (HPF) for treatment of the premature babies" | Several: Patients, health-care providers (premature babies and occupational therapists) | Case study | Individual interview, video recorded observations, and diaries. | 3 months | 1 | Unclear |
| Phipps, D. L., Jones, C. E. L., Parker, D., & Ashcroft, D. M. (2018). Organizational conditions for engagement in quality and safety improvement: A longitudinal qualitative study of community pharmacies. BMC Health Services Research, 18(1), 783-783. | UK | "...to understand what is needed for successful improvement efforts in community pharmacies, both to ensure that they make an effective contribution to primary care quality and safety in their own right, and to identify general insights about improvement that might be applicable to other areas." | Health care providers (community pharmacies) | Qualitative longitudinal research | Observations, field notes, and focus group interviews | 12 months | 10 | Unclear |
| Poland, F., Spalding, N., Gregory, S., McCulloch, J., Sargen, K., & Vicary, P. (2017). Developing patient education to enhance recovery after colorectal surgery through action research: A qualitative study. BMJ Open, 7(6), e013498-e013498. | UK | "This study aimed to examine the perceived value of patient education for patients undergoing colorectal surgery for cancer as one component of an ERAS multimodal approach, and how changing the education might better support enhanced recovery by supporting patient self-management." "The study objectives were to understand the role of preoperative education for patients undergoing colorectal surgery by involving patients, carers and staff in: (1) identifying perceived value and value deficits for enhanced recovery; (2) modifying current education practices to address educational deficits; and (3) evaluating these changes for preparing patients to enhance their recovery." | Several: Patients, family members, health-care providers (patients undergoing colorectal surgery for cancer, carers and staff) | action research | Observations, individual interviews and focus group interviews, and questionnaires | 12 weeks | 138 | 15 obnservations, 60 patient quistionnaires, 60 individual interviews, and 3 focus group interviews |
| Pope, C., McKenna, G., Turnbull, J., Prichard, J., & Rogers, A. (2019). Navigating and making sense of urgent and emergency care processes and provision. Health Expectations, 22(3), 435-443. | UK | "...to explore how people make sense of urgent care provision and processes, and how this impacts on their navigation of services." | Patients (regular users of emergency care, potentially marginalized users, and people from East/Central Europé) | not described | Individual interviews | 6 to 12 months | 93 | 134 interviews |
| Porter, T., Ong, B. N., & Sanders, T. (first published 2019). Living with multimorbidity? The lived experience of multiple chronic conditions in later life. Health, 2020, 24(6), 701-718. | UK | "...to understand how older people living with multiple chronic conditions make sense of illness. Our aim is to provide a foundational reading of multiple chronic conditions, beginning with the premise that medical diagnoses do not a priori determine illness." | Patients (older people living with multiple conditions and participant spouses) | Phenomenology, Grounded theory & qualitative longitudinal research | Individual interviews | 3 to 6 months | 15 | 27 interviews |
| Principi, A., Smeaton, D., Cahill, K., Santini, S., Barnes, H., & Socci, M. (first published 2018). What happens to retirement plans, and does this affect retirement satisfaction? International Journal of Aging & Human Development, 2020, 90(2) 152–175 | UK, Italy, and US | "This study examines the role of planning and plan fulfillment for retirement satisfaction using a dynamic resource theory approach." | People in the community (older workers) | Qualitative longitudinal research | Individual interviews | about 2 years | 111 | 217 interviews |
| Pyörälä, E., Mäenpää, S., Heinonen, L., Folger, D., Masalin, T., & Hervonen, H. (2019). The art of note taking with mobile devices in medical education. BMC Medical Education, 19(1), 96-96. | Finland | "...to explore students’ perceptions of the study use of mobile devices and digital note taking practices in the first cohort of tablet computer users during their studies. 1) What were the students’ most important self-reported study uses of mobile devices? 2) How did the note taking practices change over the study years? 3) What were the students’ perceptions of the best practices of note taking with mobile devices?" | Students (medical and dental students) | Action research | Online questionnaires including open-ended questions, and focus-group interviews | about 5 years | 176 | 2000 short text descriptions, 2 focus group interviews. |
| Ralph, A. F., Butow, P., Craig, J. C., Wong, G., Chadban, S. J., Luxton, G., Gutman, T., Hanson, C. S., Ju, A., & Tong, A. (2019). Living kidney donor and recipient perspectives on their relationship: Longitudinal semi-structured interviews. BMJ Open, 9(4), e026629-e026629. | Australia | "The aim of this study is to collect longitudinal data on donor and recipient expectations and perspectives of their relationship in living kidney donor transplantation, which may inform strategies to mitigate risks of relationship tension and conflict and support relationship resilience, thereby contributing to improved outcomes in living kidney donor transplantation" | Several: Patients and family members (living kidney donors and their recipients) | Grounded theory | Individual interviews | 13 to 15 months | 32 | 61 interviews |
| Ramanaik, S., Collumbien, M., Prakash, R., Howard-Merrill, L., Thalinja, R., Javalkar, P., Murthy, S., Cislaghi, B., Beattie, T., Isac, S., Moses, S., Heise, L., & Bhattacharjee, P. (2018). Education, poverty and "purity" in the context of adolescent girls' secondary school retention and dropout: A qualitative study from Karnataka, southern India. Plos One, 13(9), e0202470-e0202470. | India | "...to investigate gender socialisation [...] We analyse how gender-related norms interact with poverty and family background to result in girls’ drop out from secondary school. In addition, we identify the main facilitators of school retention and modifications to gender performance." | People in the community (adolescent girls) | Case study | Individual interviews | 16 months | 36 | 66 interviews |
| Reed, E., Todd, J., Lawton, S., Grant, R., Sadler, C., Berg, J., Lucas, C., & Watson, M. (2018). A multi-professional educational intervention to improve and sustain respondents' confidence to deliver palliative care: A mixed-methods study. Palliative Medicine, 32(2), 571-580. | UK | "Research question: "Can a multi-professional palliative care education intervention improve and sustain candidates’ confidence to deliver palliative care?" Aims: "•To evaluate the impact of the ECEPC on candidates’ confidence in palliative care; •To determine whether this is sustained over time; •To explore the candidate’s perception of the influence of the course on their confidence in practice." | Health care providers (candidates undertaking the ECEPC educational intervention) | mixed methods | Individual interviews and questionnairs | 6 months | 112 answered survey, 15 particiopated in interviews | Unclear |
| Rehackova, L., Araújo-Soares, V., Steven, S., Adamson, A. J., Taylor, R., & Sniehotta, F. F. (first published 2019). Behaviour change during dietary Type 2 diabetes remission: A longitudinal qualitative evaluation of an intervention using a very low energy diet. Diabetic Medicine, 2020, 37, 953–962. | UK | "To understand the process of behaviour change through the experiences of people with Type 2 diabetes engaged in an 8-month diabetes remission intervention including a 2-month weight loss phase with the use of a very low energy diet (VLED), and a 6-month, structured weight maintenance phase." | Patients (people with type 2 diabetes) | not described | Individual interviews | 8 months | 11 | 33 interviews |
| Richter Sundberg, L., Garvare, R., & Nyström, M. E. (2017). Reaching beyond the review of research evidence: a qualitative study of decision making during the development of clinical practice guidelines for disease prevention in healthcare. BMC Health Services Research, 17(1), 344-344. | Sweden | "Our focus is on the judgement and decision making process of the Prioritization group in the third phase of the NBHW guideline development model, i.e. prioritizing and deciding on guideline recommendations. Accordingly, the aim of this study was to investigate the bases for decisions and the decision making process of the Prioritization group during development of clinical guidelines with a disease preventive scope in Sweden. Three more specific research questions were posed: I. Which decision making criteria were used, and how did research evidence influence the Prioritization group’s judgment and decision making process? II. Did the composition of decision criteria change over time, and if so, how? III. Did the Prioritization group encounter conflicts or dilemmas during judgement and decision making? If so, on what subjects and how were these conflicts or dilemmas managed?" | Policy-makers (experts in preventive guidelines) | case study | Observations, open ended questions in questionnaris, and policy documents. | 3 years | 25 | 482 pages of notes from observations, 62 questionnaires and documents (total of 89 pages) |
| Ridder, H.-G., & Schrader, J. S. (2017). Processing of intended and unintended strategic issues and integration into the strategic agenda. Health Care Management Review, 44(4), 332-343. | Germany | "Hence, we ask how intended strategic issues are processed into deliberate strategies and how unintended strategic issues are processed into emergent strategies. Finally, we ask how deliberate and emergent strategies are integrated into the strategic agenda of a hospital." | Policy-makers (members of a hospital board) | Case study | Observations, documents (e.g., official protocols, internal and external documents), and individual interviews | 2 years | 13 | 13 interviews, 37 observation sessions (65 hours), 37 meeting protocols, 34 official documents, 86 newspaper articles |
| Riegel, B., Dickson, V. V., Garcia, L. E., Masterson Creber, R., & Streur, M. (2017). Mechanisms of change in self-care in adults with heart failure receiving a tailored, motivational interviewing intervention. Patient Education and Counseling, 100(2), 283-288. | US | "The aim of this study was to identify the mechanism of intervention effectiveness by elucidating the MI [motivational interviewing] techniques used and the relationship between the techniques and changes in self-care. Combined with our prior pilot work, answering these aims will allow us to develop hypotheses about mechanisms of effectiveness, which can be tested in later studies." | Patients (patients with heart failure) | mixed methods | Questionnairs, observations (e.g., recorded sessions with motivational interviewing) | 90 days | 8 | unclear |
| Robards, F., Kang, M., Steinbeck, K., Hawke, C., Jan, S., Sanci, L., Liew, Y. Y., Kong, M., & Usherwood, T. (2019). Health care equity and access for marginalised young people: A longitudinal qualitative study exploring health system navigation in Australia. International Journal for Equity in Health, 18(1), 41-41. | Australia | "This longitudinal study explored young people’s journeys through the health system in New South Wales (NSW), Australia, over time. The aim was to understand health system navigation, including the use of technology, for young people belonging to one or more marginalized groups." | People in the community (marginalised young people aged 12-24 years) | Grounded theory & qualitative longitudinal research | Individual interviews | Up to 12 months | 41 | 136 interviews |
| Roberts, D., Calman, L., Large, P., Appleton, L., Grande, G., Lloyd‐Williams, M., & Walshe, C. (2018). A revised model for coping with advanced cancer. Mapping concepts from a longitudinal qualitative study of patients and carers coping with advanced cancer onto Folkman and Greer's theoretical model of appraisal and coping. Psycho-Oncology, 27(1), 229-235. | UK | "Data from a study on coping with advanced cancer are used to explore whether the Folkman and Greer model reflects the coping processes participants used. These data are from a serial interview study designed to answer the following questions: a. What do people do to cope well when living with advanced cancer? b. Why and when do they perceive these coping strategies as effective? c. How can health care professionals support effective coping strategies?" | Patients (people with advanced cancer) | Qualitative longitudinal research | individual interviews | 4 to 12 weeks | 26 | 45 interveiws |
| Robinson, J., Gott, M., Gardiner, C., & Ingleton, C. (2018). The impact of the environment on patient experiences of hospital admissions in palliative care. BMJ Supportive & Palliative Care, 8(4), 485-492. | New Zealand | "...exploring the impact of the environment on experiences of hospitalizations from the patients͛ perspective." | Patients (patients who met one of the Gold Standard Framework Prognostic Indicators for palliative care need) | Critical realism | Individual interviews | Unclear, probably a few days up to a few weeks | 14 | 26 intervews |
| Rodriguez-Morales, L. (2017). In your own skin: The experience of early recovery from alcohol-use disorder in 12-step fellowships. Alcoholism Treatment Quarterly, 35(4), 372-394. | UK | "The study reported here adopts a longitudinal-single case approach to examine a young adult’s experience of early recovery in 12-Step fellowships. It attempts to understand what it is like to recover from an AUD [Alcohol-Use Disorder] in all its social and personal complexity, while providing an in-depth exploration of the individual psychological transformation." | People in the community (young adults) | case study & qualitative longitudinal research | individual interviews | 8 months | 1 | 3 interviews |
| Rosen, J. G., Clermont, A., Kodish, S. R., Matar Seck, A., Salifou, A., Grais, R. F., & Isanaka, S. (2018). Determinants of dietary practices during pregnancy: A longitudinal qualitative study in Niger. Maternal & Child Nutrition, 14(4), 1-1. | Niger | "This paper presents findings from a longitudinal qualitative study in south‐central Niger exploring maternal food consumption practices and their underlying determinants during pregnancy." | Several: people in the community, familymembers and health-care providers (pregnant women, household members, and health workers) | Grounded theory & qualitative longitudinal research | Individual interviews, and focus group interviews | 5 months | 140 | 153 individual interviews, and 38 focus group interviews |
| Rosenberg, A., Heimer, R., Keene, D. E., Groves, A. K., & Blankenship, K. M. (2019). Drug treatment accessed through the criminal justice system: Participants' perspectives and uses. Journal of Urban Health, 96(3), 390-399. | US | "Given the existing literature on the treatment of addiction, we focus on non-addictionrelated narratives to understand the broader role drug treatment plays in the lives of justice-involved people." | People in the community (participants who were recently released from prison or jail and convicted of a non-violent drug-related crime) | not described | Individual interviews | about 2 years | 45 | 235 |
| Ross, V., Kõlves, K., Kunde, L., & De Leo, D. (2018). Parents' experiences of suicide-bereavement: A qualitative study at 6 and 12 months after loss. International Journal of Environmental Research and Public Health, 15(4). | Australia | "...to examine the individual experiences of both mothers and fathers bereaved by suicide over time, specifically at the six month and 12 month time points after the death of their child." | Family members (mothers and fathers bereaved by suicide) | not described | Individual interviews | 6 months | 14 | 28 interviews |
| Rosser, E. A., Scammell, J., Heaslip, V., White, S., Phillips, J., Cooper, K., Donaldson, I., & Hemingway, A. (2019). Caring values in undergraduate nurse students: A qualitative longtitudinal study. Nurse Education Today, 77, 65-70. | UK | "...the purpose of this paper is to report on the final phase of a five-phase case study which uses a prospective qualitative longitudinal approach to understand the beliefs and values of caring, held by student nurses from the day of entry through their education programme to completion" | Students (nursing students) | Case study & Qualitative longitudinal research | Individual interviews and focus group interviews | about 3 years | 14 | Unclear. |
| Rulifson, G., & Bielefeldt, A. R. (2019). Evolution of students' varied conceptualizations about socially responsible engineering: A four year longitudinal study. Science and Engineering Ethics, 25(3), 939-974. | US | "This study aims to develop a better understanding of how the college experience influences students’ ideas about SRE [socially responsible engineering]. Weidman’s updated Inputs–Environment– Outputs (I–E–O) model of undergraduate socialization (Weidman was used as framework). RQ1: How did students’ pre-college experiences impact their views of socially responsible engineering? RQ2: What are the main influences that shaped evolving ideas about socially responsible engineering during students’ 4 years of college? RQ3: How do undergraduate engineering students change in the ways that they understand socially responsible engineering during college?" | Students (engineering students) | not described | Individual interviews and questionnairs | About 3 years | 21 | Probably 63 interviews |
| Ryba, T. V., Stambulova, N. B., Selänne, H., Aunola, K., & Nurmi, J.-E. (2017). “Sport has always been first for me” but “all my free time is spent doing homework”: Dual career styles in late adolescence. Psychology of Sport & Exercise, 33, 131-140. | Finland | "In this research, we conceptualised dual career as a story that young people tell about their engagement at sport and school (see also Savickas, 2011) to examine three research questions: (a) How and to what extent do adolescent Finnish athletes narrate and integrate their autobiographical events in sport and education into identity narrative?, (b) How and to what extent are sport and education integrated in the adolescent athletes' projected future?, and (c) What does the relationship between one's narratives ofthe past and narratives ofthe future reveal about their dual career style?" | People in the community (young athletes) | Qualitative longitudinal research | individual interviews | 6 months | 18 | Unclear |
| Sarkar, D., Murphy, H., Fisseha, T., Koroma, A. S., Hodges, M. H., Adero, N., Ngalombi, S., Nabakooza, J., Wun, J., & Namaste, S. M. L. (2018). Understanding the process of strengthening multi-sectoral efforts for anemia reduction: Qualitative findings from Sierra Leone and Uganda. The International Journal of Health Planning and Management, 33(4), 1024-1044. | Sierra Leone and Uganda | "...explore country experiences developing a multi‐sectoral anemia platform and strategyIn this paper, we present key findings and lessons learned from SPRING's documentation in Sierra Leone and Uganda, along with similarities and variations across the 2 settings, to inform future global and country multi‐sectoral anemia planning efforts." | Policy-makers (NAWG-members) | Case study | Individual interviews | 11 to 24 months (differed between sites) | 25 | 43 interviews |
| Schiltz, J., Derluyn, I., Vanderplasschen, W., & Vindevogel, S. (2019). Resilient and self‐reliant life: South Sudanese refugees imagining futures in the adjumani refugee setting, Uganda. Children & Society, 33(1), 39-52. | Uganda | "This article analyses how South Sudanese refugee youngsters in Uganda imagine and act towards their futures in a humanitarian space that aims for refugees to become resilient and self-reliant. […] In examining what happens when youngsters become acquainted with the refugee policy and the options for the future that are available to them and explores what such policies can mean within the permanent temporariness of the camp." | People in the community (young South Sudanese refugees) | the methodological approach of bricolage & qualitative longitudinal research | Individual interwiews, informal conversations, and observations | about 2 years | 30 | Unclear, 24 participants were interviewed at least three out of the four times |
| Schmid-Mohler, G., Caress, A.-L., Spirig, R., Benden, C., & Yorke, J. (2019). "Thrust out of normality"-How adults living with cystic fibrosis experience pulmonary exacerbations: A qualitative study. Journal of Clinical Nursing, 28(1), 190-200. | Switzerland | "The aim of this study was to explore the experience of adults with CF[cystic fibrosis] during a pulmonary exacerbation over time." | Patients (people with Cystic Fibrosis) | not described | Individual interviews and field notes | 3-4 weeks | 18 | 31 interviews |
| Schröder, S. L., Fink, A., & Richter, M. (2018). Socioeconomic differences in experiences with treatment of coronary heart disease: A qualitative study from the perspective of elderly patients. BMJ Open, 8(11), e024151-e024151. | Germany | "...to identify socioeconomic differences in the patient’s perspective and their experiences with the treatment pathway for CHD [coronary heart disease] in all sectors from therapy to aftercare." | Patients (patients with a confirmed diagnosis of CHD) | Grounded theory & qualitative longitudinal research | Individual interviews | 6 months | 41 | 58 interviews |
| Serholt, S. (2018). Breakdowns in children's interactions with a robotic tutor: A longitudinal study. Computers in Human Behavior, 81, 250-264. | Sweden | "The aim of this paper is to explore the challenges that currently exist when moving robotic tutors into actual classrooms by focusing specifically on breakdowns in children's interactions with a robotic tutor at their school. Video recordings of such instances are analyzed in-depth through qualitative methods, guided by the following research question: What causes break-downs in children's interactions with a robotic tutor, and what consequences do such breakdowns pose for the educational use of robots?" | Students (school children) | not described | Observations, brief informal interviews, and questionnaires | 13 weeks | 6 | about 14 hours of video recorded observations |
| Sestito, S. F., Rodriguez, K. L., Saba, S. K., Conley, J. W., Mitchell, M. A., & Gordon, A. J. (2017). Homeless veterans’ experiences with substance use, recovery, and treatment through photo elicitation. Substance Abuse, 38(4), 422-431. | US | "We conducted a secondary qualitative analysis aimed at describing H-PACT veterans’ experiences with substance use (SU), substance use recovery (SUR), and substance use treatment (SUT)." | People in the community (homeless veterans) | not described | Individual interviews, and photovoice | 1 month | 15 | 25 interviews |
| Shelton, R. C., Charles, T.-A., Dunston, S. K., Jandorf, L., & Erwin, D. O. (2017). Advancing understanding of the sustainability of lay health advisor (LHA) programs for African-American women in community settings. Translational Behavioral Medicine, 7(3), 415-426. | US | "...to understand factors that influence the long-term implementation, sustainability, and impact of this community-engaged LHA [lay health advisor] program within African- American communities. This paper seeks to (1) advance understanding of barriers and facilitators to the long term implementation and sustainability of community-engaged and community-based LHA programs, including factors that impact the participation and retention of LHAs, and (2) document the impact of LHA programs on women who serve as LHAs and more broadly in African-American communities." "The goal of the qualitative data, presented here, was to inform a more in-depth and comprehensive understanding of factors that influence long-term NWP [National Witness Project] program implementation and sustainability, and to increase the likelihood we had identified the full range of factors, including organizational and contextual factors that were not measured in the quantitative study." | Health care providers (African-American lay health advisors) | mixed methods | individual interviews | 12 to 18 month | 76 | 144 interveiws |
| SmithBattle, L. (2019). Housing trajectories of teen mothers and their families over 28 years. The American Journal of Orthopsychiatry, 89(2), 258-267. | US | "The specific aims of this substudy were to describe the housing trajectories of teen mothers over 28 years and to explore how their housing trajectories were shaped by family resources, housing programs, and discrimination." | Several: People in the community and family members (teen mothers and their family) | Phenomenology & qualitative longitudinal research | Individual interviews | 28 years | 18 | Unclear |
| Solomon, P., O'Brien, K. K., Nixon, S., Letts, L., Baxter, L., & Gervais, N. (2018a). Qualitative longitudinal study of episodic disability experiences of older women living with HIV in Ontario, Canada. BMJ Open, 8(4), e021507-e021507. | Canada | "...to examine the disability experiences of older women living with HIV over time." | Patients (women aged 50 years or older living with HIV for more than 6 years) | Qualitative longitudinal research | Individual interviews | 20 months | 10 | 40 interviews |
| Solomon, P., O'Brien, K. K., Nixon, S., Letts, L., Baxter, L., & Gervais, N. (2018b). Trajectories of episodic disability in people aging with HIV: A longitudinal qualitative study. Journal Of The International Association of Providers of AIDS Care, 17, 1-7. | Canada | "The purpose of this study was to understand how the episodic nature of HIV and the associated uncertainty shape the disability experience of adults aging with HIV over time." | Patients (older adults living with HIV) | Qualitative longitudinal research | Individual interviews | 20 months | 24 | 96 interviews |
| Stapley, E., Target, M., & Midgley, N. (2017). The journey through and beyond mental health services in the United Kingdom: A typology of parents' ways of managing the crisis of their teenage child's depression. Journal Of Clinical Psychology, 73(10), 1429-1441. | UK | "...exploring the patterns or ideal types of parental experiences that could exist over time among parents of adolescents diagnosed with depression. Specifically, our study sought to create a typology of parents’ ways of managing and dealing with the crisis of their teenage child’s depression over a 2-year period, starting at the point of their child’s referral to CAMHS [child and adolescent mental health services] in the United Kingdom and diagnosis of depression, and then after their child had received treatment at CAMHS, and finally one year later." | Family members (parents of adolescents diagnosed with depression) | not described | individual intewrview | about one year | 33 | 85 interview |
| Stephens, G. C., Rees, C. E., & Lazarus, M. D. (2019). How does donor dissection influence medical students' perceptions of ethics? A cross-sectional and longitudinal qualitative study. Anatomical Sciences Education, 12(4), 332-348. | Australia | "This research aimed to identify any areas of interplay between anatomy and medical ethics curricula to ultimately inform development of an evidence-based integrated anatomy and bioethics curriculum. The following exploratory research question was addressed: What are the impacts (if any) of donor dissection on students’ perceptions of medical ethics?" | Students (medical students) | Qualitative longitudinal research | Data from online discussion forums, individual interviews and focus group interviews | 18 months | 207 | Discussion forums including 51,024 words. 11 interviews. |
| Stevens, M. (2018). Parents' experiences of services addressing parenting of children considered at-risk for future antisocial and criminal behaviour: A qualitative longitudinal study. Children & Youth Services Review, 95, 183-190. | UK | "The study aimed to explore the ways in which families with children identified by services as at risk of future criminal or antisocial behaviour benefit, or do not, from services' intervention, in the longer term. focussing on the perspective of parents, while also including the perspectives of practitioners working [...] The broad objectives were as follows: To explore with primary carers their experiences of interactions with services, and with practitioners their experiences of working with the families • To understand which aspects of families' lives participants felt best responded to service provision • To understand which aspects of service provision were viewed as helpful or unhelpful by primary carers and practitioners." | Several: Family members and health-care providers (high need families and practitioners) | Qualitative longitudinal research | Individual interviews | about 5 years | 32 | Unclear |
| Storholm, E. D., Ober, A. J., Hunter, S. B., Becker, K. M., Iyiewuare, P. O., Pham, C., & Watkins, K. E. (2017). Barriers to integrating the continuum of care for opioid and alcohol use disorders in primary care: A qualitative longitudinal study. Journal of Substance Abuse Treatment, 83, 45-54. | US | "The objective of this study was to use an organizational capacity framework to examine perceived barriers to implementing the continuum of care for OAUD [opioid and alcohol use disorders] in a community-based primary care organization over three time points: pre-implementation (preparation), early implementation (practice), and full implementation." | Health care providers (clinic administrators and medical and mental health providers) | Qualitative longitudinal research | individual interviews, and focus group interviews | about 3 years | unclear, probabvly 72 | unclear, about 48 individual interviews, and 6 focus group interviews |
| Sukhera, J., Milne, A., Teunissen, P. W., Lingard, L., & Watling, C. (2018). Adaptive reinventing: implicit bias and the co-construction of social change. Advances in Health Sciences Education, 23(3), 587-599. | Canada | "...to explore how individuals and workplace learning environments influence each other to produce change following a learning activity that disrupts workplace norms. We hoped to draw out how the process of bringing implicit biases into conscious awareness influenced workplace learning and how implicit bias recognition and management could potentially shape workplace transformation." | Health care providers (physicians and nurses) | Case study and grounded theory | Individual interviews | 12 months | 14 | 32 interviews |
| Superdock, A., K., Barfield, R. C., Brandon, D. H., & Docherty, S. L. (2018). Exploring the vagueness of Religion & Spirituality in complex pediatric decision-making: a qualitative study. BMC Palliative Care, 17(1), 107-107. | US | "...to illuminate the influence of R&S [Religion & Spirituality] on parental decision-making and explore how providers interact with parents for whom R&S are important." | Several: Patients, family members and health-care providers (parents and health care providers of infants with complex life-threatening conditions) | Case study & qualitative longitudinal research | Individual interviews, field notes, questionnaires, and data from medical charts | Between 8 days and 531 days (median = 380 days, mean = 324 days, SD = 174 days) | 136 | 363 intervews |
| Sussman, T., & Orav-Lakaski, B. (first published 2018). "I didn't even make my bed": Hospital relocations and resident adjustment in long-term care over time. The Gerontologist, 2020, 60(1), 32–40. | Canada | "... experiences with the relocation process to Long Term Care from hospital over time. More specifically, the study sought to: (a) describe the relocation trajectories of older adults moving to LTC [long term care] from hospital and (b) identify the conditions that help or hinder older adults’ capacities to adjust to LTC when relocating in the context of multiple moves." | People in the community (older adults) | Grounded theory & qualitative longitudinal research | Individual interviews | up to 2 years | 9 | 24 intervews |
| Tafvelin, S., Isaksson, K., & Westerberg, K. (2018). The first year of service: A longitudinal study of organisational antecedents of transformational leadership in the social service organisations. British Journal of Social Work, 48(2), 430-448. | Sweden | "The purpose of this longitudinal interview study is to explore organisational antecedents to leadership behaviour among first line managers in social service organisations. Using transformational leadership theory as a framework, we first investigate what kind of leadership the managers are striving to achieve in terms of leadership ideals in their first year of service. By following managers over time, we then aim to capture organisational and working conditions that may hinder managers from performing this leadership ideal in the realm of their everyday life." | Managers (first line managers in social service organisations) | not described | individual interviews | 12 months | 8 | 20 interviews |
| Taylor, R. D., Carson, H. J., & Collins, D. (2018). The impact of siblings during talent development: A longitudinal examination in sport. Journal of Applied Sport Psychology, 30(3), 272-287. | UK | "Therefore, a 1-year longitudinal study was conducted to examine the impact of siblings on TD [talent development] where both are prevalent within the talent pathway. Interviews were conducted with siblings and parents with the aim of further enhancing our knowledge of the impact of this subsystem on the development process." | Family members (siblings and parents) | case study | Joint interviews | one year | 13 | 44 interviews |
| Ten Hoeve, Y., Kunnen, S., Brouwer, J., & Roodbol, P. F. (2018). The voice of nurses: Novice nurses' first experiences in a clinical setting. A longitudinal diary study. Journal of Clinical Nursing, 27(7), e1612-e1626. | The Netherlands | "The current longitudinal study has an open character and focuses on novice nurses’ lived experiences in a clinical setting to gain a greater understanding of which demands from personal and professional life they are confronted with, and what can be done to improve the transition from being a novice to a professional staff nurse. With lived experiences, we refer to the description of and reflections on weekly experiences of novice nurses during their first 2 years on a clinical ward after graduation as a nurse" | Health care providers (novice nurses) | phenomenology & qualitative longitudinal research | diaries | 2 years | 18 | 580 diary entries |
| Testoni, I., Francescon, E., De Leo, D., Santini, A., & Zamperini, A. (2019). Forgiveness and blame among suicide survivors: A qualitative analysis on reports of 4-year self-help-group meetings. Community Mental Health Journal, 55(2), 360-368. | Italy | "The main objective of this research was to verify whether the forgiveness process spontaneously appear in a self-help group composed by suicide survivors and how it matches with blame processes. In fact, following the perspective of Supiano (2012) and of Lee et al. (2015), we wanted to analyze how the different types of causal attributions, self or other blame and responsibility, intervene, and how these explanations are intertwined with forgiveness processes. Secondarily, we wanted to check the effects of the self-help group intervention, analysing if the participants had an elaboration and/or a decrease of the feelings of self/other blame and an increase of forgiveness and acceptance of the relative’s suicide." | Patients (participants in self-help groups for suicide survivors) | not described | Reports of narrations of the support group meetings. | Up to 3 years | 10 | Unclear. |
| Theadom, A., Rutherford, S., Kent, B., & McPherson, K. (2019). The process of adjustment over time following stroke: A longitudinal qualitative study. Neuropsychological Rehabilitation, 29(9), 1464-1474. | New Zealand | "This study aimed to explore how people adjust to life after stroke over time and to identify what helps or hinders recovery." | Patients (people experienced a stroke) | qualitative description methodology & qualitative longitudinal research | Individual interviews | 36 months | 82 | 184 interviews |
| Theron, L., & van Rensburg, A. (2018). Resilience over time: Learning from school-attending adolescents living in conditions of structural inequality. Journal of Adolescence, 67, 167-178. | South Africa | "Thus, the questions directing the study we report in this article were: Do the same (or different) resilience-enabling resources inform township-dwelling, school-attending adolescents' resilience accounts when they are invited to self-explain their resilience at two distinct points in time? Which resilience-enabling resources, if any, become significantly more (or less) salient over time and how do township-dwelling, school-attending adolescents explain the resilience-enabling value of these resources?" | Students (school-attending adolescents) | Phenomenology & qualitative longitudinal research | Drawings combined with written reflections | 24 months | 140 | 280 drawings and written reflections |
| Thomson, R., Martin, J. L., & Sharples, S. (2017). The experience of couples being given an oxygen concentrator to use at home: A longitudinal interpretative phenomenological analysis. Journal of Health Psychology, 22(6), 798-810. | UK | "...the aim of this study was to follow couples and describe their experience as they began LTOT [Long-term oxygen therapy] to understand the issues associated with the device and how these may alter over time." | Several: Patients and family members (couples were one had COPD and oxygene at home) | phenomenology | Joint interviews | 5 months | 8 | 11 interviews |
| Thorpe, G., & McArthur, M. (2017). Social adaptation following intestinal stoma formation in people living at home: A longitudinal phenomenological study. Disability & Rehabilitation, 39(22), 2286-2293. | UK | "...this paper explores the outward focus on a disrupted social world, highlighting how stoma-forming surgery impacts on individuals’ abilities to participate and interact socially and how these experiences can change over time." | Patients (individuals having had stoma-forming surgery) | phenomenology | Individual interviews | 15 months | 12 | 36 interviews |
| Todis, B., McCart, M., & Glang, A. (2018). Hospital to school transition following traumatic brain injury: A qualitative longitudinal study. Neurorehabilitation, 42(3), 269-276. | US | "Our objective was to better understand the complex variables that influence the hospital–school transition experience by analyzing parent and teacher experiences, impressions, and interactions." | Several; Family members and teachers (parents and teachers) | Qualitative longitudinal research | Individual interviews and observations | about 4 years (range 0-6) | 71 | 162 interviews, 30 observations |
| Toly, V. B., Blanchette, J. E., Alhamed, A., & Musil, C. M. (2019). Mothers' voices related to caregiving: The transition of a technology-dependent infant from the NICU to home. Neonatal Network, 38(2), 69-79. | US | "is to explore how mothers of technology-dependent infants perceive their transition experiences just prior to and during the first months after their infants’ initial discharge from the NICU to home" | Family members (mothers caring for an infant expected to be discharged home within two to three weeks for the first time dependent on medical technology) | Qualitative longitudinal research | Individual interviews | about 3 months | 19 | 28 interviews |
| Tornero, M. D. L. A., & Capella, C. (2017). Change during psychotherapy through sand play tray in children that have been sexually abused. Frontiers in Psychology, 8, 617-617. | Chile | "The present study therefore seeks to analyze characteristics of play during therapy among child victims of sexual assault between the ages of 7 and 10. Three particular moments of the therapeutic process are observed using sand tray therapy." | People in the community (child victims of sexual assault between the ages of 7 and 10) | not described | observations | 6 months | 7 | 19 observations |
| Tsai, A., C., Hatcher, A. M., Bukusi, E. A., Weke, E., Lemus Hufstedler, L., Dworkin, S. L., Kodish, S., Cohen, C. R., & Weiser, S. D. (2017). A livelihood intervention to reduce the stigma of HIV in rural Kenya: Longitudinal qualitative study. AIDS And Behavior, 21(1), 248-260. | Kenya | "...we conducted a qualitative study to explore longitudinal changes in stigma among persons with HIV participating in a randomized controlled trial in rural Kenya. Treatment-arm participants received a livelihood intervention, called Shamba Maisha (meaning ‘‘farming life’’ in Kiswahili), and our goal was to explore the intervention’s perceived impacts on HIV stigma and to understand the mechanisms through which any stigma-related changes may have occurred." | Patients (persons with HIV participating in a randomized controlled trial) | Qualitative longitudinal research | Individual interviews | 6 months | 54 | unclear |
| Tse, S., Mak, W. W. S., Lo, I. W. K., Liu, L. L., Yuen, W. W. Y., Yauc, S., Hod, K., Chan, S.-K., & Wong, S. (2017). A one-year longitudinal qualitative study of peer support services in a non-Western context: The perspectives of peer support workers, service users, and co-workers. Psychiatry Research, 255, 27-35. | Hong Kong | "The present paper aims to identify the changes in the perceptions (concerning peer support services and their key ingredients) of PSWs [Peer support workers] among themselves, their co-workers (supervisors, mentors of the PSWs, and non-peer professional staff members), and service users at different community-based mental healthcare centers in Hong Kong over a 12 month period." | Several: health-care providers, peer-supporters, tutors, patients (peer support workers, supervisors, mentors of the peer support workers, non-peer professional staff members, and service users) | Qualitative longitudinal research | Individual interviews | 12 months | 14 | 77 interviews |
| Tudor-Sfetea, C., Rabee, R., Najim, M., Amin, N., Chadha, M., Jain, M., Karia, K., Kothari, V., Patel, T., Suseeharan, M., Ahmed, M., Sherwani, Y., Siddiqui, S., Lin. Y., & Eisingerich, A. B. (2018). Evaluation of Two Mobile Health Apps in the Context of Smoking Cessation: Qualitative Study of Cognitive Behavioral Therapy (CBT) Versus Non-CBT-Based Digital Solutions. JMIR Mhealth and Uhealth, 6(4), e98. | UK | "The purpose of this study was, therefore, first, to explore users’ perceptions of two mHealth apps, one CBT-based [cognitive behavioral therapy] app, Quit Genius (QG), and one non-CBT-based app, National Health Service (NHS) Smokefree (SF), over a variety of critical themes. Second, the study also sought to investigate the perceptions and health behavior with respect to smoking cessation for users of each app." | People in the community (smoker who intends to quit) | Qualitative longitudinal research | Individual interviews | 1 week | 29 | 58 interviews |
| Turnbull, J., Pope, C., Prichard, J., McKenna, G., & Rogers, A. (2019). A conceptual model of urgent care sense-making and help-seeking: a qualitative interview study of urgent care users in England. BMC Health Services Research, 19(1), 481. | UK | "Our aim is to explicate how people make sense of urgent care through the work that they do when they experience a health problem, and in turn how this drives action e.g. seeking help from services or choosing not to seek help." | People in the community (people seeking or not seeking health care) | not described | Individual interviews | 12 months | 100 | 141 interviews |
| Vaghefi, I., & Tulu, B. (2019). The continued use of mobile health apps: Insights from a longitudinal study. JMIR Mhealth and Uhealth, 7(8), e12983. | US | "To understand continued use of mHealth apps and individuals’ decisions related tothis behavior." | Students (university students) | Qualitative longitudinal research | Individual interviews and diaries | 2 weeks | 17 | 34 interviews, 193 diary entries (over 300 pages) |
| Valdez, A., Nowotny, K. M., Zhao, Q.-W., & Cepeda, A. (2019). Interpersonal partner relationships, bonds to children, and informal social control among persistent male offenders. Social Problems, 66(3), 468-483. | US | "We explore a qualitative examination of the men’s motivations, aspirations, and goals to lead conventional lives and desist from criminal behaviors." | People in the community (young men who were gang affiliated as adolescents) | Ethnography | Individual interviews, and field observations | one month | 40 | Unclear |
| Van Natta, M., Burke, N. J., Yen, I. H., Rubin, S., Fleming, M. D., Thompson‐Lastad, A., & Shim, J. K. (2018). Complex care and contradictions of choice in the safety net. Sociology of Health & Illness, 40(3), 538-551. | US | "This article explores the complicated and often-contradictory notions of choice at play in complex care management (CCM) programmes in the US healthcare safety net." | Several: Patient and health care providers (patients and providers in the clinic, patients’ homes, and other settings) | ethnography | Individual interviews and observations | about 2 years | 108 | Over 1,000 hours of observations, 185 interviews |
| Vandenberg, B. E., Advocat, J., Hassed, C., Hester, J., Enticott, J., & Russell, G. (first published 2018). Mindfulness-based lifestyle programs for the self-management of Parkinson's disease in Australia. Health Promotion International, 2019, 34, 668–676. | Australia | "To characterize the experiences of communityliving adults with Stage 2 PD [Parkinson's disease] participating in a facilitated, group mindfulness-based lifestyle program, and explore how the program influenced beliefs about self-management of their disease." | Patients (adult participants with Parkinson's disease participating in a mindfuldness program) | Qualitative longitudinal research | Individual interviews | 8 months | 16 | 37 interviews |
| VanHeuvelen, J. S. (2019). Isolation or interaction: Healthcare provider experience of design change. Sociology of Health & Illness, 41(4), 692-708. | US | "To assess the experience of providers, I examine ethnographic and interview data gathered at ‘Children’s NICU’ as it moved from an open-bay to single-patient rooms." | Health care providers (health-care providers at children NICU) | Ethnography | Individual interviews, observations and field notes | 13 months | 40 | Unclear |
| Varagona, L. M., & Hold, J. L. (2019). Nursing students' perceptions of faculty trustworthiness: Thematic analysis of a longitudinal study. Nurse Education Today, 72, 27-31. | US | "The purpose of this study was to explore nursing students' perceptions of faculty trustworthiness. The study sought to identify characteristics and behaviors of nursing faculty that lead nursing students to trust them." | Students (nursing students) | Qualitative longitudinal research | Focus group interviews | 2 years | 77 | 11 focus group interviews |
| Vassilev, I., Rogers, A., Kennedy, A., Oatley, C., & James, E. (2019). Identifying the processes of change and engagement from using a social network intervention for people with long‐term conditions. A qualitative study. Health Expectations, 22(2), 173-182. | UK | "An acceptable intervention which facilitates self- management by mobilizing network support and improves network engagement has a positive impact on health and quality of life. This study aims to identify the processes through which such changes and engagement take place" (from abstract) | Patients (people living with long-term conditions) | Case study | Observations, and individual intervews | 3 months | 15 | Unclear |
| Vestergren, S., Drury, J., & Chiriac, E. H. (2018). How collective action produces psychological change and how that change endures over time: A case study of an environmental campaign. British Journal of Social Psychology, 57(4), 855-877. | Sweden | "The present research aimed to extend previous literature by giving an account of the emergence and endurance of psychological changes through participation in an environmental campaign. In the context of the campaign, we present a qualitative analysis of a longitudinal panel study with quantitative measures of interaction and amount of change. In addition, we explore the processes of endurance of these changes illustrated through a case study of consumer behaviour and consumption attitudes." | People in the community (self-defined activists and locals) | Ethnography | individual interviews | 18 months | 28 | 196 interviews |
| Walker, K. C., Valentiner, L. S., & Langberg, H. (2018). Motivational factors for initiating, implementing, and maintaining physical activity behavior following a rehabilitation program for patients with type 2 diabetes: A longitudinal, qualitative, interview study. Patient Preference and Adherence, 12, 145-152. | Denmark | "The primary objective of the present study is to address the gap in knowledge on motivational changes over time to improve adherence to physical activity from the initiation of behavioral change in a rehabilitation program, to implementation of physical activity outside the program, and, finally, to maintenance of physical activity behavior in everyday life." | Patients (patients with type 2 diabetes) | Qualitative longitudinal research | Individual interviews | one year | 5 | Unclear, probably 10 interviews |
| Walker-Williams, H. J., & Fouché, A. (2018). Resilience enabling processes and posttraumatic growth outcomes in a group of women survivors of childhood sexual abuse. Health SA Gesondheid, 23, 1-9. | South Africa | "To explore emerging resilience processes and posttraumatic growth outcomes in women survivors of childhood sexual abuse after attending the S2T [programme entitled S2T denoting from Survivor to Thriver]" | People in the community (women survivors of childhood sexual abuse) | not described | Individual interviews, and drawings | about 12 months | 8 | Unclear |
| Wang, C.-L., Kuo, L.-M., Chiu, Y.-C., Huang, H.-L., Huang, H.-L., Hsu, W.-C., Lu, C.-H., Huang, T.-H., Huang, S., & Shyu, Y.-I. L. (2018). Protective preparation: A process central to family caregivers of persons with mild cognitive impairment. International Psychogeriatrics, 30(3), 375-384. | Taiwan | "Therefore, the purpose of this study was to explore and develop a theoretical model to explain the longitudinal changes in the caregiving process for family caregivers of persons with MCI [mild cognitive impairment] in Taiwan." | Family members (family caregivers of persons with MCI) | grounded theory | individual interviews | 2 years | 13 | 42 interviews |
| Weatherred, J. L. (2017). Framing child sexual abuse: A longitudinal content analysis of newspaper and television coverage, 2002-2012. Journal of Child Sexual Abuse, 26(1), 3-22. | US | "This study seeks to quantitatively explore how the media has framed CSA [Child Sexual Abuse], specifically whether it is framed as an individual or a societal level problem, from 2002 to 2012. The first research question seeks to establish the frequency of CSA news stories throughout the time period. RQ1: How often do news stories about CSA appear in U.S. television and newspaper coverage? Because previous research indicated a spike in news coverage that could be attributed to the Catholic Church, a quantitative measurement ofthis coverage was included in the coding sheet. The articles that were about Sandusky, the Catholic Church, the Boy Scouts, or other categories were coded. Therefore, the following research question is posed: RQ2: How much news coverage is about these specific CSA scandals? The next research question seeks to establish the nature of the news coverage, specifically if it is focused on individual or societal causes and solutions. RQ3: Is news coverage about CSA focused on individual or societal level causes and solutions? The fourth question seeks to determine whether differences in coverage of CSA exist between newspapers and television. RQ4: Do newspapers and television differ in whether they focus more on individual-level or societal causes and solutions for CSA?" In order to determine whether there have been changes in how the news media frame CSA, the following research question was posed: RQ5: Has the media’s framing of responsibility shifted from individual- to societal-level causes and solutions over time? | Others (US national news organizations) | not described | Documents (e.g., articles in daily press) | 10 years | 8 medias (TV-channels and news papers) | 503 newspaper articles |
| Weiser, S. D., Hatcher, A. M., Hufstedler, L. L., Weke, E., Dworkin, S. L., Bukusi, E. A., Burger, R. L., Kodish, S., Grede, N., Butler, L. M., & Cohen, C. R. (2017). Changes in health and antiretroviral adherence among HIV-infected adults in Kenya: Qualitative longitudinal findings from a livelihood intervention. AIDS & Behavior, 21(2), 415-427. | Kenya | "Here we report on results of a longitudinal qualitative study that was carried out simultaneously alongside the trial and aimed to understand how and why the intervention may have affected participant health." | Patients (people living with HIV) | Qualitative longitudinal research | individual interviews | 12 months | 74 | 117 interviews |
| Wenham, A., Atkin, K., Woodman, J., Ballard, K., & MacPherson, H. (2018). Self-efficacy and embodiment associated with Alexander Technique lessons or with acupuncture sessions: A longitudinal qualitative sub-study within the ATLAS trial. Complementary Therapies in Clinical Practice, 31, 308-314. | UK | "The ATLAS trial [1] has provided a useful platform from which to explore the participants' experiences and their perspectives of potential ways in which the changes in clinical outcomes came about. In particular, we aimed to explore in depth the role of learning, self-efficacy and self-care in sustaining benefits over the longer term." | Patients (participants with chronic neck pain who attended Alexander Technique lessons or acupuncture sessions) | Qualitative longitudinal research | individual interviews | 6 months | 30 | 56 interviews |
| Werner-Lin, A., Ersig, A. L., Mueller, R., Young, J. L., Hoskins, L. M., Desai, R., & Greene, M. H. (2018). Catalysts towards cancer risk management action: A longitudinal study of reproductive-aged women with BRCA1/2 mutations. Journal of Psychosocial Oncology, 36(5), 529-544. | US | "The present study sought to identify factors that shape risk management planning, life experiences that shape the capacity or decision about when, how, or whether to enact plans, and the psychosocial impact of those adaptions. Towards this end, we integrated multiple data points over time with BRCA mutation carriers of reproductive age." | Patients (women with increased cancerrisk) | Qualitative longitudinal research | Individual interviews | 2 to 3.5 years | 12 | 25 interviews |
| Whiffin, C. J., Baker, D., Henshaw, L., Nichols, J. J., & Pyer, M. (2018). "Am I a student or a healthcare assistant?" A qualitative evaluation of a programme of prenursing care experience. Journal of Advanced Nursing, 74(11), 2610-2621. | UK | "To examine the experiences of prenursing HCAs during a 6‐month programme of prenursing care experience. Determine the benefit of the experience to the individual participant and their ambition to become a Registered Nurse. • Examine how the experience informs participant's values and behaviours in clinical practice. • Identify key strengths and challenges in the delivery of formal programmes of prenursing care experience." | Health care providers (health care asisstants in prenursinng positions) | constructivist approach & qualitative longitudinal research | Focus group interviews | 6 months | 23 | 12 focus groups |
| Wiklund, M., Ahlgren, C., & Hammarström, A. (2018). Constructing respectability from disfavoured social positions: Exploring young femininities and health as shaped by marginalisation and social context. A qualitative study in Northern Sweden. Global Health Action, 11, 1519960. | Sweden | "The aim of this research was to elucidate constructs of femininities in relation to structuring living conditions and expressions of health in Northern Swedish women. The time period of interest was the transition from unemployed teenagers to young adults in a social context of high unemployment and societal change across the critical ‘school-towork-transition’ life course period." | People in the community (youth not in employment, education or training) | Qualitative longitudinal research | individual interviews | 15 years | 7 | 23 interviews |
| Winkel, A. F., Robinson, A., Jones, A. A., & Squires, A. P. (2019). Physician resilience: A grounded theory study of obstetrics and gynaecology residents. Medical Education, 53(2), 184-194. | US | "to create a conceptual model of resiliencein the context of OBGYN [obstetric and gynaecology] residency." | Students (obstetrics and gynaecology residents in postgraduate year) | Grounded theory | Individual interviews | 3 to 6 months | 18 | 36 interviews |
| Woodgate, R. L., Zurba, M., Edwards, M., Ripat, J. D., & Rempel, G. (2017). The embodied spaces of children with complex care needs: Effects on the social realities and power negotiations of families. Health & Place, 46, 6-12. | Canada | "This paper presents research findings that advance knowledge around the power and agency families with children with complex care needs (CCN). Our conceptual framework uses concepts from geography towards situating the experiences and social realities of family carers within the ‘embodied space of care’." (from abstract) | Several: Patients and family members (Children with complex care needs, their parents, and siblings) | ethnography & qualitative longitudinal research | Individual interviews and photovoice | 3 years | 92 | Unclear, at least 89 interviews |
| Wu, F. K. Y., Shek, D. T. L., Chai, C. W. Y., & Zhu, J. X. Q. (2019). Well-being of university students in Hong Kong: A longitudinal case study. International Journal of Child & Adolescent Health, 12(1), 99-107. | Hong Kong | "...to investigate the differences of views and suggest how these influences have influenced the understanding and perception of the assistance of GUR [general university requirements]." | Students (freshmen at university) | Case study | individual interviews, informal meetings, and field notes | about one year | 42 | 84 interviews and 42 one page summary field notes |
| Yalcin Arslan, F. (2019). Reflection in pre-service teacher education: Exploring the nature of four EFL pre-service teachers' reflections. Reflective Practice, 20(1), 111-124. | Turkey | "this study assessed the nature of student-teachers’individual reflections throughout the phases of school experience, teaching practice, and entry into the workplace. For this purpose, structured reflection was employed in order togain more thorough knowledge regarding this matter. [...] (1) What is the nature of EFL pre-service teachers’reflections during their teaching practicum? (2) What is the nature of EFL teachers’reflection upon their entry into the workplace?" | Several: Students and teachers (student and teachers at university) | Case study | Individual interviews | 3 months | 4 | 16 intervews |
| Yeh, E. A., Chiang, N., Darshan, B., Nejati, N., Grover, S. A., Schwartz, C. E., Slater, R., & Finlayson, M., for the Pediatric MS Adherence Study Group (2019). Adherence in Youth With Multiple Sclerosis: A Qualitative Assessment of Habit Formation, Barriers, and Facilitators. Qualitative Health Research, 29(5), 645-657. | US and Canada | "Therefore, we sought to understand the social and lifestyle factors that influence medication adherence in youth with MS, from the perspective of the youth, over time." | Patients (youth with MS) | Qualitative longitudinal research | Observations (e.g., transcripts from motivational interview sessions during the intervention) | 3 months | 28 | 84 transcripts |
| Young, J. L., Werner-Lin, A., Mueller, R., Hoskins, L., Epstein, N., & Greene, M. H. (2017). Longitudinal cancer risk management trajectories of BRCA1/2 mutation-positive reproductive-age women. Journal of Psychosocial Oncology, 35(4), 393-408. | US | "The current study examined data from follow-up interviews with a subset of Hoskins’ (2010) original cohort. We identified whether and how risk perceptions, management strategies, and family planning decisions articulated at baseline were put into practice. The goals were to learn how closely participants followed their initial plan and what factors, including life events, influenced their final decisions." | Patients (BRCA1/ 2 mutation-positive women) | grounded theory & qualitative longitudinal research | Individual interviews | 3 years | 12 | 24 interviews |
| Zadvinskis, I. M., Garvey Smith, J., & Yen, P.-Y. (2018). Nurses' experience with health information technology: Longitudinal qualitative study. JMIR Medical Informatics, 6(2), e38-e38. | US | "This was a longitudinal qualitative study that explored medical-surgical nurse perceptions of HIT [health information technology] implementation over time. We interviewed nurses three times after EHR and bar code medication administration (BCMA) implementation to capture evolving adaptation of their perceptions and behaviors in this specific job role. The objective of the study was to explore nurses’ experience of HIT implementation, and how they adapted their perceptions and behavior to HIT upgrades and optimization over time" | Health care providers (nurses) | Phenomenology & qualitative longitudinal research | individual interviews | 15 months | 19 | 30 interviews |
| Zuurmond, M., Nyante, G., Baltussen, M., Seeley, J., Abanga, J., Shakespeare, T., Collumbien, M., & Bernays, S. (2019). A support programme for caregivers of children with disabilities in Ghana: Understanding the impact on the wellbeing of caregivers. Child: Care, Health & Development, 45(1), 45-53. | Ghana | "...to study the interplay of various factors at different levels of a social system (family, support group, and community) affecting changes in the caregiver. [...] The research questions addressed in this paper are to explore (a) in what ways the intervention impacted upon the caregiver wellbeing and (b) the change process that caregivers engaged in, in order to inform future strengthening of the intervention." | Family members (care-givers to children with celeral palsy) | Qualitative longitudinal research | Individual interviews | 12 months | 15 | 38 interviews |
